# A model of the onset of the senescence associated secretory phenotype after DNA damage induced senescence

**DOI:** 10.1371/journal.pcbi.1005741

**Published:** 2017-12-04

**Authors:** Patrick Meyer, Pallab Maity, Andre Burkovski, Julian Schwab, Christoph Müssel, Karmveer Singh, Filipa F. Ferreira, Linda Krug, Harald J. Maier, Meinhard Wlaschek, Thomas Wirth, Hans A. Kestler, Karin Scharffetter-Kochanek

**Affiliations:** 1 Department of Dermatology and Allergic Diseases, University of Ulm, Germany; 2 Aging Research Center (ARC), University of Ulm, Germany; 3 Institute of Medical Systems Biology, University of Ulm, Germany; 4 International Graduate School in Molecular Medicine, University of Ulm, Germany; 5 Institute of Physiological Chemistry, University of Ulm, Germany; University of Connecticut Health Center, UNITED STATES

## Abstract

Cells and tissues are exposed to stress from numerous sources. Senescence is a protective mechanism that prevents malignant tissue changes and constitutes a fundamental mechanism of aging. It can be accompanied by a senescence associated secretory phenotype (SASP) that causes chronic inflammation. We present a Boolean network model-based gene regulatory network of the SASP, incorporating published gene interaction data. The simulation results describe current biological knowledge. The model predicts different in-silico knockouts that prevent key SASP-mediators, IL-6 and IL-8, from getting activated upon DNA damage. The NF-κB Essential Modulator (NEMO) was the most promising *in-silico* knockout candidate and we were able to show its importance in the inhibition of IL-6 and IL-8 following DNA-damage in murine dermal fibroblasts *in-vitro*. We strengthen the speculated regulator function of the NF-κB signaling pathway in the onset and maintenance of the SASP using *in-silico* and *in-vitro* approaches. We were able to mechanistically show, that DNA damage mediated SASP triggering of IL-6 and IL-8 is mainly relayed through NF-κB, giving access to possible therapy targets for SASP-accompanied diseases.

## Author summary

The senescence associated secretory phenotype is developed by cells undergoing permanent cell cycle arrest. This phenotype is characterized by the secretion of a variety of factors that facilitate tissue breakdown and inflammation and is therefore theorized to, in part, be causal for aging and age-related diseases. In recent years the SASP has been implicated in a variety of chronic inflammatory diseases. Due to these advances, it is imperative to better understand the dynamics of this cellular phenotype and to find ways to disrupt it. We have developed a Boolean network incorporating the major signaling pathways of the SASP that allows us to specifically investigate interactions of the pathways and genes involved. We validated our model by reliably reproducing published data on the SASP. We utilized our model to uncover components that directly control the detrimental effects of the senescence associated secretory phenotype that are largely caused by IL-6 and IL-8, two major factors of the SASP in establishing and spreading senescence as well as causing local inflammation. In subsequent in-vitro experiments, we were able to verify our computational results and could suggest NEMO as one potential target for therapy of SASP-related diseases.

## Introduction

Age-related diseases can be held accountable for the major part of morbidity and mortality in an ageing population. Additionally they cause a large proportion of yearly health costs [1]. Cellular senescence is one of the most prominent events that is likely to contribute to ageing. It refers to the irreversible cell cycle arrest that is essential when cells encounter detrimental changes. Once in permanent arrest, these cells are normally cleared by the immune system before they are able to do any harm to the organism [2]. However, some of these cells persist and develop a secretory phenotype releasing a variety of factors among which pro-inflammatory cytokines, chemokines and extracellular matrix degrading proteases are included. Together these shape the senescent-associated secretory phenotype or SASP [3–5].

While the SASP can cause chronic inflammation in tissue, it can also reinforce senescence in autocrine and paracrine manner [6, 7]. This feature of the SASP not only keeps senescent cells in their growth arrested states but it promotes senescence spreading to healthy bystander cells. Therefore, the SASP contributes to the accumulation of senescent cells during ageing, but also supports the emergence of age-related chronic diseases and tissue dysfunctions by elevating inflammatory processes [6, 8]. Major soluble factors that facilitate this bystander-infection of healthy cells are IL-6 and IL-8. Both have been shown to be important in the maintenance and spreading of oncogene- and DNA-damage-induced senescence [3]. Also, both have been shown to be highly overexpressed by senescent cells and are known to locally and systemically play important roles in the regulations of a variety of processes in the aging body [3, 4, 9]. IL-6, in fact, most likely contributes to organ dysfunction during aging thus promoting frailty [8].

To allow for a deeper understanding of the SASP and the dynamics of its complex interactions a computational model of the Regulatory Network (RN) [10] and subsequent simulations can be insightful. RNs can be described by different mathematical models such as differential equations, Bayesian networks, and Boolean networks among others [11]. The Boolean network model [12, 13], as opposed to other model approaches, can be based on qualitative knowledge only. In gene-gene interaction, for example, the expression of a gene is regulated by transcription factors binding to its regulatory regions. The activation of a gene follows a switch-like behavior depending on the concentration of its transcription factors. This behavior allows common approximation of the possible states of a gene to be active or inactive [14, 15]. Ultimately, this can be encoded as Boolean logical values: true (“1”) or false (“0”). The interactions between genes, e.g. whether a factor acts as an activator, repressor or both can be described by functions. These Boolean functions are the basis to simulate dynamic behavior, i.e. changes over time. As every regulatory factor has two possible states (active or inactive) in a Boolean network model, 2^x^ possible state combinations (i.e. gene activation patterns) exist for x genes. For any activation pattern, iterative updates of genes in the network through consecutive application of the Boolean rules eventually lead to sequences of gene activation patterns that are time-invariant, called attractors. These attractors can correspond to observed expression profiles of biological phenotypes or can be used to create hypotheses to further evaluate in wet-lab experiments [16, 17]. Different update strategies for the Boolean functions exist. Using a synchronous update strategy means applying all Boolean functions simultaneously, also assuming that regulatory factors interact independently of one another and that their interaction has a similar time scale resolution. Relaxing these assumptions leads to the concept of asynchronous updates where each Boolean function of is updated separately one at a time in any order. This allows a more direct modelling of different time scales. The asynchronous update strategy also usually generates trajectories that are different from those of synchronous Boolean networks. The state transition graph of an asynchronous Boolean network becomes a Markov chain which requires the additional definition of transition probabilities in each node of the state graph. Interestingly, point attractors (those with one state) in asynchronous Boolean networks are the same as those in synchronous Boolean networks. However, these networks can also show loose/complex attractors [18] which are part of active research [19, 20]. Another extension of Boolean networks are probabilistic Boolean networks, which may define more than one Boolean function for regulatory factors where each function has a specific probability to be chosen for update. Although this concept may closer represent a biological system, it again requires parameter estimation for the probabilities. However, estimation of the probabilities naturally demands large amounts of interaction specific data which is, for larger networks, neither economically, nor experimentally viable. In our case, we decided to focus on synchronous Boolean networks, partly due to their proven usability, and their ability to reveal key dynamical patterns of the modelled system. However, to strengthen our models’ hypothesis, we additionally performed in-silico experiments with an asynchronous update scheme (S1 Text).

Synchronous Boolean networks have been used to model the oncogenic pathways in neuroblastoma [21], the hrp regulon of Pseudomonas syringae [22], the blood development from mesoderm to blood [23], the determination of the first or second heart field identity [24] as well as for the modeling of the Wnt pathway [25]. The qualitative knowledge base that is necessary to reconstruct [26] a Boolean network model consists mostly of reports on specific interactions that describe local regulation of genes or proteins. Boolean network models utilize this knowledge about local regulations to reconstruct a first global mechanistic model of SASP. In summary, such a model allows to generate hypotheses about regulatory influences on different local interactions. These interactions, in turn, can be tested in wet-lab in order to validate the generated hypothesis and assess the accuracy of the proposed model.

Here, we present a regulatory Boolean network of the development and maintenance of senescence and the SASP incorporating published gene interaction data of SASP-associated signaling pathways like IL-1, IL-6, p53 and NF-κB. We simulated the model and retrieved steady states of pathway interactions between p53/p16^INK4A^ steered senescence, IL-1/IL-6 driven inflammatory activity and the emergence and retention of the SASP through NF-κB and its targets. This Boolean network enables the highlighting of key players in these processes. Simulations of knock-out experiments within this model go in line with previously published data. The subsequent validation of generated *in-silico* results *in-vitro* was done in murine dermal fibroblasts (MDF) isolated from a murine NF-κB Essential Modulator (NEMO)-knockout system in which DNA damage was introduced. The NEMO knockout inhibits IL-6 and IL-8 homologue mRNA expression and protein secretion in MDFs after DNA damage *in-vitro*, possibly enabling at least a lowering of the contagiousness for neighboring cells and the pro-tumorigenic potential of the SASP. The model presented in this article allows a mechanistic view on interaction between the proinflammatory and DNA-damage signaling pathways and thereby helps to gain insights into the dynamics of the SASP. Furthermore, it enables to generate extensive hypotheses about possible knockout targets that can be experimentally tested and verified *in-vitro*. To the best of our knowledge, this report is the first one that combined *in-silico* simulation of the SASP with its laboratory based experimental validation.

## Results

### The network model exhibits stable states for cell cycle progression and senescence

The reconstruction of a Boolean network model for SASP requires screening for many candidate interactions in published literature and data. Although the model, after reconstruction, may be reduced in the number of components [20, 27, 28], it would potentially hide some of the interaction targets and regulatory factors with regard to the signaling cascade. The regulatory factors defined in this model are beneficial if one wants to extend the model and include additional related signaling pathways. The subsequent model must accurately correspond to the current understanding of the process at hand, i.e., able to predict well-known phenotypes of SASP. Biological phenotypes represent a long-term behavior of a biological system based on interaction of regulatory factors. In the same sense, attractors are the long-term behavior of a Boolean network model based on the Boolean rules of modelled regulatory factors. Hence, there is a natural correspondence between biological phenotypes and attractors in the Boolean network. In the following, we use figures that depict the signaling cascade towards an attractor as well as the attractor itself. The interpretation of these attractors in the context of SASP further allows generation of hypotheses that can be tested in a biological system.

**Fig 1:**
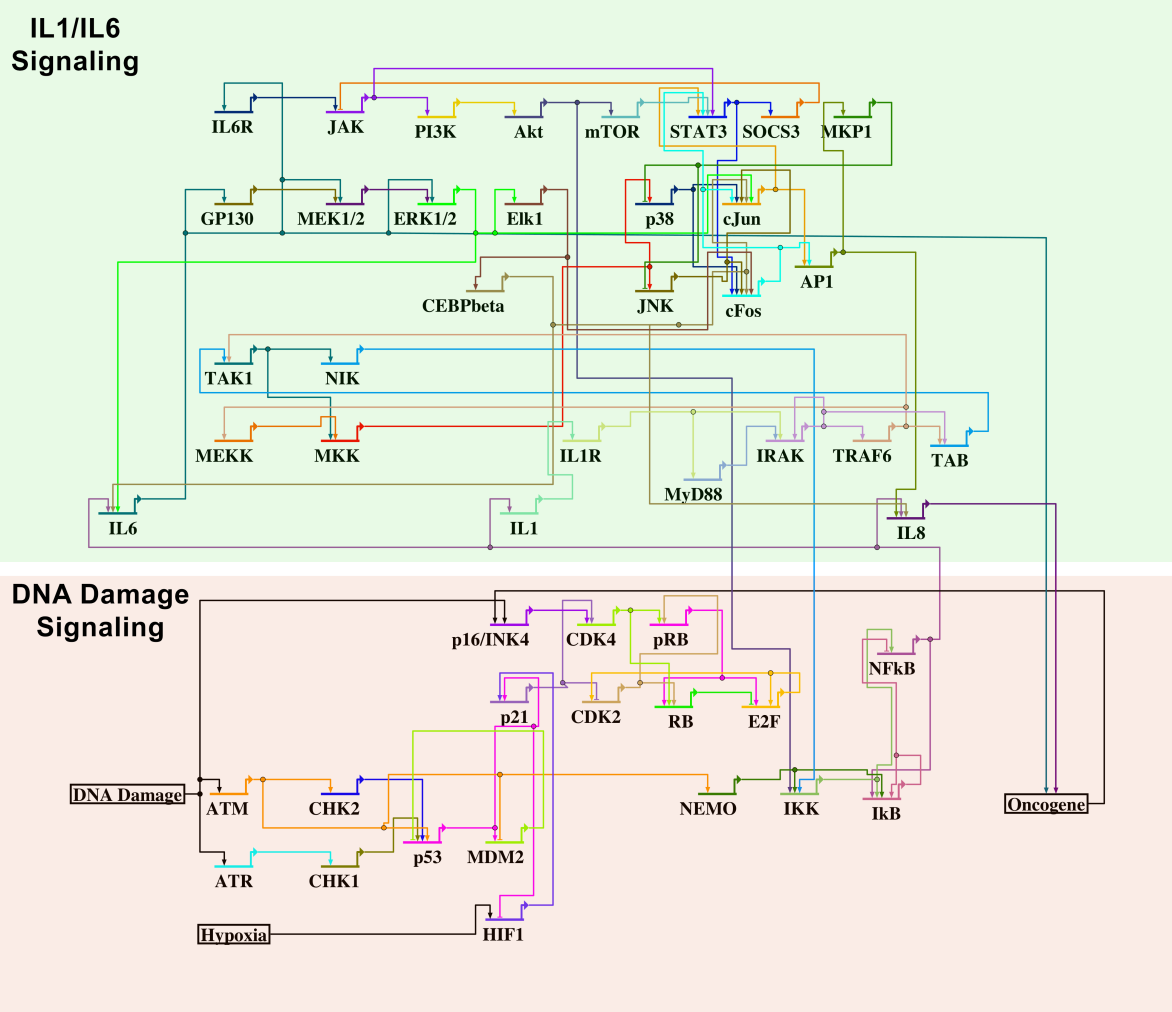
Boolean network for gene regulation during cell cycle progression and the onset of cell cycle arrest after DNA damage. The overview shows the network wiring of the known gene regulations during DNA damage with a focus on the DNA damage repair/cell cycle arrest signaling. Cell cycle arrested cells over time show a tendency to develop a secretory phenotype that causes them to secrete high amounts of proinflammatory factors that can negatively influence neighboring cells. Major signaling pathways of these factors are included in this overview and in the Boolean network. Arrows indicate gene activation and inhibition is depicted as bar head. However, the interaction may be more complex and the corresponding Boolean rules are given in Table 1.

The information for the reconstruction of these networks was collected from published data. An overview of the genes incorporated in this model and their interaction can be found in Fig 1. The corresponding Boolean rules are listed in Table 1. The network depicts processes following a cell cycle arrest inducing action, such as DNA damage and other cellular stresses. Here, we analyze SASP under strong DNA damage and do not distinguish between different levels of DNA damage.

**Table 1:** Boolean network for gene regulation during cell cycle progression and the onset of cell cycle arrest after DNA damage. Boolean Rules using operators “&” (logical and), “|” (logical or) and “¬” (logical not).

DNA Damage/Senescence signaling		
Regulatory Factor at time ***t+1***	Boolean rule update given regulatory factor state at time ***t***	
	*DNA Damage*, *Defective Telomeres*, *etc*.	
DNAD	DNAD	This rule serves as an input signal to any kind of severe DNA damage.
	*Oncogene induced senescence*	
Oncogene	IL8 | IL6	Active IL-6 or IL-8 signaling characterize the activation of Oncogene. Moreover, IL-6 and IL-8 also required for oncogene induced senescence [3].
Hypoxia	Hypoxia	Exogenous factor describing Hypoxia.
	*In presence of DNA damage*, *a cell activates regulatory factors ATR and ATM*, *which subsequently activate checkpoints CHK1 and CHK2*.	
ATM	DNAD	ATM is active in presence of DNA damage [57–59].
CHK2	ATM	ATM subsequently activates CHK2 [60].
ATR	DNAD	ATR is active in presence of DNA damage [57, 59].
CHK1	ATR	ATR subsequently activates CHK1 [61].
p53	(CHK2 | CHK1 | ATM) & (¬MDM2)	p53 can be activated by any of CHK1 [62], CHK2 [62, 63] or ATM [62, 64]. However, MDM2 is a strong inhibitor of p53 [62, 65].
HIF1	Hypoxia & (¬p53)	HIF1, which is active during Hypoxia [66], is inhibited by p53 [67].
p21	p53 | HIF1	p21 is activated by p53 [68] as well as by HIF1 [69].
CDK2	E2F & (¬p21)	CDK2 requires activation of E2F. p21 inhibits the CDK2 complex [68].
RB	¬(pRB | CDK4 | CDK2)	RB, which is active in its hypophosphorylated state (RB) is hyperphosphorylated and inactivated (pRB) by CDK4 and CDK2 [70–72].
pRB	(CDK4 | CDK2)	RB is phosphorylated (pRB) in presence of any cyclin dependent kinases CDK4 and CDK2 [70–72].
E2F	(pRB | E2F) & ¬RB	E2F is positively autoregulated and active in presence of hyperphosphorylated RB (pRB). Active RB, however, inhibts E2F [38].
MDM2	p53 & ¬ATM	p53 activates MDM2 [65, 73, 74], while ATM inhibits MDM2 [64].
p16INK4	Oncogene | DNAD	Activation of p16INK4 depends on either DNA damage or Oncogene or both [75].
CDK4	¬(p16INK4 | p21)	CDK4 is inhibited by p16INK4 [75] and p21 [68].
NEMO	DNAD	NEMO is activated by DNA damage [76, 77].
IKK	NEMO | NIK | Akt	IKK can be activated by any of NEMO [78], NIK [79] or Akt [80].
IkB	(NFkB |IkB) & ¬(IKK & NEMO)	IkB is activated NFkB complex or IkB itself [81]. IKK [82] and NEMO [83] together are required to inhibit IkB.
NFkB	IKK & ¬IkB	NFkB is activated by IKK, while inhibited by IkB [82, 83].
**IL-1 signaling**		
IL1	NFkB	IL1 is activated by NFkB [29, 30].
IL1R	IL1	IL1 binds to and activates IL1 receptor (IL1R) [84].
MyD88	IL1R	MyD88 is an adaptor molecule in IL1-IL1R pathway and bridging IL1R to the IRAK complex IL1R [84].
IRAK	IL1R | MyD88 | IRAK	IRAK is autoactivated [85, 86] and also is activated by IL1R [84, 86] and MyD88 [85, 87].
TRAF6	IRAK	TRAF6 is activated by IRAK [85].
TAB	(TRAF6 | IRAK)	TAB is activated by any of TRAF6 [88, 89] or IRAK [89].
TAK1	(TRAF6 | TAB)	TAK1 is activated by any of TRAF6 [88, 89] or TAB [90].
MEKK	TRAF6	MEKK is activated by TRAF6 [89].
MKK	(TAK1 | MEKK)	MKK is activated by any of TRAK1 [91, 92] or MEKK [93].
JNK	MKK & ¬MKP1	JNK is activated by MKK [94, 95] while is inhibited by MKP1 [96].
p38	MKK & ¬MKP1	p38 is activated by MKK [97] while inhibited by MKP1 [98].
cJun	(p38 | JNK | ERK1_2 | CEBPbeta) & cFos	cFos is required for the action of cJun and can be activated by any one of p38 [99, 100], JNK [101], ERK1_2 [102] or CEBPbeta [103].
cFos	p38 | JNK | Elk1 | CEBPbeta | STAT3	cFos can be activated by any one of p38 [104], JNK [104], Elk1 [103, 105, 106], CEBPbeta [103] or STAT3 [107].
AP1	cJun & cFos	AP1 complex consists of both cJun and cFos [104, 108].
MPK1	AP1	AP1 activates MPK1 [96, 109, 110].
IL8	NFkB | AP1 | CEBPbeta	IL8 is activated by anyone of NFkB [31, 111, 112], AP1 [31] or CEBPbeta [3] signals.
NIK	TAK1	NIK is activated by TAK1 [91, 92].
**IL-6 signaling**		
IL6	(NFkB | ERK1_2 | CEBPbeta)	IL6 is activated by anyone of NFkB [32, 33], ERK1_2 [113, 114] or CEBPbeta [3, 115] signals.
IL6R	IL6	IL6 binds to and activates IL6 receptor (IL6R) [88, 116].
GP130	IL6	GP130 is activated by IL6 [117, 118].
PI3K	JAK	PI3K is activated by JAK [119].
JAK	IL6R & ¬SOCS3	Active IL6 receptor (IL6R) activates JAK [117], while JAK is inhibited by SOCS3 [120].
Akt	PI3K	Akt is activated by PI3K [121, 122].
mTOR	Akt	mTOR is activated by Akt [123].
SOCS3	STAT3	SOCS3 is activated by STAT3 [124].
	*GP130*, *MEK1_2*, *and ERK1_2 together depend all on the activation of IsL6 to form a cyclic signaling cascade*	
MEK1_2	GP130 & IL6	MEK1_2 is activated by GP130 [116, 125] as well as IL6 [116].
ERK1_2	MEK1_2 & IL6	ERK1_2 is activated by MEK1_2 [126] and IL6 [127].
Elk1	ERK1_2	Elk1 is activated by ERK1_2 [128].
CEBPbeta	Elk1	CEBPbeta is activated by Elk1 [103].
STAT3	JAK | (cFos & cJun) | mTOR	STAT3 is activated by JAK [119] or mTOR [129]. Alternatively is can be activated in presence of both cFos and cJun [130].

**Fig 2:**
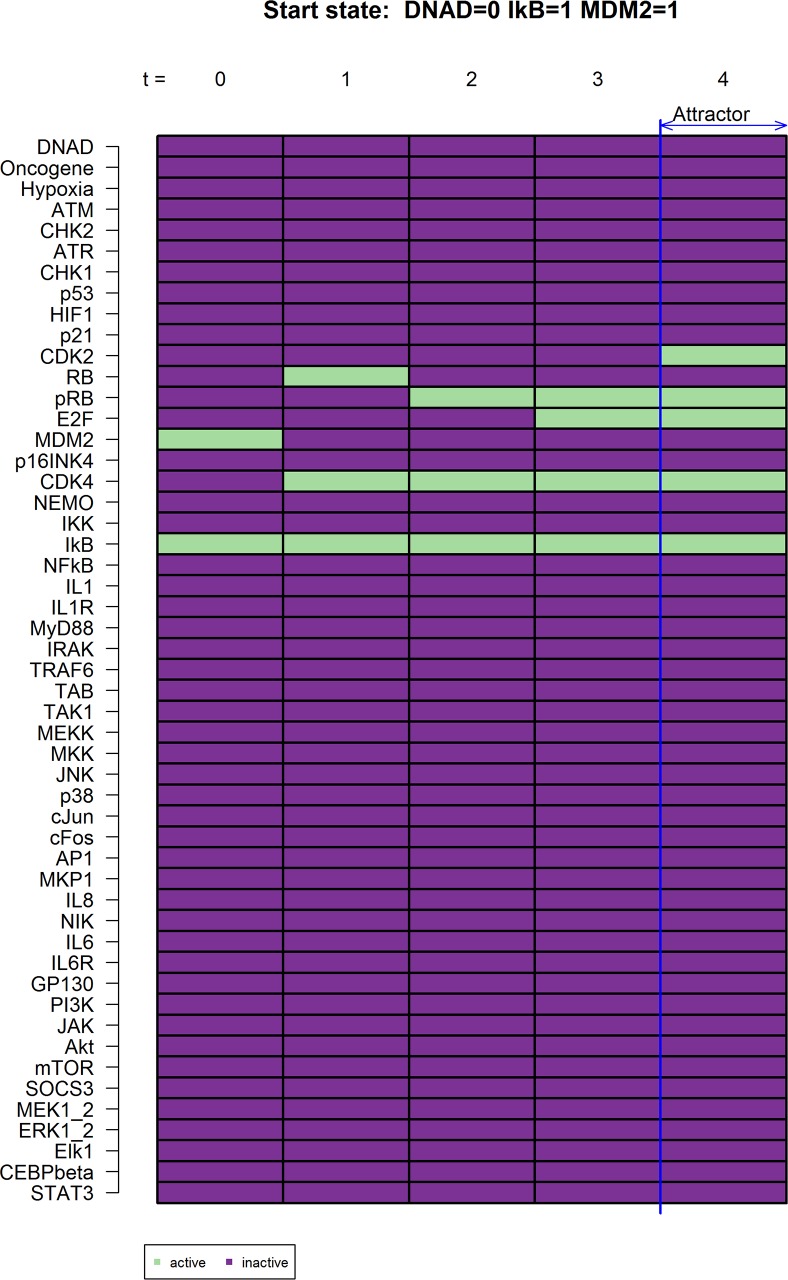
Naturally occurring network states. Without DNA damage the resulting network state is expected to show normal cell cycle progression. As shown here this includes the activation of CDK2 (t = 5) and CDK4 (t = 2) with a subsequent phosphorylation of RB (t = 3) leading to a release of E2F (t = 4) which will release the cell into cell cycle progression. The temporal sequence is shown as t = n. Active genes are shown as green, inactive genes as dark purple.

We first analyzed if our model can render steady states for cell cycle progression when there is no stress signal input. Our data show a normal cell cycle progression with active CDK2 and CDK4, as well as phosphorylated Rb and hence an active E2F. No other signaling pathways that are implemented in this model were activated which can be seen as normal cell cycle progression (Fig 2).

**Fig 3:**
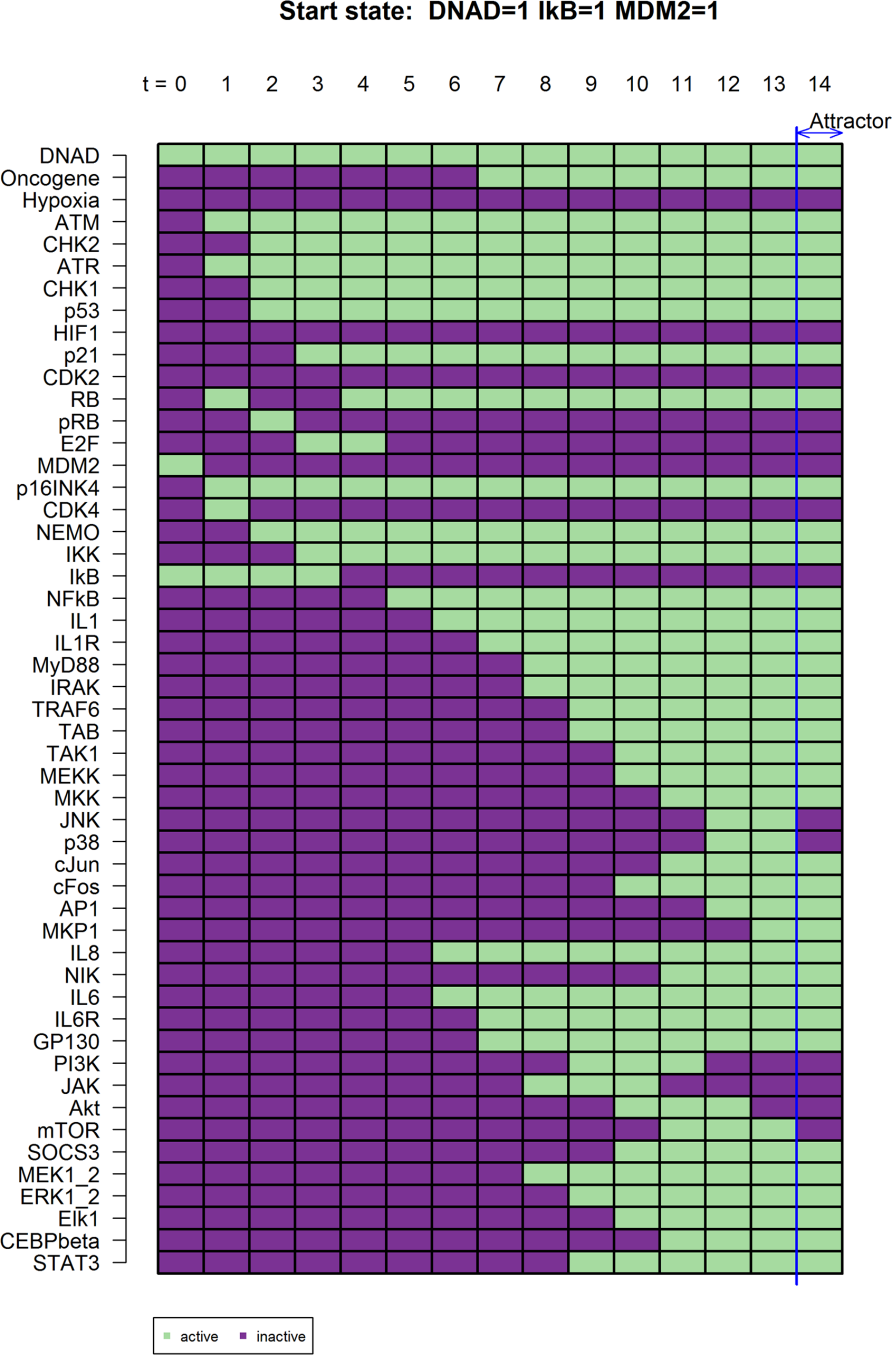
Naturally occurring network states upon DNA damage. Upon DNA damage the first response of the cell is the activation of ATM/ATR mediated DNA damage repair (t = 2) with a subsequent activation of p53- and p16-mediated cell cycle arrest (t = 3). The DNA damage signal is relayed by the DNA damage response through NEMO (t = 3) that in turn activates NF-κB signaling (t = 4) which will ultimately lead to the activation of IL-1, IL-6 and IL-8 signaling (t = 7). The temporal sequence is shown as t = n. Active genes are shown as green, inactive genes as dark purple.

Upon the outside signal DNA damage, we observe first the activation of the DNA damage response with a subsequent activation of p53 and p16^INK4A^ signaling, leading to a stop in cell cycle progression and at a later time point to permanent cell cycle arrest. Simultaneously NF-κB signaling gets activated by the DNA damage response through NEMO, giving rise to beneficial but also detrimental effects of NF-κB like the senescence associated secretory phenotype (Fig 3).

After entering p53/p21 and p16^INK4A^ mediated permanent cell cycle arrest upon DNA damage, the activation of NF-κB leads to an increase of IL-1, IL-6 as well as IL-8 expression among others [29–33]. Our model shows the direct activation of these cytokines and chemokines by NF-κB after its activation through the DNA damage response and NEMO (Fig 3).

### The Boolean network describes published knock-out and overexpression phenotypes

The NF-κB pathway has been studied extensively and there are knockout mice available for all proteins of the pathway, however some of them are embryonically lethal due to the importance of NF-κB signaling in regulating development and apoptosis. We therefore focused on published *in-vitro* knockout and overexpression phenotypes. IL-6 and IL-8 are extremely important in maintaining and spreading the SASP in an autocrine as well as paracrine fashion. Hence, we followed the question what knockouts and/or overexpressions the Boolean network model suggests to inhibit the expression of IL-6 and IL-8 under the assumption of existing DNA damage. These simulations are included in S1 Text.

**Fig 4:**
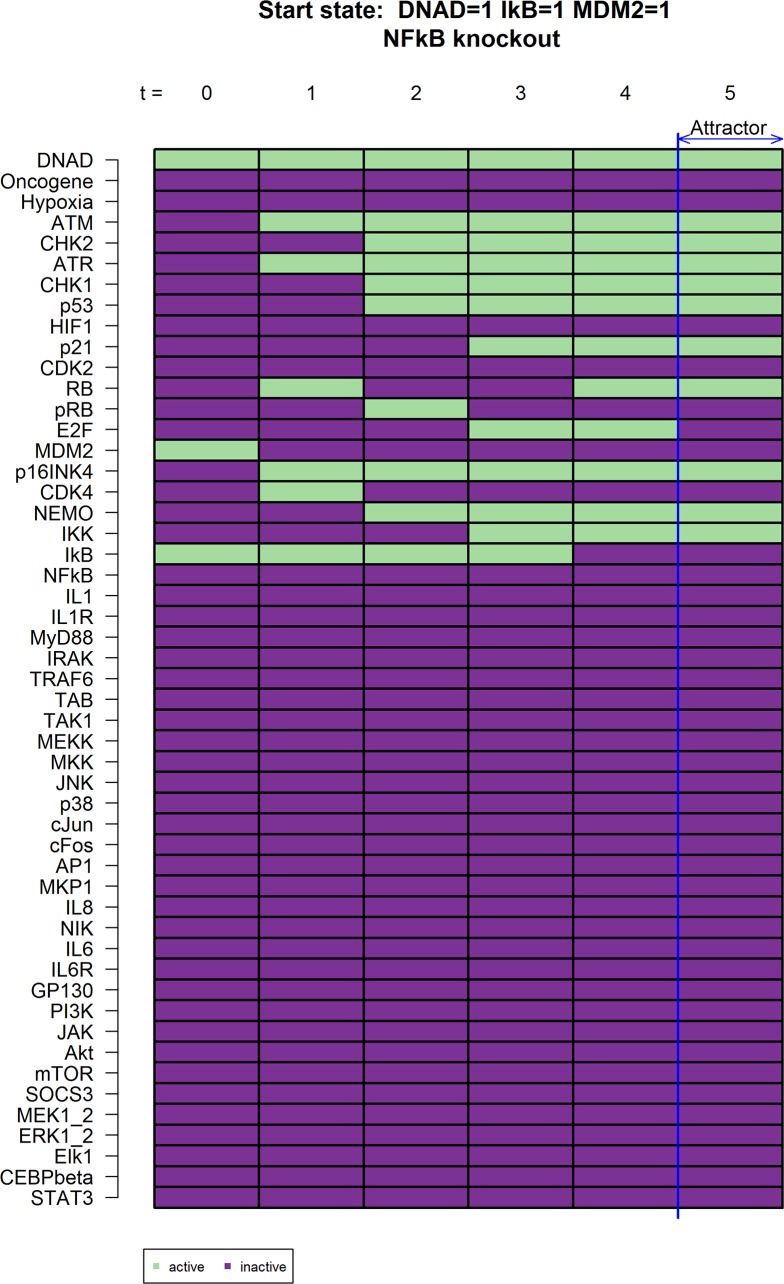
Knockouts that cause *in-silico* IL-6 and IL-8 inhibition for NFkB knockout. Network states present the gene activity of all genes in the model. Green boxes indicate gene activation while red boxes show gene inactivation. A knock-down or overexpression is simulated by setting a gene to 0 or 1, respectively. This simulation shows the time course of expected states after DNA damage with NF-κB switched off (NFkB = 0) which leads to an inhibition of proinflammatory signaling.

RelA binds with p50 to form a transcriptionally active heterodimer (called NFkB in this model). In its inactive state, it is bound with the inhibitor of kappa B (IκB) and resides in the cytoplasm. Upon NF-κB activation, the inhibitor is phosphorylated by the inhibitor of kappa B kinases (IKK) and degraded which releases the RelA/p50 heterodimer to translocate to the nucleus and regulate the transcription of target genes. To investigate the role of RelA on the expression of IL-8, we set NFkB = 0, simulating the ablation of the transcriptionally active heterodimer (Fig 4). The predictions of the model simulations are consistent with knock-out experiments where the absence of RelA caused a significant reduction in IL-8 production in human fibroblast (IMR-90) [7].

**Fig 5:**
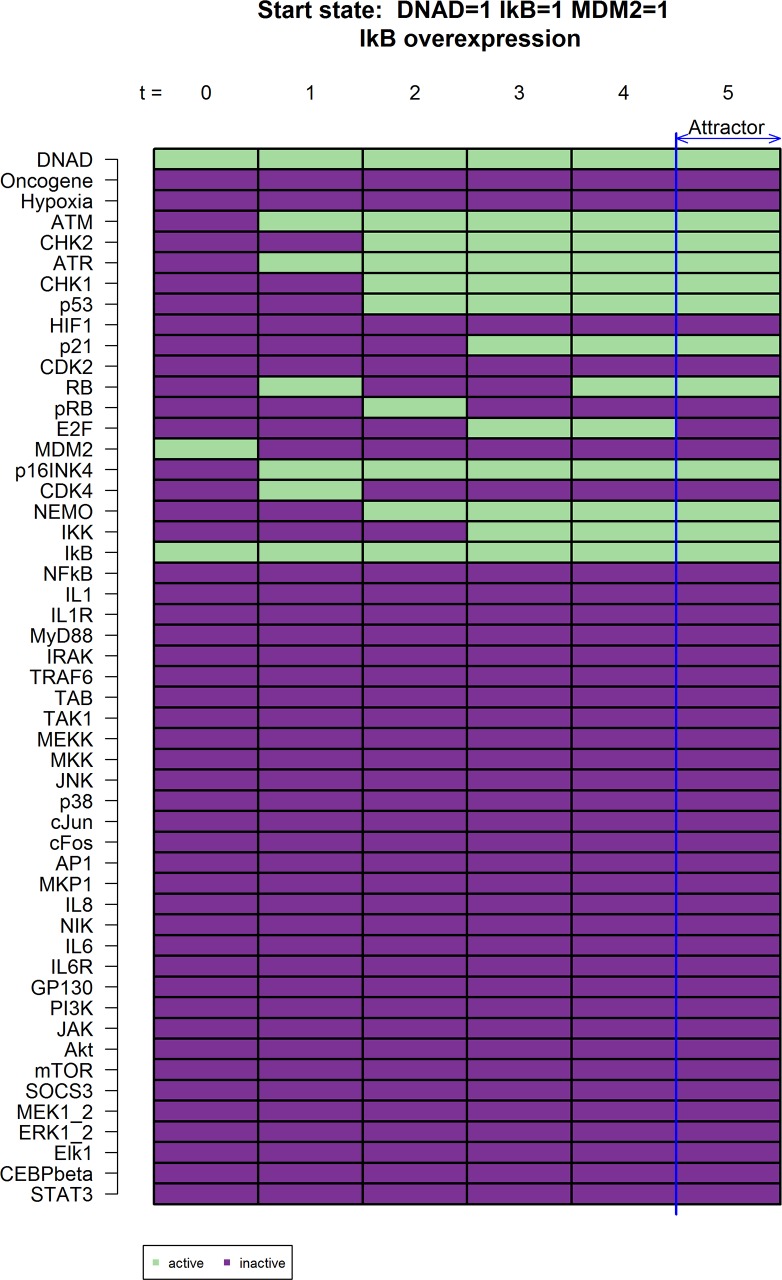
Knockouts that cause *in-silico* IL-6 and IL-8 inhibition for IkB overexpression. This simulation shows an overexpression of IκB (IkB = 1) showing a similar outcome as in Fig 4.

We also simulated the overexpression of IκB by constantly activating IκB (IkB = 1) and could show an effect comparable to the knock-out of RelA (Fig 5). In our model the overexpression of IκB leads to the inhibition of IL-8 and IL-6 expression which is in line with a previously published report, where the overexpression of a non-degradable IκBα completely abolishes IL-8 production, among other soluble factors, in human epithelial and cancer cell lines [34].

**Fig 6:**
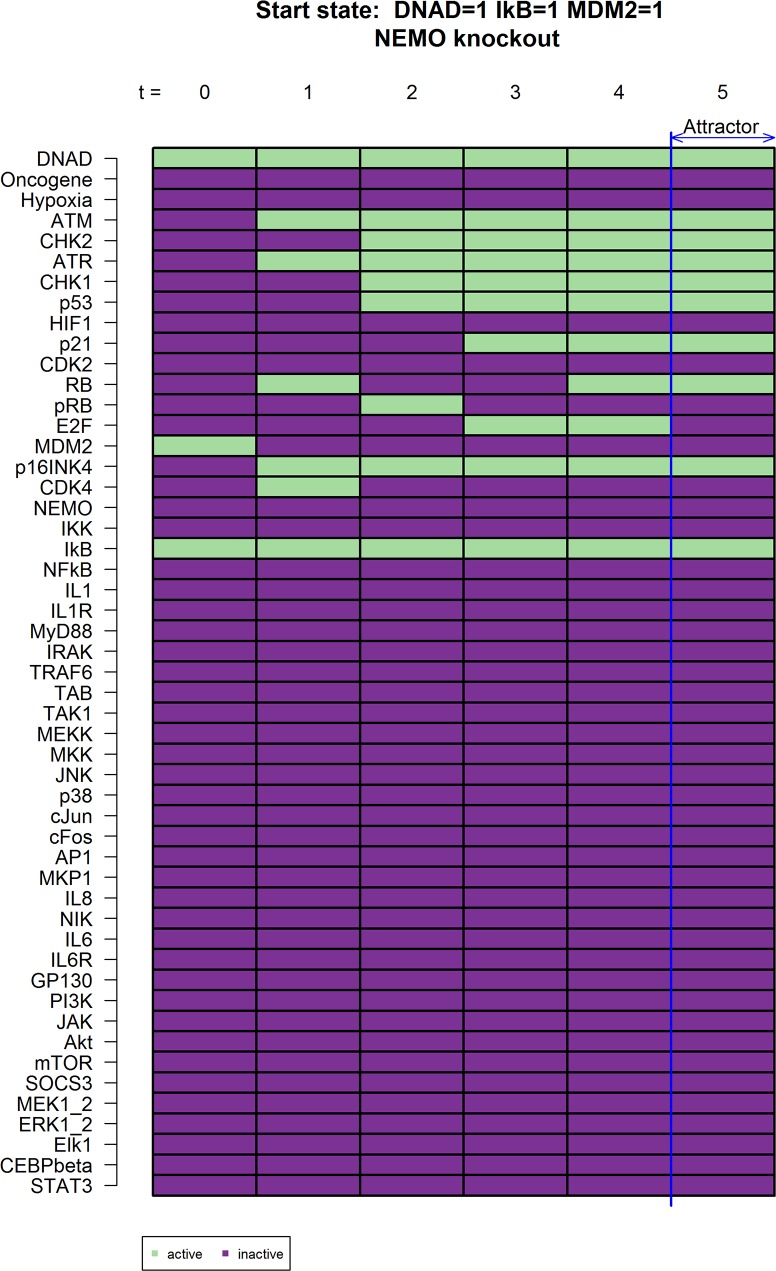
Knockouts that cause *in-silico* IL-6 and IL-8 inhibition for NEMO knockout. NEMO is switched off (NEMO = 0) preventing NF-κB signaling from being activated. The outcome is similar to the two previously described simulations in Figs 4 and 5.

Another promising knockout described by our network is inhibitor of nuclear factor kappa-B kinase subunit gamma also known as NEMO, which is able to prevent IL-6 and IL-8 expression after DNA damage activated the DNA damage repair apparatus and cell cycle progression has been stopped *in-silico* (Fig 6). In studies with murine NEMO knockout models it has already been shown that murine embryonic fibroblasts (MEFs) isolated from these mice show reduced NF-κB activity and IL-6 secretion upon stimulation with typical NF-κB activators like IL-1 and TNF [35].

### NEMO is essential for DNA damage triggered NF-κB activation

Apart from being important for the assembly of the IKK-complex, NEMO also acts as a shuttle relaying the ATM-mediated DNA damage apparatus to cellular response mechanisms. Upon DNA damage ATM can bind NEMO and trigger its translocation from the nucleus to the cytoplasm where it activates NF-κB signaling [36]. This in turn will help cells avoid clearance through apoptosis, increasing the number of long-term senescent cells in tissues and organs of the organism and might also increase and sustain the inflammatory potential of the SASP.

**Fig 7:**
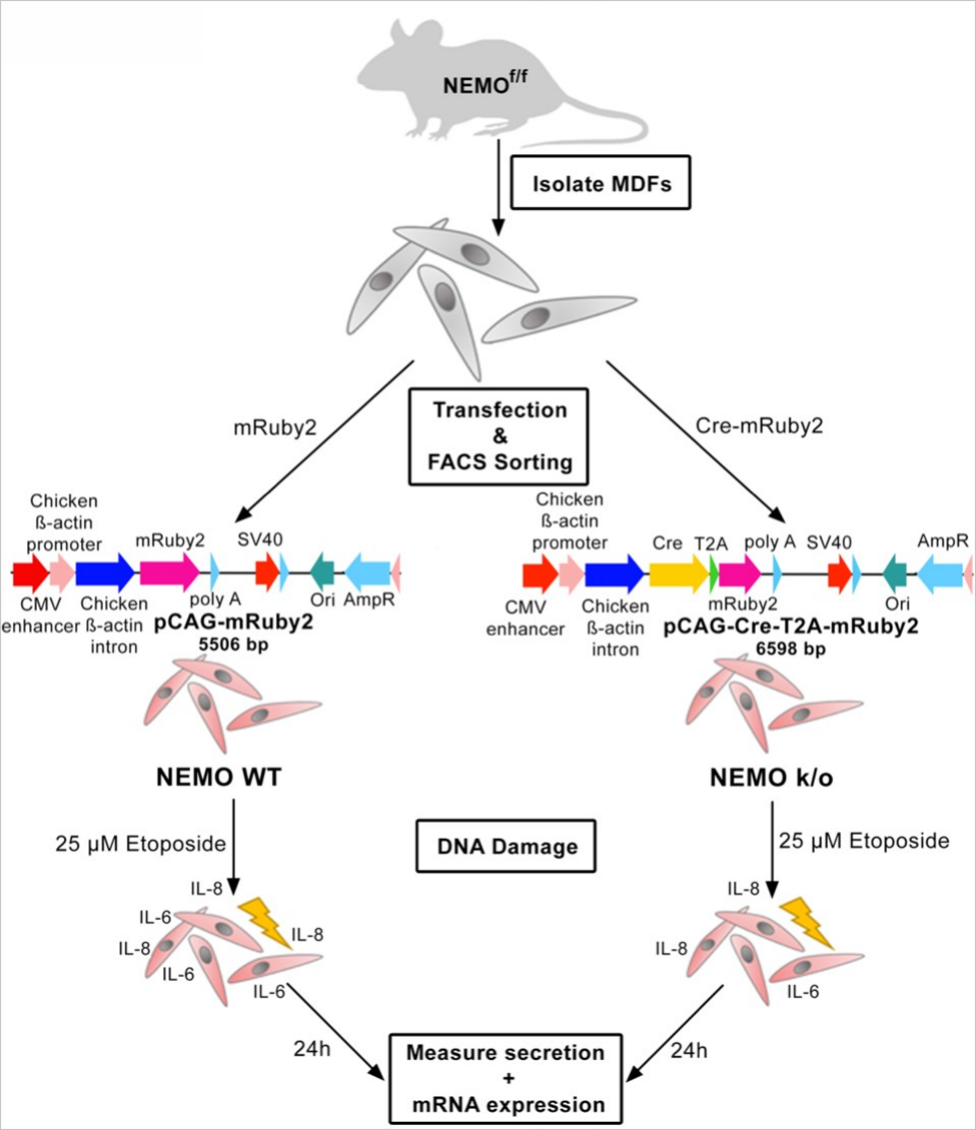
Schematic overview of the experimental workflow. Murine dermal fibroblasts (MDFs) are isolated from NEMO-floxed mice. After short expansion in cell culture these MDFs are transfected with pCAG-Cre-T2A-mRuby2 or pCAG-mRuby2, respectively. Because of mRuby2 expression, successfully transfected cells can be sorted by FACS. Cells transfected with pCAG-Cre-T2A-mRuby2 are knocked out for NEMO while pCAG-mRuby2 transfected cells are used as wildtype controls. After transfection cells are treated with 25 μM etoposide for 3 h to induce DNA damage. 24 h after treatment cell culture media is taken for ELISA measurement of secretion and cells are harvested for RNA isolation and subsequent RT-qPCR analysis.

**Fig 8:**
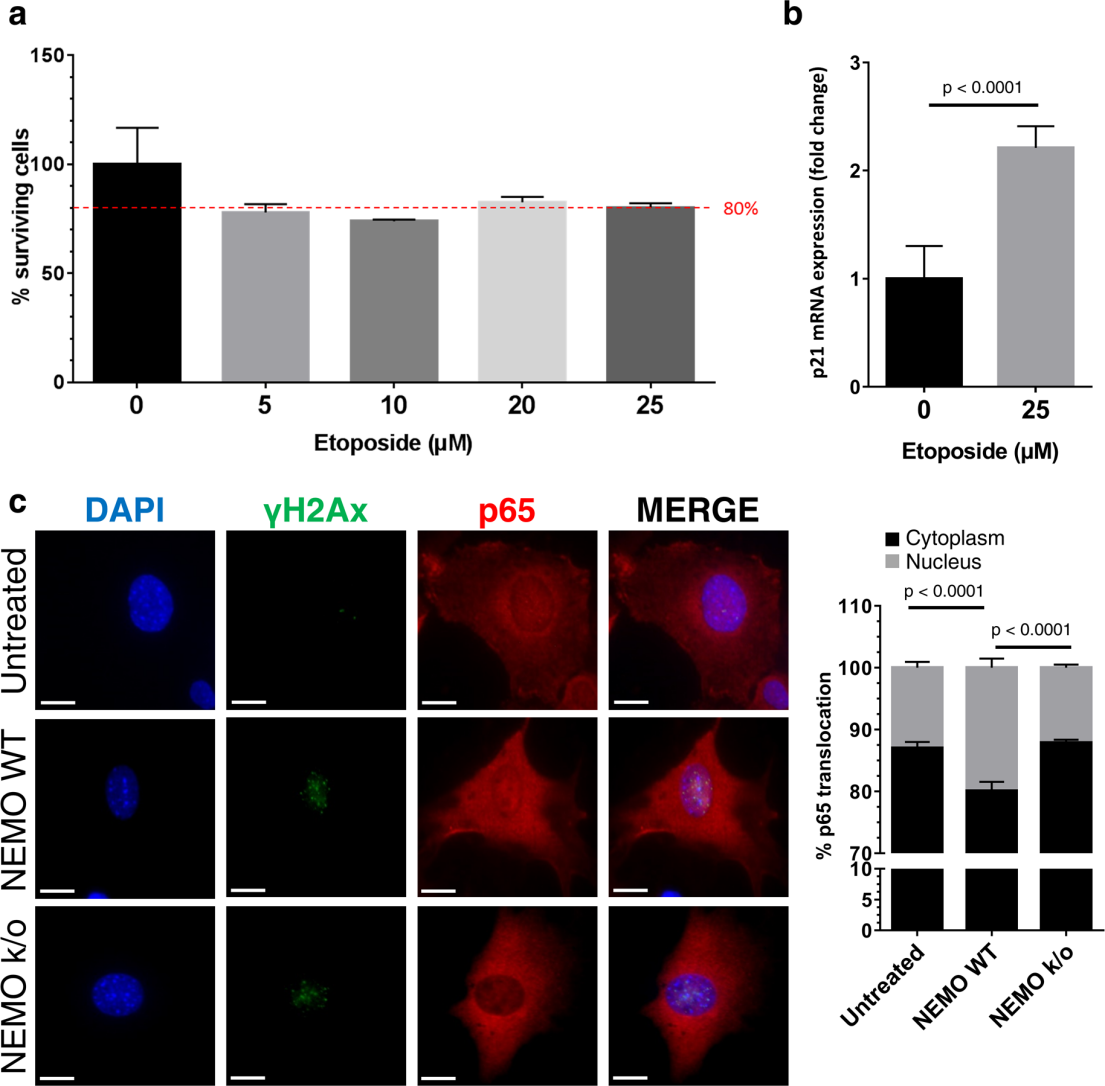
NEMO knockout murine dermal fibroblasts show a decreased nuclear translocation of p65. **a.** MTT assay determined optimal experimental conditions. 80% viable cells was set as threshold. After overnight serum starvation MDFs were treated with etoposide for 3 h followed by a 24 h incubation period. MTT assay was started afterwards to determine the viability of cells. Values are presented as mean ± SEM in percent. (*n* = 3) **b.** In order to evaluate DNA damage response and cell cycle arrest mRNA expression of p21 was analysed by RT-qPCR in MDFs treated with 25μM etoposide for 3 h followed by a 24 h incubation time (*n* = 5). Values are presented as mean ± SEM of fold change. Comparison was made with two-tailed t-test; *P*-value indicated the significance of difference. **c.** Representative immunostaining of γH2Ax (green) and p65 (red) in wildtype (NEMO WT) and NEMO knockout (NEMO k/o) MDFs treated with 25μM etoposide for 3 h with a following incubation period of 24 h. Scale bars, 50μM. The graph shows the percentage of p65 in the cytoplasm (black bars) compared to the nucleus (grey bars) as percentage of red pixels. Values are mean ± SEM in percent. Comparison was made with two-tailed t-test (*n* = 10); line and *P*-value.

In order to evaluate proposed knockouts NEMO was depleted from murine dermal fibroblasts (MDFs) using a NEMO-floxed mouse line. These MDFs were isolated from murine skin and subsequently transfected with a Cre-recombinase coding plasmid including a fluorescence reporter construct (Fig 7). To purify NEMO knockout MDFs, these cells were FACS sorted two days post-transfection (S1A Fig). Successful NEMO knockout was assessed by PCR (S1B Fig) and western blot (S1C Fig). To study the effect of DNA damage, overnight-starved MDFs were treated with 25 μM etoposide, an established DNA damage and senescence inducer, for 3 h followed by a 24 h incubation period [37]. Afterwards cell media supernatant was taken and total RNA was isolated. We first measured p21 mRNA expression as an indicator for DNA damage and cell cycle arrest. Without a significant reduction of cell viability (Fig 8A), p21 mRNA expression was upregulated more than twofold in etoposide treated compared to untreated MDFs (Fig 8B). NEMO is of high importance for DNA damage mediated nuclear translocation of the NF-κB signaling molecule p65. As shown by immunofluorescence staining of untreated NEMO wildtype MDFs compared to etoposide treated wildtype and knockout MDFs, the translocation of p65 into the nucleus upon DNA damage is significantly increased in wildtype whereas it is brought down to the level of untreated wildtype MDFs when NEMO is knocked out (Fig 8C).

### NEMO mediates DNA damage induced expression and secretion of IL-6 and IL-8

**Fig 9:**
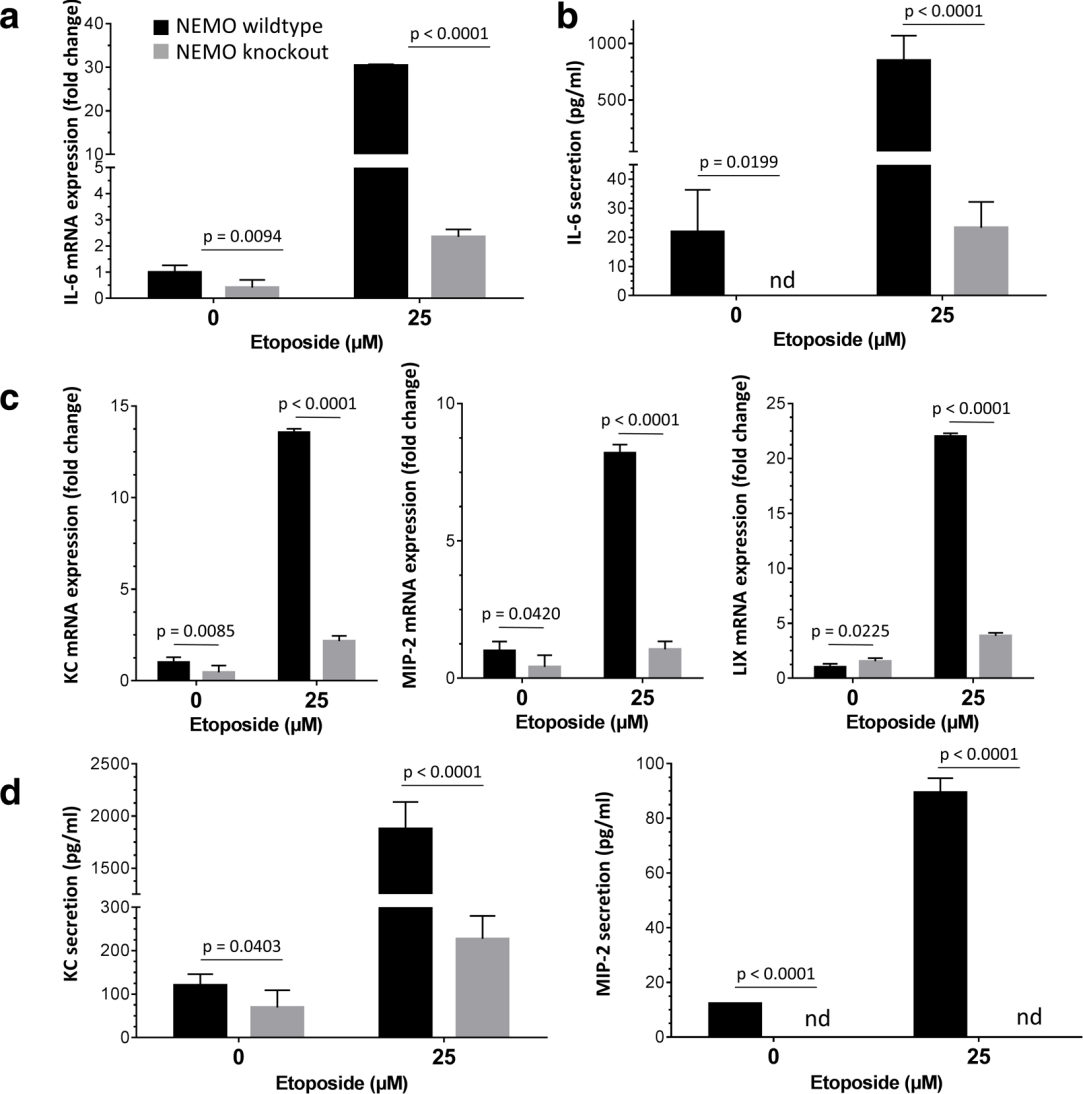
DNA damaged NEMO knockout MDFs show a decrease in IL-6 and IL-8 mRNA expression and protein secretion. **a**. To assess the influence of the NEMO knockout on DNA damage mediated activation of SASP signaling IL-6 mRNA expression was measured by RT-qPCR in untreated and etoposide-treated MDFs (*n* = 5). Cells with wildtype NEMO (black bars) or NEMO knockout (grey bars) were used. Values were presented as mean ± SEM of fold change. Comparison was made with the two-tailed t-test. **b**. IL-6 secretion was measured by ELISA in conditioned media of untreated and etoposide-treated MDFs (*n* = 5). Cells with wildtype NEMO (black bars) or NEMO knockout (grey bars) were used. Values were presented as mean ± SEM of total secretion in pg/ml, nd means non-detectable. Comparison was made with the two-tailed t-test. **c**. In addition to IL-6 murine IL-8 homologues KC, LIX and MIP-2 were used to further show activation of SASP signaling. mRNA of all three homologues was measured by RT-qPCR in untreated and etoposide-treated MDFs (n = 5). Cells with wildtype NEMO (black bars) or NEMO knockout (grey bars) were used. Values were presented as mean ± SEM of fold change. Comparison was made with the two-tailed t-test. **d**. IL-8 homologue secretion was measured by ELISA in conditioned media as previously described (n = 5). Values were presented as mean ± SEM of total secretion in pg/ml, nd means non-detectable. Comparison was made with the two-tailed t-test.

As we have observed the effect of a NEMO knockout on the nuclear translocation of p65 and thereby activation of NF-κB, we further explored the possible suppressive effect on IL-6 and IL-8 activation. To achieve this we isolated total RNA and analyzed the mRNA expression of IL-6 and the murine homologues of IL-8 CXCL1 (KC), CXCL2 (MIP-2) and CXCL5 (LIX). Upon DNA damage, we observed a significant reduction in IL-6 mRNA expression with a strong downregulation in untreated knockout compared to untreated wildtype. An even stronger downregulation in etoposide treated NEMO knockout compared to wildtype MDFs was detected. Taken together a NEMO knockout could reduce DNA-damage mediated IL-6 mRNA expression by almost tenfold (Fig 9A). Next, we measured the secretion of IL-6. While there is nearly no secretion of IL-6 in untreated wildtype as well as knockout MDFs, a strong increase in IL-6 secretion occurred in etoposide treated wildtype MDFs, whereas the NEMO knockout MDFs only shows a small increase in secretion with a more than hundredfold reduction when compared with etoposide treated wildtype cells (Fig 9B). We additionally analyzed the mRNA expression of three murine IL-8 homologues to assess the impact of a NEMO knockout on DNA damage mediated IL-8 expression. We found that all three chosen homologues were significantly downregulated in NEMO knockout MDFs compared to wildtype MDFs after DNA damage. The total expression of IL-8 homologues mRNA in NEMO knockout MDFs was reduced by at least fivefold when compared to treated wildtype MDFs (Fig 9C). There is detectable secretion of IL-8 homologues in untreated wildtype and NEMO knockout MDFs, however the secretion strongly rose upon etoposide treated in wildtype cells whereas there is no detectable increase in the NEMO knockout MDFs. This effect was similarly found for the studied IL-8 homologues KC and MIP-2 (Fig 9D). However, we did not find any significant alteration in the expression of two housekeeping genes, such as beta-actin and 18s rRNA in the NEMO knockout MDFs, compared with NEMO wildtype (S2A Fig). In addition, we also did not observe any significant alteration in the expression of a wide array of genes that were predicted by Boolean network not to be changed after NEMO knockout (S2B Fig). These data show the importance of NEMO and NF-κB signaling for the activation of IL-6 and IL-8 in the case of DNA damage. In early stages DNA damaged and cell cycle arrested MDFs most likely activate secretory SASP signaling through NF-κB rather than other stress pathways.

## Discussion

In the model of DNA damage and proinflammatory signaling presented here we collected and combined previously published knowledge on major regulators of the SASP. Using this model, we identified attractors fitting cell cycle progression and cell cycle arrest as they physiologically occur. This suggests reliability of this model in terms of reproducibility of current biological knowledge. The network model allows us to time- and cost-effectively generate hypotheses and predict gene knockouts that may influence the outcome of the SASP *in-vitro*.

In the process of modeling, we first created individual models of DNA damage and proinflammatory signaling. In a next step, we fused these two sub-networks to the model presented here. In S1 Text, we analyzed the impact of integrating both pathways in one Boolean network model. Our results indicate that there is not only an effect of DNA damage in the proinflammatory signaling but also vice versa. On one hand, we deduce a stabilization of the DNA damage response network as the integration of both sub-networks leads to a reduction of possible attractors (87 to 19). On the other hand, the inner dynamics of each sub-network stay intact, showing biologically reproducible signaling cascades (e.g. Fig 4).

In the simulation without DNA damage, only activation of cell cycle regulation genes that facilitate cell cycle progression were observed [38]. In contrast, when we entered DNA damage into the network, we detected early activation of the DNA damage response (DDR) followed by a p53/p21 mediated cell cycle arrest and at a later time point the activation of proinflammatory signaling through NF-κB [39, 40]. We utilized the Boolean network to simulate knockout and overexpression states that have the power to inhibit both IL-6 and IL-8 activation, such as knockouts of ATM and RelA or the overexpression of IκBα, that have previously been published to decrease IL-8 or IL-6 expression and secretion *in-vitro* [7, 9, 34]. One of the most prominent knockout suggestions obtained was that of NEMO, which acts as an essential modulator of NF-κB signaling and is a major link between DDR and NF-κB signaling [41]. Therefore, it is a suitable target to prevent NF-κB activation, while maintaining the repair potential of the DDR. Taken together these *in-silico* data suggest NF-κB to be one of the major SASP activators in response to DNA damage activating all three mediators of proinflammatory signaling depicted in this network.

For the sake of manageability, the model presented here was limited to a core set of pathways involved in senescence and the SASP. Of course, the value of the results could still be enriched by adding even more components and additional pathways, such as a more detail view on CEBP-signaling, growth factor signaling and the expansion of cell cycle related signaling. This would enable to simulate an even deeper level of signaling involved in the SASP. Another factor that was not viewed in this work is the influence of the intensity levels and timing of expression and stimuli on the outcome of the SASP. Physiologically occurring DNA damage, for example, is not an all or nothing event but rather comes in different levels and lengths of damage that can trigger a multitude of different reactions in the cell. In future works, it would be interesting to add these into the model. Such extension would allow simulations of the exact amount and timing of damage needed to trigger full-blown SASP rather than senescence. Furthermore, it would possibly reveal at which point the cell decides that it is beneficial to trigger SASP signaling in order to warn the system of the damage and initiate clearance as opposed to trying to repair itself.

IL-6 and IL-8 reinforce senescence in an autocrine and paracrine way, concomitantly preventing senescent cells from exiting cell cycle arrest and forcing neighboring cells into senescence themselves [3, 42]. Persistent DDR activity, that is also known to induce IL-6 and IL-8 secretion [9], could be shown in various premalignant and malignant lesions *in-vivo*, and is hypothesized to be one the main causes of aging [9, 43, 44]. Due to this ability to promote invasiveness of cancer cells and the spreading of senescence to neighboring cells IL-6 and IL-8 are of special interest [3, 45]. While it is probably not detrimental to transiently activate the respective signaling pathways, the long-term persistence of unrepairable DNA damage leads to a lasting activation of NF-κB through the DDR mechanisms and thereby to a prolonged stimulation of IL-6 and IL-8. Ultimately, this initiates and perpetuates a vicious cycle from which cells cannot escape and causes the development of the SASP.

To explore and validate previously generated *in-silico* results *in-vitro*, we isolated murine dermal fibroblasts from NEMO-floxed mice and transfected these with a Cre-recombinase plasmid to deplete NEMO. Contrary to NEMO knockout MDFs we observed RelA enrichment in the nucleus in DNA damaged wildtype cells. This suggests that mainly NEMO is responsible for the forwarding of DNA damage signals from the DDR to NF-κB signaling.

We were particularly interested in achieving inhibition of IL-6 and IL-8 expression and secretion *in-silico* and *in-vitro*. As we could show in our in-vitro results, DNA damaged NEMO knockout cells did not reveal any induction of IL-6 or IL-8 homologue mRNA expression, suggesting that DNA damage-triggered IL-6 and IL-8 expression is mainly conferred by NF-κB signaling. This was confirmed on protein level, showing a strong decrease in secretion of both IL-6 and IL-8 homologues in NEMO knockout MDFs. In conclusion, abolishing NEMO is sufficient to not only block the signaling from DDR to NF-κB but also to decrease expression and secretion of two of the most prominent and established SASP mediators IL-6 and IL-8.

The question arises why damaged senescent cells have to start expressing and secreting factors that are detrimental to themselves, surrounding cells and tissues. The secretion of many SASP factors can be explained firstly by the attempt to clear senescent cells from tissue by cells of the innate immune system and secondly as a warning to the microenvironment that there is a danger in the near vicinity. Senescent cells secrete different factors that attract phagocytic immune cells and induce proteolytic enzymes to facilitate their migration through the extracellular matrix [46]. As long as damaged cells can be cleared in early phases the SASP is probably beneficial for the organism, however once the immune system cannot keep up with the emergence of damaged cells, detrimental effects accumulate and tissue takes damage [2, 47]. In this phase, it would be beneficial to have the possibility to counteract the SASP and give the immune system time to catch up.

In summary, we could illustrate that *in-silico* identification of genes with mechanistic contribution in the regulation of the SASP, confirmed under experimental conditions *in-vitro*, is a highly suitable approach and holds substantial promise to identifying therapeutic targets to delay or even prevent the detrimental SASP effects on tissue homeostasis and overall ageing. Using our Boolean model, we were able to reproduce published data *in-silico* and generate various knockout proposals to shut down two of the most detrimental effectors of the SASP. This is of major clinical relevance in terms of tissue aging. In fact, SASP factors like IL-6 and IL-8 have been correlated with inflammaging not only driving the aging process itself, but also promoting aging associated morbidity, frailty and mortality [48]. We additionally were able to validate and prove one of the most prominent knockout suggestions *in-vitro*, keeping in mind that there might always be detrimental off-target effects when altering a major signaling pathway like NF-κB. However, targeting NEMO and its interaction partners, as already shown in studies of inflammatory arthritis and diffuse large B-cell lymphoma, may hold promise for the development of new therapies for age-related pathologies in which senescence and the SASP play a role [49, 50].

## Methods

### Mice experiments

Murine dermal fibroblasts from an inducible connective tissue-specific NEMO-deficient mouse model were used for *in-vitro* experiments. This mouse line (Col(I)α2-CreERT^+^;NEMO^f/f^) was generated by crossing Col(I)α2-CreERT transgenic mice [51] with NEMO floxed mice [35]. These mice were backcrossed to C57BL/6J for at least 6 generations. They were maintained in the Animal Facility of the University of Ulm with 12 h light–dark cycle and SPF conditions. The breeding of the mice and all experiments were approved by the animal ethical committee (approval number, Tierversuch-Nr. 1102, Regierungspräsidium Tübingen, Germany). For mice genotyping standard PCR techniques were used. The sequences of the primers used in this manuscript are summarized in S1 Table. Briefly, DNA was isolated from the tail tip of an individual mouse using a commercial kit (Easy DNA kit, Invitrogen). Purified DNA was later dissolved in TE and used for PCR amplification. The PCR products were run in QIAxcel Advance system (Qiagen) using the program AM320 and then documented digitally.

### Isolation and culture of murine dermal fibroblasts

Murine dermal fibroblasts (MDFs) were isolated from ear skin of young mice and cultured as previously described [52].

### Induction of DNA damage

DNA damage was induced by adding etoposide to cell culture media at a concentration of 25 μM for 3 hours after overnight serum-starvation. Supernatants subsequently removed and cells were rinsed with PBS before adding fresh culture media. Cells and/or media were used 24 h later for further analysis.

### Cloning

Recombineering technology [53] was used to constract plasmids containing CDS of both Cre recombinase and fluorescence reporter, mRuby2 or only mRuby2. pCAG-Cre vector (a gift from Connie Cepko, Addgene plasmid # 13775) was used for the recombineering. In the first construct, the aim was to insert the T2A-mRuby2 sequence before the stop codon of Cre recombinase and in the second construct, the aim was to replace the Cre ORF with mRuby2 ORF. In brief, synthetic DNA fragments were synthesized either as gBlock (IDT) or as GeneArt string (Thermo Scientific). Four DNA fragments were synthesized, the first one contained 5’ 50 bp homology regions to the vector (targeting 50 nucleotide upstream of Cre ORF stop codon), chloramphenicol and ccdB cassettes and 3’ terminal 50 bp homology regions to the vector (targeting 50 nucleotide downstream of last amino acid coding codon of Cre ORF, i.e., condon preceding the Cre ORF stop codon). The second synthetic fragment contained 5’ 50 bp homology regions to the vector (targeting 50 nucleotide upstream of Cre ORF start codon), chloramphenicol and ccdB cassettes and 3’ terminal 50 bp homology regions to the vector (targeting 50 nucleotide downsteram of Cre ORF stop codon). The third synthetic fragment contained 5’ 50 bp homology regions to the vector (same as fragment 1), T2A sequence-mRuby2 ORF and 3’ terminal 50 bp homology regions to the vector (same as fragment 1). The fourth synthetic fragment contained 5’ 50 bp homology regions to the vector (same as fragment 2), mRuby2 ORF and 3’ terminal 50 bp homology regions to the vector (same as fragment 2). E. coli containing pCAG-Cre was processed for electrocompetent using standard methods and these electrocompetent E coli, containing pCAG-Cre were electroporated with a dual inducible expression plasmid pSC101-ccdA-gbaA (a gift from Prof. A. Francis Stewart) and selected for ampicillin 100μg/ml and tetracycline 3.5μg/ml at 30°C. Next day, 4–5 colonies were expanded and the expression of recombineering proteins, λphage *redα*, *redβ and redγ* and *rec*A (*red*gbaA) was induced by L-rhamnose (1.4mg/ml). After 1 h of L-rhamnose treatment, the induced E. coli were processed for electrocompetent and then electroprorated either with synthetic DNA fragments 1 or 2. After 1 h of recovery in SOC medium, the electroporated E coli, were plated in LB-agar containing ampicillin 100μg/ml, tetracycline 3.5μg/ml, chloramphenicol 25μg/ml and 1.4mg/ml L-arabinose. L-arabinose addition induced the expression of ccdA, the antidote of ccdB in that only recombined plasmid containing E. coli can survive. Thereafter colonies from fragment 1 and fragment 2 electroporated E. coli plates were picked and expanded for the verification of first recombinant product using restriction digestion analyses. The corresponding colony was expanded and *red*gbaA expression was induced by L-rhamnose for 1 h. The induced E. coli containing either recombined DNA fragment 1 or fragment 2 were made electrocompetent for the second round of recombineering. The E. coli, containing recombined DNA fragment 1 then electroporated with synthetic DNA fragment 3. The E. coli, containing recombined DNA fragment 2 were electroporated with synthetic DNA fragment 4. The recovered electroporated E. coli were plated in LB-agar containing ampicillin 100μg/ml and incubated at 37°C overnight. Colonies from both plates were picked, expanded and verified for the second recombinant products. The correct plasmids were sequenced and verified through commercial services (Sequiserve, Germany). Plasmid preparation was performed using a commercially available kit (Qiagen plasmid plus kit, Qiagen). This plasmid (pCAG-Cre-T2A-mRuby2) can be obtained from the authors on request and was deposited in the Addgene repository (Accession ID 102989).

### Initiation of Cre activity (NEMO knockout)

Early passage MDFs with a floxed NEMO allele were transfected with a Cre expressing vector using an electroporation-based transfection method (Amaxa, Lonza Group). Transfer of the plasmid was performed using a commercial kit with the AMAXA program N24 (Nucleofector Kits for Mouse or Rat Hepatocytes, Lonza). Successful NEMO knockout was assessed by PCR as explained before.

### FACS sorting of positive cells

Two days after transfection cell populations were purified using the mRuby2-based reporter system included in the previously described Cre-expressing vectors. Gating was set for living cells and singlets, sorting was based on mRuby2 expression in the PE-channel. FACS-sorting was performed with a FACSAria III system (BD Biosciences) and analysis was done on FACSDiva and FlowJo (Tree Star) software.

### Immunofluorescence staining

Cells were fixed in 4% PFA in PBS for 15 min and thereafter treated with 0.1% Triton X-100 for 10 min at room temperature. Blocking was performed in 5% BSA for 1 h at room temperature. Anti-p65 (#8242, 1:200, Cell Signaling) and anti-γH2A.x (ab22551, 1:200, Abcam) were used as primary antibodies overnight at 4°C. Incubation with the secondary antibody Alexa 488 goat anti-mouse (for γH2A.x, 1:500) and Alexa 555 goat anti-rabbit (for p65, 1:500) was performed at room temperature for 1 h.

### Western blotting

Western blot analyses were performed as described earlier [54]. In brief, murine dermal fibroblasts were lysed in RIPA lysis buffer (25mM Tris-HCl pH 7.6, 150mM NaCl, 1% NP-40, 1% sodium deoxycholate, 0.1% SDS) supplemented with protease and phosphatase inhibitors (Thermo Scientific). Cells in RIPA were sonicated using sonopuls HD 2070 and MS72 microtips (Bandelin). The sonicator setting was 50% power 3 cycles and 10 sec for three times. Following sonication, the lysate was centrifuged for 15 min at 14000 rpm and 4°C. The supernatant was collected and protein concentration was measured by Bradford Assay (Biorad). 50μg of protein from each lysate was resolved in 4–20% SDS-PAGE, followed by transfer to nitrocellulose membrane and probing the membrane with anti-NEMO antibody (1:1000, Abcam). The membrane was incubated with goat anti-rabbit IgG coupled with HRP for 1 hr (Jackson ImmunoResearch). Thereafter the membrane was developed by LumiGLO chemiluminescence reagent (Cell Signaling Technologies) using Fusion FX7 Geldoc system (Vilber Lourmat), followed by stripping with Restore Plus Western blot Stripping Buffer (Thermo Scientific) and re-probed with anti-β-actin antibody coupled with HRP (1:12000, Santa Cruz), finally developed the membrane using LumiGLO.

### Quantitative PCR

Twenty-four hours after treatment, total RNA was isolated from cultured murine dermal fibroblasts using a commercial kit (RNeasy Mini Kit, Qiagen) as described by the manufacturer. Two μg of RNA per sample were reverse transcribed using illustra Ready-To-Go RT-PCR Beads (GE Healthcare). Quantity and quality of total RNA and cDNA was assessed using Nanodrop 1000 (Thermo Scientific) and QIAxcel Advance system (Qiagen). The 7300 real time PCR system (Applied Biosystem, Life Technologies) was used to amplify cDNA using Power SYBR green mastermix (Applied Biosystems, Life Technologies). Sequences for primers used in all experiments and genotyping are provided in S1 Table.

### ELISA

After etoposide treatment cells were supplied with fresh culture media. Culture media was taken for analysis of secreted IL-6 and murine IL-8 homologues (KC and MIP-2) 24 h after treatment. Media was stored at -80°C until analysis.

Concentrations of secreted IL-6 and murine IL-8 homologues after DNA damage were determined using commercial kits (Mouse IL-6/KC/MIP-2 Quantikine ELISA Kit, R&D) as described by the manufacturer.

### Statistical calculations

The influence of a NEMO knockout was compared to wildtype controls based on IL-6, IL-8 homologue and p21 mRNA expression as well as IL-6 and IL-8 homologue protein secretion. The sample size for all experiments was 5 per group. The expression and secretion of the two groups was tested using unpaired two-tailed t-test. Furthermore, the influence of the NEMO knockout compared to wildtype controls on the nuclear translocation of p65 was measured by the percentage of fluorescence intensity in the cell nucleus as well as cytoplasm (sample size = 10). The fluorescence intensity was tested using unpaired two-tailed t-test. The exact p-values are depicted in the respective figures. The figures show mean values. Error bars correspond to the standard error of the mean.

### Boolean networks

In a first step, IL- and DNA-damage pathways included in the Boolean model of SASP were reconstructed individually. To generate the independent gene regulatory networks of inflammatory and DDR signaling, we collected peer-reviewed literature that is considered relevant in the context of SASP (see Table 1). This literature reports data about the local interaction of key genes regulating each pathway. The information was collected in murine and human experimental *in-vivo* and *in-vitro* studies. In order to control the complexity of model we restricted the set of regulatory factors in the model to the most relevant for SASP and to those being important components of each pathway. The modeled pathways were chosen based on the requirement in the onset and maintenance of the SASP shown in studies related to senescence and the SASP. In total 80 publications were used to determine the relationships between the individual components of the model (Table 1).

After the reconstruction of Boolean network models of inflammation and DNA damage response, both were combined into a larger network. The impact of combining the two network models instead of simulating them independently is shown by additional analysis in S1 Text. Simulations based on specific environmental (input) conditions were performed to find the corresponding attractors. Furthermore, to identify possible interaction targets, i.e., to generate testable hypotheses about interventions, we fixed corresponding regulatory factors to either 0 or 1 (modelling of knockout or overexpression, similar to [55]) and reran the simulations (S1 Text). Given an interaction target, we looked for the attractors that positively influence the DNA damage response phenotype.

Network figures were drawn with Biotapestry (www.biotapestry.org). Simulations of the Boolean network were performed with the package BoolNet [12, 56] in R (www.r-project.org).

This model contains two external signals (DNA damage and Activated Oncogenes). These signals do not coincide with genes within the network, but represent different stimuli from external or internal sources that are known to activate the DNA damage response and/or cell cycle arrest signaling through either p16^INK4^ or p53/p21.

## Supporting information

S1 Fig Establishment of a pure NEMO knockout murine dermal fibroblast (MDF) population. **a.** To purify NEMO k/o MDFs, NEMO-floxed cells were transfected with a Cre-recombinase vector including a mRUBY2-reporter construct. Two days post-transfection cells were purified for the NEMO k/o using flowcytometry-based sorting, gating for living cells, cell singlets and mRUBY2 signal (histograms; left to right). **b.** Successful NEMO k/o was determined using PCR analysis. DNA was isolated from FACS-sorted MDFs and later used for PCR amplification. Cre-recombinase activity induced the deletion of floxed NEMO alleles resulting in a bigger sized amplification product in successful knockouts as compared to wildtype cells. **c.** In addition to PCR analysis a successful knockout on protein level was determined by western blotting of cell lysates equilibrated to actin expression levels.
(TIF)

S2 Fig Unaltered expression of selected genes (predicted to be unaffected in NEMO knockout) following NEMO knockout. The expression level of a set of genes that were predicted not to be changed after NEMO knockout by the Boolean network model. In a setting of 2-fold cutoff (blue dotted line), the expression of all genes remained unaltered between control and NEMO knock out MDFs. Dotted line at value ‘1’ represents level of expression in the control MDFs.
(TIF)

S1 Table Primer sequences. (DOCX)

S1 Text Simulation of SASP network with BoolNet. (PDF)
