# Supplementary figures and images for: A model of the onset of the senescence associated secretory phenotype after DNA damage induced senescence
Source: PLoS Comput Biol. 2017 Dec 4;13(12):e1005741. doi: 10.1371/journal.pcbi.1005741 (PMC5730191; doi:10.1371/journal.pcbi.1005741)

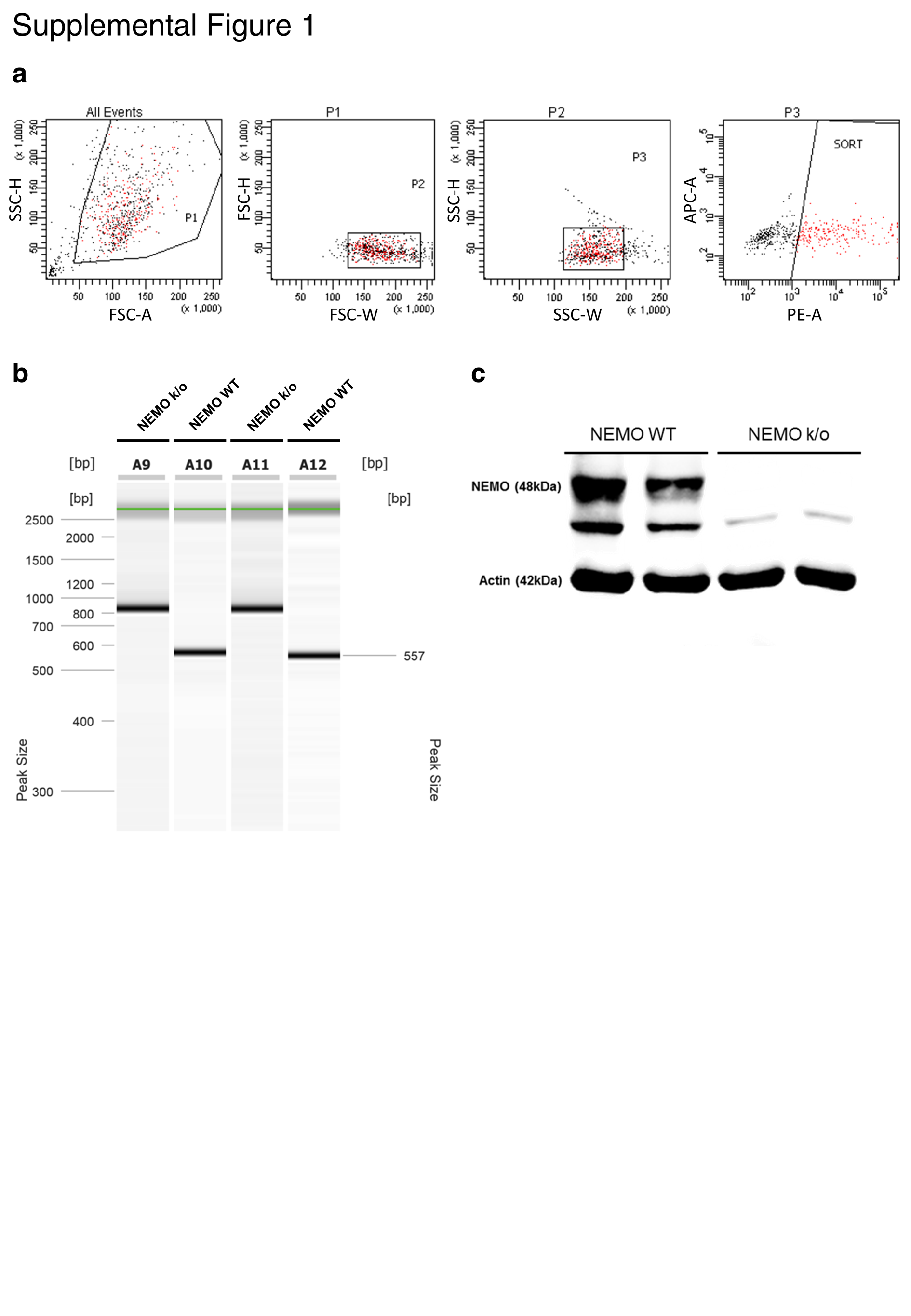

Supplement: S1 Fig — a. To purify NEMO k/o MDFs, NEMO-floxed cells were transfected with a Cre-recombinase vector including a mRUBY2-reporter construct. Two days post-transfection cells were purified for the NEMO k/o using flowcytometry-based sorting, gating for living cells, cell singlets and mRUBY2 signal (histograms; left to right). b. Successful NEMO k/o was determined using PCR analysis. DNA was isolated from FACS-sorted MDFs and later used for PCR amplification. Cre-recombinase activity induced the deletion of floxed NEMO alleles resulting in a bigger sized amplification product in successful knockouts as compared to wildtype cells. c. In addition to PCR analysis a successful knockout on protein level was determined by western blotting of cell lysates equilibrated to actin expression levels. (TIF) [file pcbi.1005741.s001.tif]

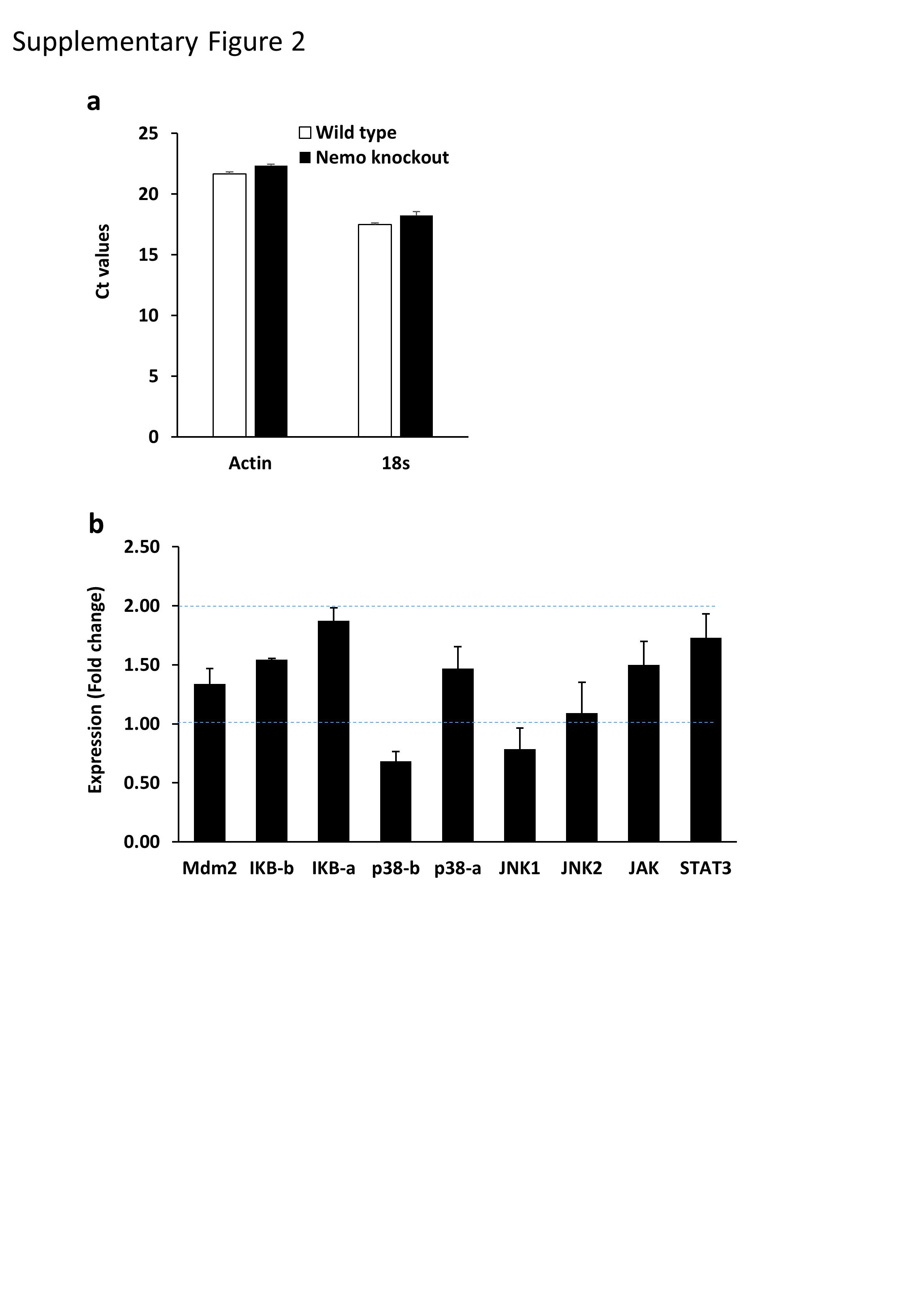

Supplement: S2 Fig — The expression level of a set of genes that were predicted not to be changed after NEMO knockout by the Boolean network model. In a setting of 2-fold cutoff (blue dotted line), the expression of all genes remained unaltered between control and NEMO knock out MDFs. Dotted line at value ‘1’ represents level of expression in the control MDFs. (TIF) [file pcbi.1005741.s002.tif]
