# Supplementary material for: A model of the onset of the senescence associated secretory phenotype after DNA damage induced senescence
Source: PLoS Comput Biol. 2017 Dec 4;13(12):e1005741. doi: 10.1371/journal.pcbi.1005741 (PMC5730191; doi:10.1371/journal.pcbi.1005741)
Supplement: S1 Table — (DOCX) [file pcbi.1005741.s003.docx]

# Supplements

Supplementary Table 1: Primer Sequences

| **Gene** | **Primer** | **Sequence** | **Application** |
| --- | --- | --- | --- |
| Nemo | FP | TTCTTGGAAGGGTATGGCCAG | Genotyping |
|  | RP | TCCGGGCTTCCTGGAATTT |  |
|  | DEL-RP | GGCCTCACACAAGCAAAGCAT |  |
| IL-6 | FP | TTCCAGAAACCGCTATGAAG | QPCR |
|  | RP | CAGCATCAGTCCCAAGAAG |  |
| KC | FP | AGAGCTTGAAGGTGTTGC | QPCR |
|  | RP | CATTCTTGAGTGTGGCTATGA |  |
| MIP-2 | FP | GTTTGCCTTGACCCTGAA | QPCR |
|  | RP | TCAGTTAGCCTTGCCTTTG |  |
| LIX | FP | GTTGTTTCTTGGGTGTGTTAAG | QPCR |
|  | RP | CATGACACAGCAGCTTTCTA |  |
| p21 | FP | CTCAGTGACTTCTCCCATTTC | QPCR |
|  | RP | GTCCTGTGAGCTCCCTTA |  |
| beta-Actin | FP | CCTTCTTGGGTATGGAATCCTGTGG | QPCR |
|  | RP | CAGCACTGTGTTGGCATAGAGGTCTTTAC |  |
| Mdm2 | FP | GAGCGCAAAACGACACTTACAC | QPCR |
|  | RP | CTGCTTCTCGTCATATAACCTCTTAGTC |  |
| IKB beta | FP | CATCCGGTGGCACAATCAGG | QPCR |
|  | RP | ATGATCTGGATTTCGAGGCACC |  |
| IKB alpha | FP | GGTTTCGGGAACGTCAGTCTG | QPCR |
|  | RP | AGCTCTAAACGACAAGACTTAATTGC |  |
| p38 MAPK beta | FP | GTACACTTCGCTGAAATCCTCG | QPCR |
|  | RP | AAGCACCTGAAGCACGAGAAC |  |
| p38 MAPK alpha | FP | CTATGGCTCGGTGTGTGCTG | QPCR |
|  | RP | GGCGTGAATGATGGACTGAAAC |  |
| JNK1 | FP | AGGAGCTCAAGGAATAGTGTGTG | QPCR |
|  | RP | CGCTTAGCATGGGTCTGATTCTG |  |
| JNK2 | FP | TGCATTCAGCTGGTATCATTCATAGAG | QPCR |
|  | RP | GCCAGGCCAAAGTCAAGGATC |  |
| JNK3 | FP | CATGGGCTACAAGGAGAACGTG | QPCR |
|  | RP | CACTGGTCAATATAGTCCCTTCCG |  |
| JAK1 | FP | TCCGAACCGAATCATCACTGTG | QPCR |
|  | RP | GTTGTCATTGGTTCCGTGCC |  |
| JAK2 | FP | CCAGGCATGACATACTCTACAGG | QPCR |
|  | RP | ACACTCCGTATCTGTAGGTTCTGC |  |
| STAT3 | FP | TGGCACCTTGGATTGAGAGTC | QPCR |
|  | RP | CCCAAGAGATTATGAAACACCAACG |  |
