# Supplementary material for: A model of the onset of the senescence associated secretory phenotype after DNA damage induced senescence
Source: PLoS Comput Biol. 2017 Dec 4;13(12):e1005741. doi: 10.1371/journal.pcbi.1005741 (PMC5730191; doi:10.1371/journal.pcbi.1005741)
Supplement: S1 Text — (PDF) [file pcbi.1005741.s004.pdf]

Simulation of SASP network with BoolNet

Andre Burkovski, Julian Schwab and Hans A. Kestler

This document is supporting information for the manuscript Meyer et. al. "A model of the onset of the Senescence Associated Secretory Phenotype after DNA damage induced Senescence".

The `SASP` network is stored in *SBML* format in file `sasp-sbml.xml`. The rules are described in Table 1 of the main manuscript. It can be loaded and analyzed by using BoolNet (among others):

```
saspnetwork <- loadSBML("sasp.sbml")

#exhaustively search for attractors using synchronous updates
saspAttrs <- getAttractors(saspnetwork, method = "sat.exhaustive")
```

Subnetwork simulation

The `SASP` network comprises two major sub-processes: the DNA damage response and inflammation pathways. These two pathways are modeled as individual Boolean networks. In the next step both networks were integrated to the complete model of the `SASP`-network. We compared the attractors of the two individual sub-networks with those of the complete `SASP`-network in order to investigate and reveal synergistic effects.

DNA damage sub-network

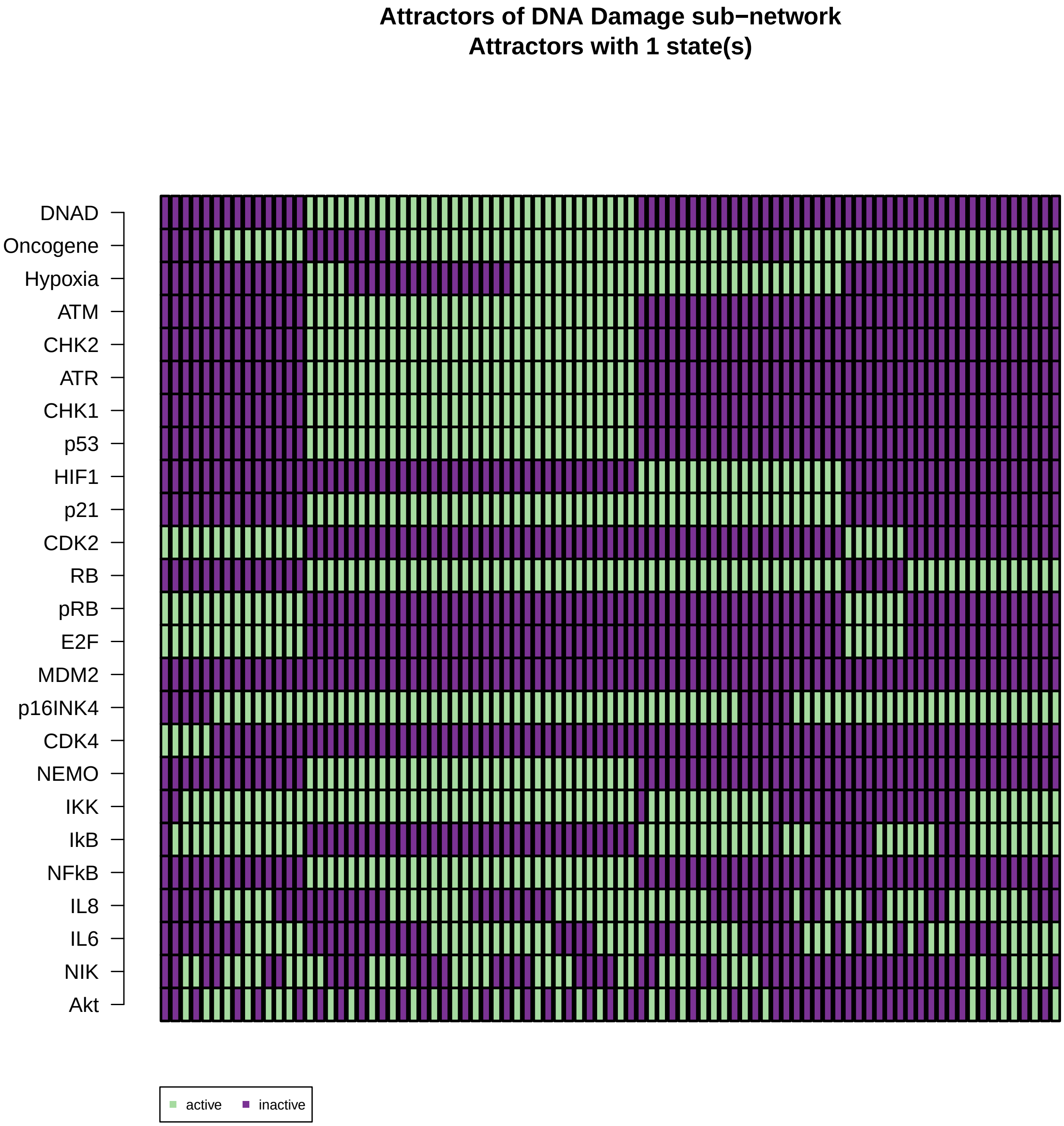


```
Boolean network with 25 genes

Involved genes:
DNAD Oncogene Hypoxia ATM CHK2 ATR CHK1 p53 HIF1 p21 CDK2 RB
pRB E2F MDM2 p16INK4 CDK4 NEMO IKK IkB NFkB IL8 IL6 NIK

Transition functions:
DNAD = DNAD
Oncogene = IL8 | IL6
Hypoxia = Hypoxia
```

```
ATM = DNAD
CHK2 = ATM
ATR = DNAD
CHK1 = ATR
p53 = (CHK2 | CHK1 | ATM) & ! MDM2
HIF1 = Hypoxia & ! p53
p21 = p53 | HIF1
CDK2 = E2F & ! p21
RB = ! (pRB | CDK4 | CDK2)
pRB = (CDK4 | CDK2)
E2F = (pRB | E2F) & ! RB
MDM2 = p53 & ! ATM
p16INK4 = (Oncogene | DNAD)
CDK4 = ! (p16INK4 | p21)
NEMO = ATM
IKK = NEMO | NIK | Akt
IkB = (NFkB | IkB) & !(IKK & NEMO)
NFkB = IKK & ! IkB
IL8 = IL8
IL6 = IL6
NIK = NIK
Akt = Akt
no. dmg attractors : [1] 87
```

Exhaustive attractor search in the DNA damage sub-network leads to 87 different single state attractors. In the following part the patterns in the attractors of the DNA damage network and of the complete `SASP`-network are compared. This is done by excluding the inflammation related regulatory factors from the attractors in the complete network and then comparing the attractors with the DNA damage attractors.

```
par(mar=c(5,7,5,4))
##compare attractors of subparts and complete network
dnaDmgGenes <- dnaDmg$genes #genes relevant for comparsion
#mark regulatory factor from inflammation sub-network as excluded
excludedGenes <- saspnetwork$genes[!(saspnetwork$genes %in% dnaDmgGenes)]
##create attractor matrices
saspAttrMatrices <- lapply(saspAttrs$attractors,
                           getStateMatrix,
                           genes = saspnetwork$genes,
                           excludedGenes = excludedGenes)
##search corresponding vector of dnaDamage attractors in complete model
correspondingAttrsDmg <- lapply(dnaDmgAttrVecs, function(v) {
  matchingAttrs <- lapply(1:length(saspAttrMatrices), function(m) {
    if(attractorExists(saspAttrMatrices[[m]],
                       v,
                       partly = T))
      m
    else
      NULL
})})

#name attractor matchings (outer list -> DNA DMG, inner lists -> complete sasp)
names(correspondingAttrsDmg) <- paste0("DMG Attr ", 1:length(correspondingAttrsDmg))
correspondingAttrsDmg <- lapply(correspondingAttrsDmg, function(a) {
  ret <- a
  names(ret) <- paste0("Complete Attr ", 1:length(a))
```

```
  return(ret)})

#extract idx of identified attractors of completenetwork in subnetwork
foundDmg <- unique(unlist(correspondingAttrsDmg))

print("Matching attractors of DNA damage subpart in complete network : ")
print(sort(foundDmg))

#print matching attractor ids
foundMatches <- gsub("\\.", " -> ", names(unlist(correspondingAttrsDmg)))
invisible(sapply(foundMatches,print))

#select matching attractors of complete network
matchingOrigs <- saspAttrs$attractors[foundDmg]
matchingOrigs <- list(stateInfo = saspAttrs$stateInfo, attractors = matchingOrigs)
class(matchingOrigs) <- "AttractorInfo"
par(mar=c(5,7,5,4))
p <- plotAttractors(matchingOrigs,
                   title = "Attractors of SASP found in DNA Dmg sub-network",
                   allInOnePlot = F,
                   offColor = "#7b3294", onColor = "#a6dba0")
```

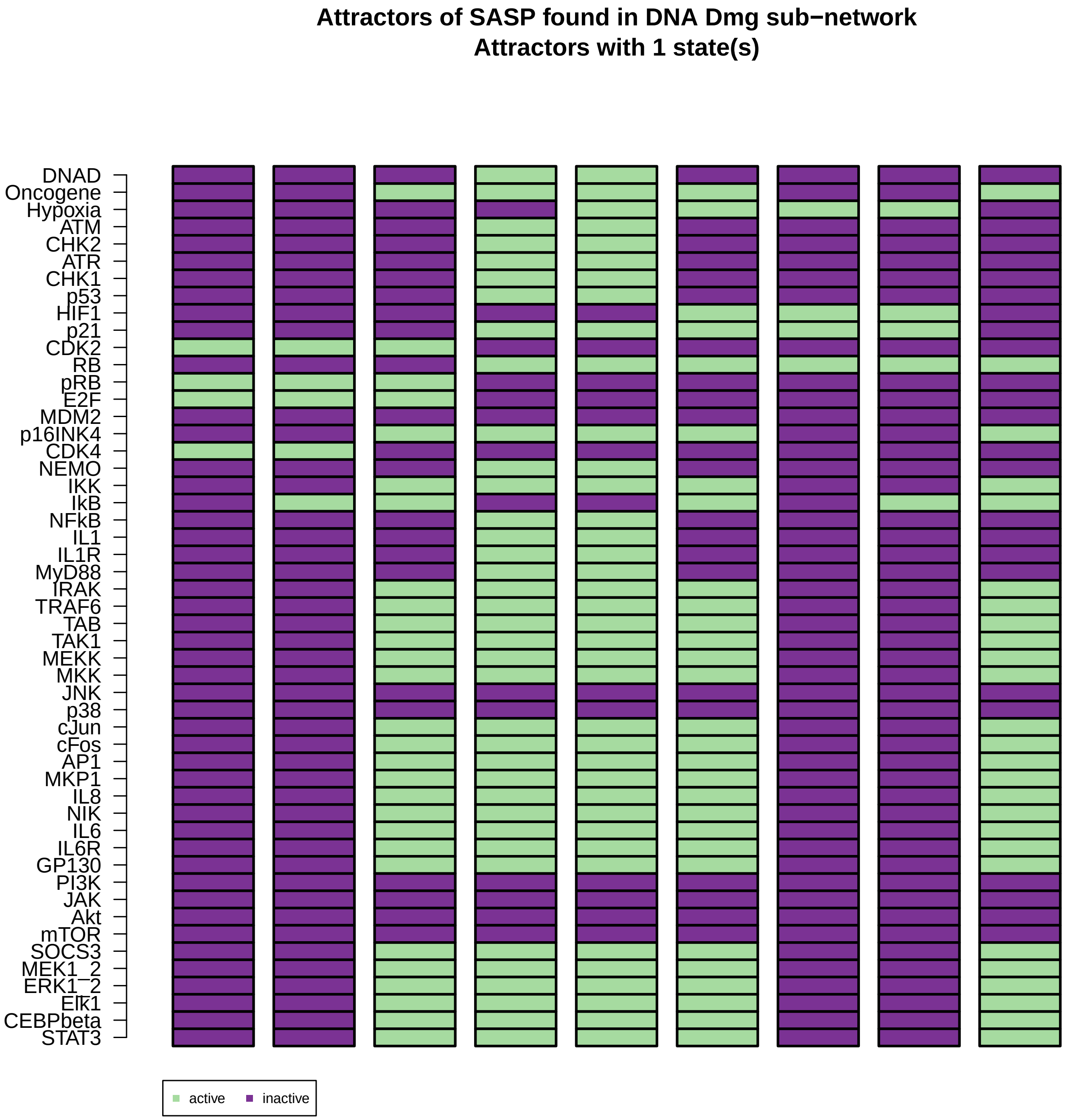


```
[1] "Matching attractors of DNA damage subpart in complete network : "
[1]  1  2  3  4  5  6  7  8 15
[1] "DMG Attr 1 -> Complete Attr 2"
[1] "DMG Attr 2 -> Complete Attr 3"
[1] "DMG Attr 10 -> Complete Attr 7"
[1] "DMG Attr 30 -> Complete Attr 6"
[1] "DMG Attr 46 -> Complete Attr 5"
[1] "DMG Attr 52 -> Complete Attr 8"
[1] "DMG Attr 60 -> Complete Attr 1"
[1] "DMG Attr 61 -> Complete Attr 4"
[1] "DMG Attr 84 -> Complete Attr 15"
```

Inflammation sub-network

```
inflamm <- loadNetwork("inflammationSubpart.txt")
printNetwork(inflamm)
inflammAttrs <- getAttractors(inflamm, method = "sat.exhaustive")
cat("no. inflamm attractors")
length(inflammAttrs$attractors)
inflammAttrVecs <- lapply(inflammAttrs$attractors,
                          getStateMatrix,
                          genes = inflamm$genes,
                          excludedGenes = c())
par(mar=c(5,7,5,4))
p <-plotAttractors(inflammAttrs,
                   title = "Attractors of inflammation subnetwork",
                   allInOnePlot = F,
                   offColor = "#7b3294", onColor = "#a6dba0")
```

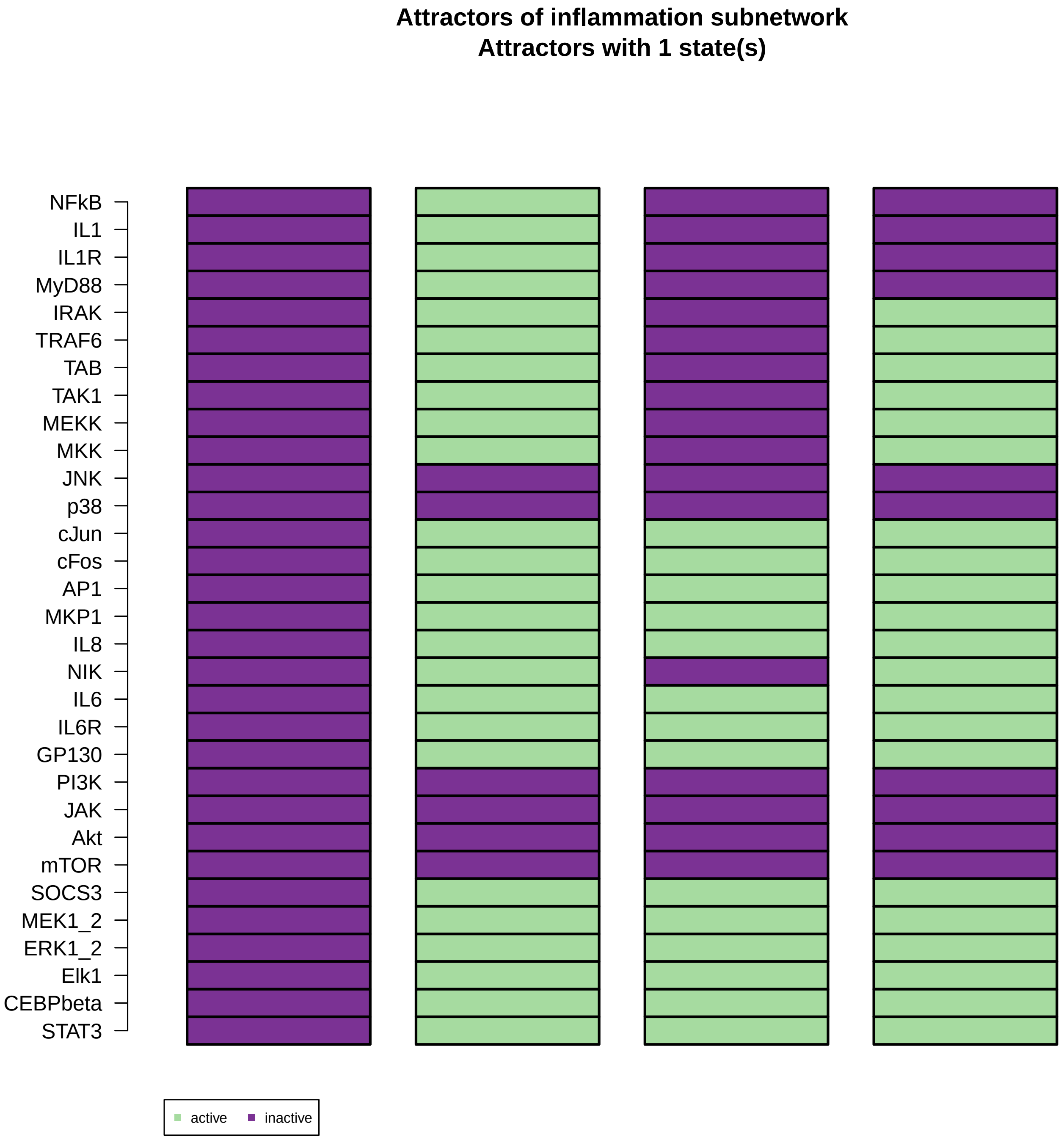
Attractors of inflammation subnetwork
Attractors with 1 state(s)
NFkB
IL1
IL1R
MyD88
IRAK
TRAF6
TAB
TAK1
MEKK
MKK
JNK
p38
cJun
cFos
AP1
MKP1
IL8
NIK
IL6
IL6R
GP130
PI3K
JAK
Akt
mTOR
SOCS3
MEK1_2
ERK1_2
Elk1
CEBPbeta
STAT3
active
inactive

Attractors of inflammation subnetwork
Attractors with 3 state(s)

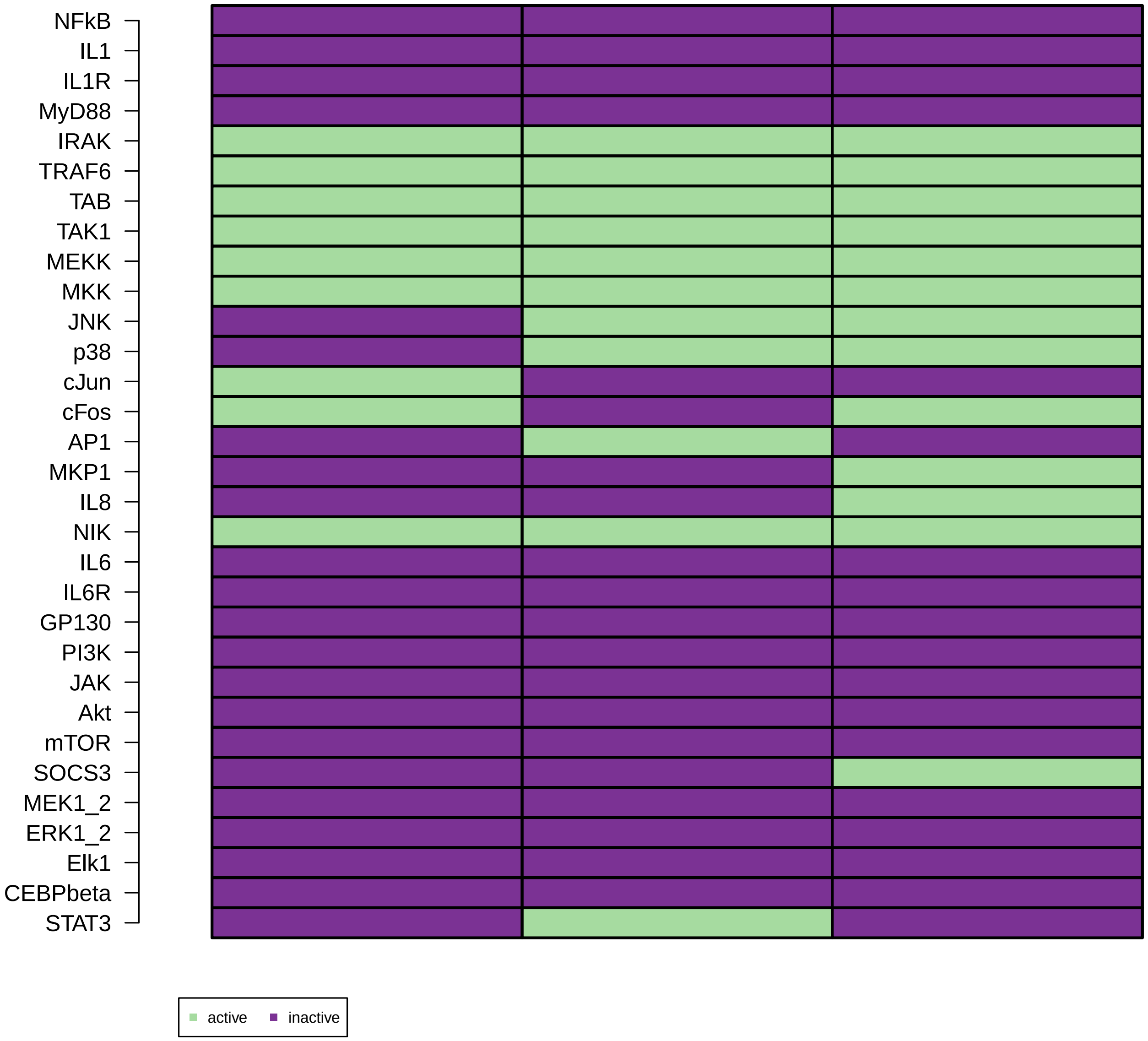

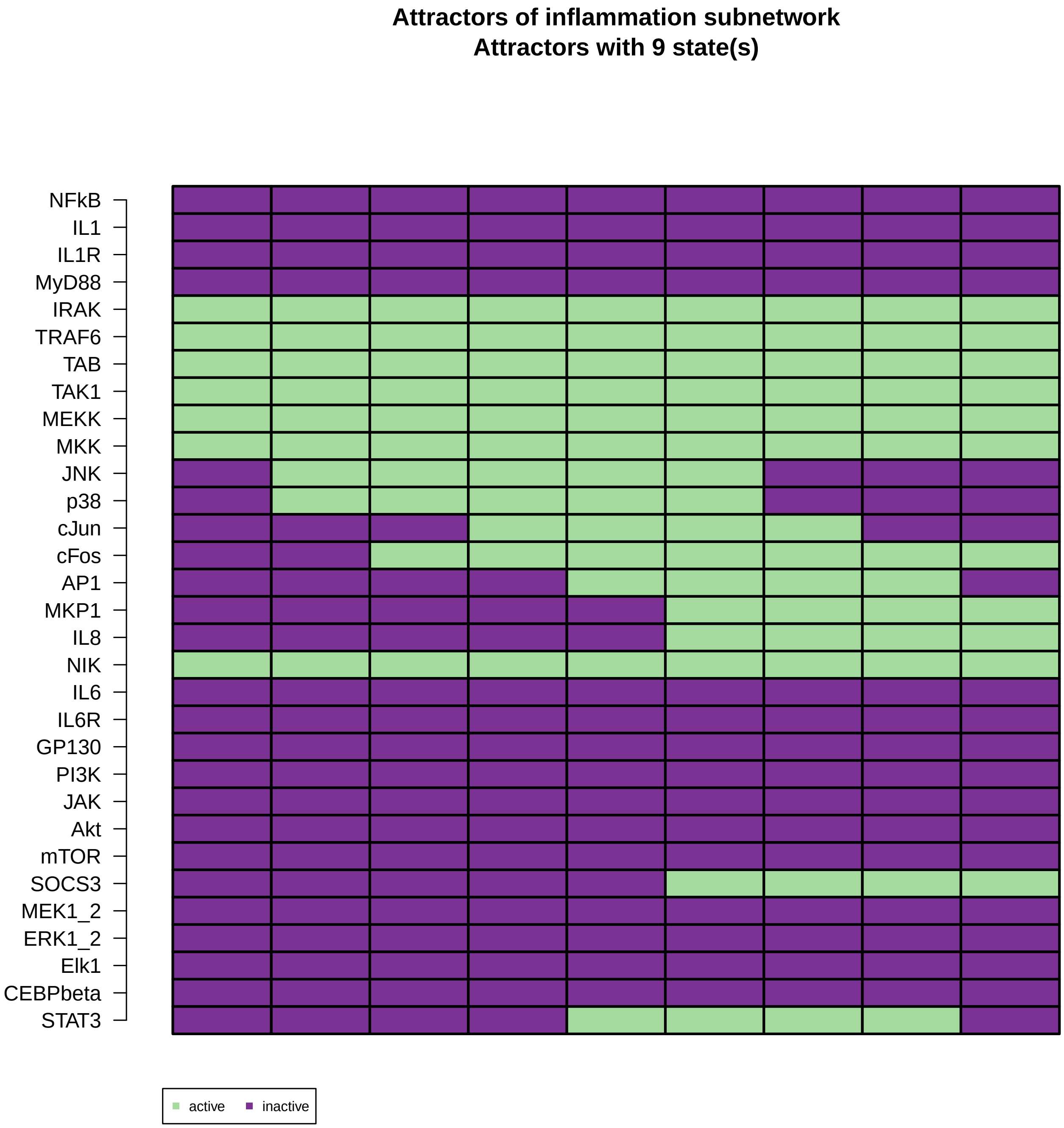


```
Boolean network with 31 genes

Involved genes:
NFkB IL1 IL1R MyD88 IRAK TRAF6 TAB TAK1 MEKK MKK JNK p38 cJun cFos AP1
MKP1 IL8 NIK IL6 IL6R GP130 PI3K JAK Akt mTOR SOCS3 MEK1_2 ERK1_2 Elk1 CEBPbeta

Transition functions:
NFkB = NFkB
IL1 = NFkB
IL1R = IL1
MyD88 = IL1R
IRAK = IL1R | MyD88 | IRAK
```

```
TRAF6 = IRAK
TAB = (TRAF6 | IRAK)
TAK1 = (TRAF6 | TAB)
MEKK = TRAF6
MKK = (TAK1 | MEKK)
JNK = MKK & ! MKP1
p38 = MKK & ! MKP1
cJun = (p38 | JNK | ERK1_2 | CEBPbeta) & cFos
cFos = p38 | JNK | Elk1 | CEBPbeta | STAT3
AP1 = cJun & cFos
MKP1 = AP1
IL8 = NFkB | AP1 | CEBPbeta
NIK = TAK1
IL6 = (NFkB | ERK1_2 | CEBPbeta)
IL6R = IL6
GP130 = IL6
PI3K = JAK
JAK = IL6R & ! SOCS3
Akt = PI3K
mTOR = Akt
SOCS3 = STAT3
MEK1_2 = GP130 & IL6
ERK1_2 = MEK1_2 & IL6
Elk1 = ERK1_2
CEBPbeta = Elk1
STAT3 = JAK | (cFos & cJun) | mTOR
no. inflamm attractors[1] 6
```

Exhaustive search for attractors in the inflammation sub-network leads to 6 different attractors. In the following part the patterns in the attractors of inflammation sub-network and the complete network are compared.

```
par(mar=c(5,7,5,4))
#compare attractors of subparts and complete network
inflammGenes <- inflamm$genes #genes relevant for comparsion
excludedGenes2 <- saspnetwork$genes[!(saspnetwork$genes %in% inflammGenes)]

#create attractor matrices
saspAttrMatrices2 <- lapply(saspAttrs$attractors,
                            getStateMatrix,
                            genes = saspnetwork$genes,
                            excludedGenes = excludedGenes2)

#search corresponding vector of dnaDamage attractors in complete model
correspondingAttrsInflamm <- lapply(inflammAttrVecs, function(v) {
  matchingAttrs <- lapply(1:length(saspAttrMatrices2), function(m) {
    if(attractorExists(saspAttrMatrices2[[m]],
                       v,
                       partly = T))
      m
    else
      NULL
})})

#naming of attractor matchings
```

```r
names(correspondingAttrsInflamm) <- paste0("Inflamm Attr ",
                                           1:length(correspondingAttrsInflamm))
correspondingAttrsInflamm <- lapply(correspondingAttrsInflamm, function(a) {
  ret <- a
  names(ret) <- paste0("Complete Attr", 1:length(a))
  return(ret)})

#extract attractor IDs from complete subnetworks, found in inflammation network
foundInflamm <- unique(unlist(correspondingAttrsInflamm))

print("Matching attractors in complete network : ")
print(sort(foundInflamm))

#print matching attractor IDs
foundMatches <- gsub("\\.", " -> ", names(unlist(correspondingAttrsInflamm)))
invisible(sapply(foundMatches,print))

#plot matching attractors
matchingOrigs2 <- saspAttrs$attractors[foundInflamm]
matchingOrigs2 <- list(stateInfo = saspAttrs$stateInfo,
                       attractors = matchingOrigs2)
class(matchingOrigs2) <- "AttractorInfo"
par(mar=c(5,7,5,4))
p <- plotAttractors(matchingOrigs2,
                    title = "Attractors of SASP found in inflammation sub-network",
                    allInOnePlot = F,
                    offColor = "#7b3294",
                    onColor = "#a6dba0")
```

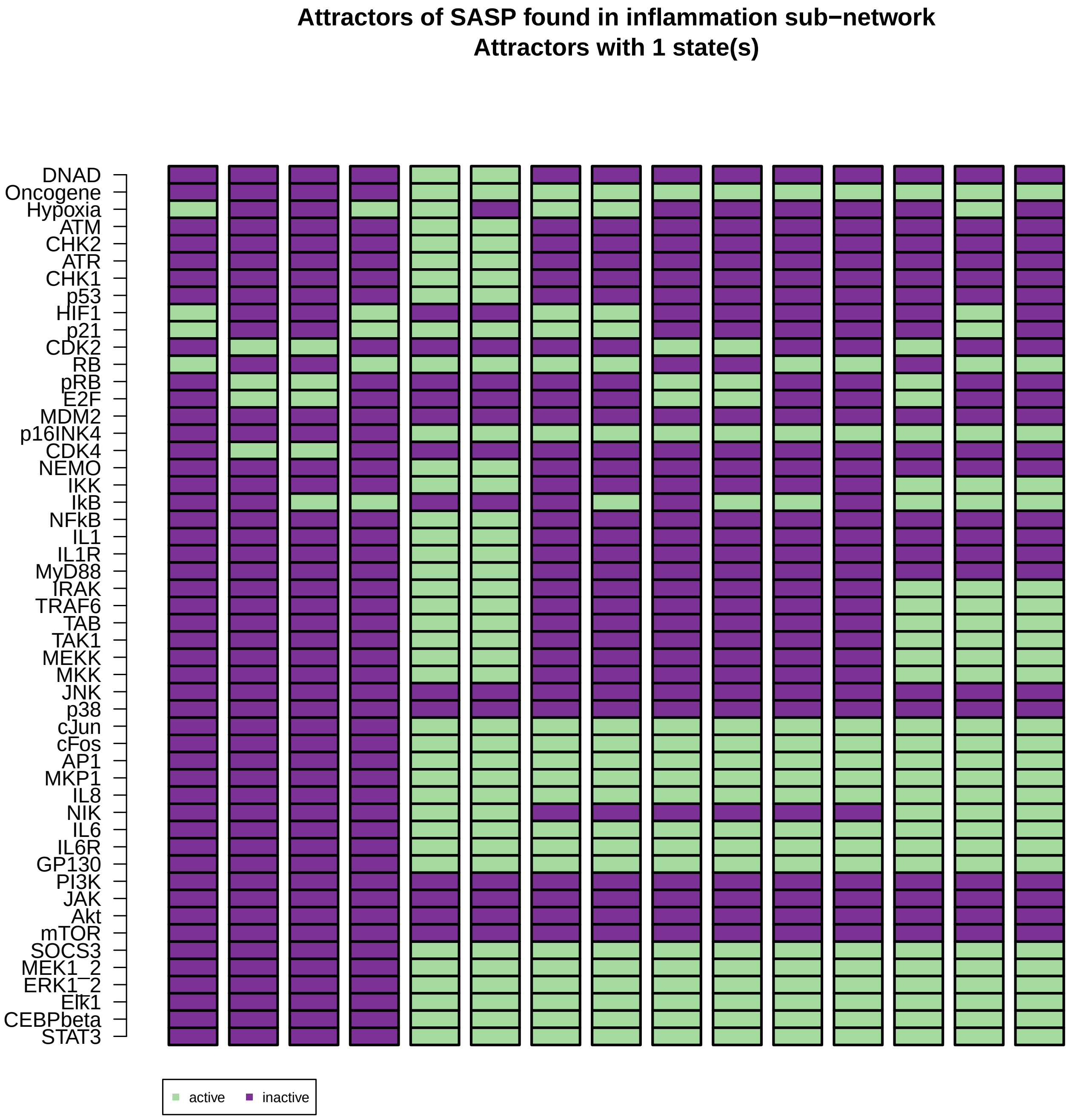

Attractors of SASP found in inflammation sub−network
Attractors with 1 state(s)
DNAD
Oncogene
Hypoxia
ATM
CHK2
ATR
CHK1
p53
HIF1
p21
CDK2
RB
pRB
E2F
MDM2
p16INK4
CDK4
NEMO
IKK
IkB
NFkB
IL1
IL1R
MyD88
IRAK
TRAF6
TAB
TAK1
MEKK
MKK
JNK
p38
cJun
cFos
AP1
MKP1
IL8
NIK
IL6
IL6R
GP130
PI3K
JAK
Akt
mTOR
SOCS3
MEK1_2
ERK1_2
Elk1
CEBPbeta
STAT3
active
inactive

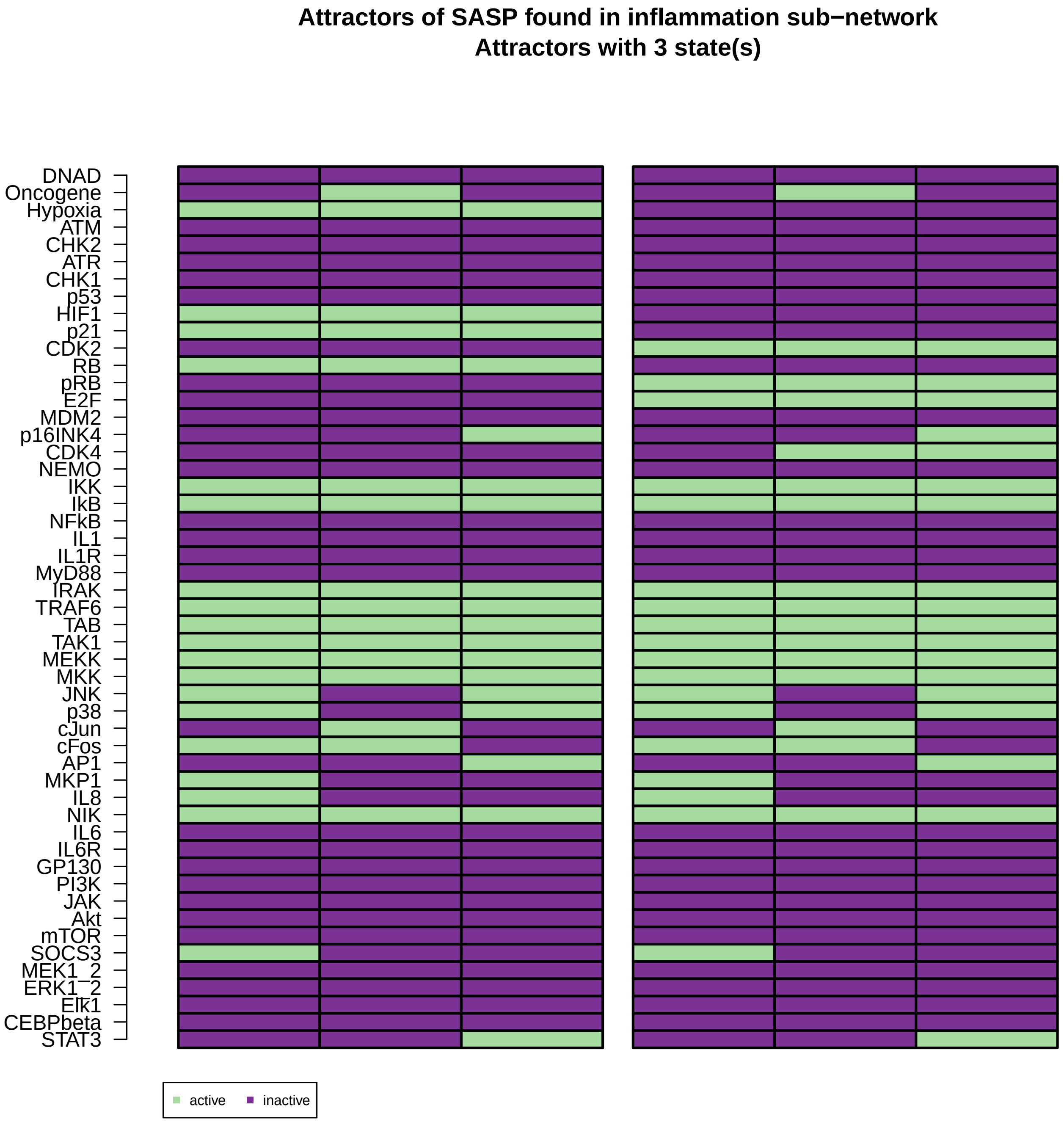
Attractors of SASP found in inflammation sub−network
Attractors with 3 state(s)
DNAD
Oncogene
Hypoxia
ATM
CHK2
ATR
CHK1
p53
HIF1
p21
CDK2
RB
pRB
E2F
MDM2
p16INK4
CDK4
NEMO
IKK
IkB
NFkB
IL1
IL1R
MyD88
IRAK
TRAF6
TAB
TAK1
MEKK
MKK
JNK
p38
cJun
cFos
AP1
MKP1
IL8
NIK
IL6
IL6R
GP130
PI3K
JAK
Akt
mTOR
SOCS3
MEK1_2
ERK1_2
Elk1
CEBPbeta
STAT3
active
inactive

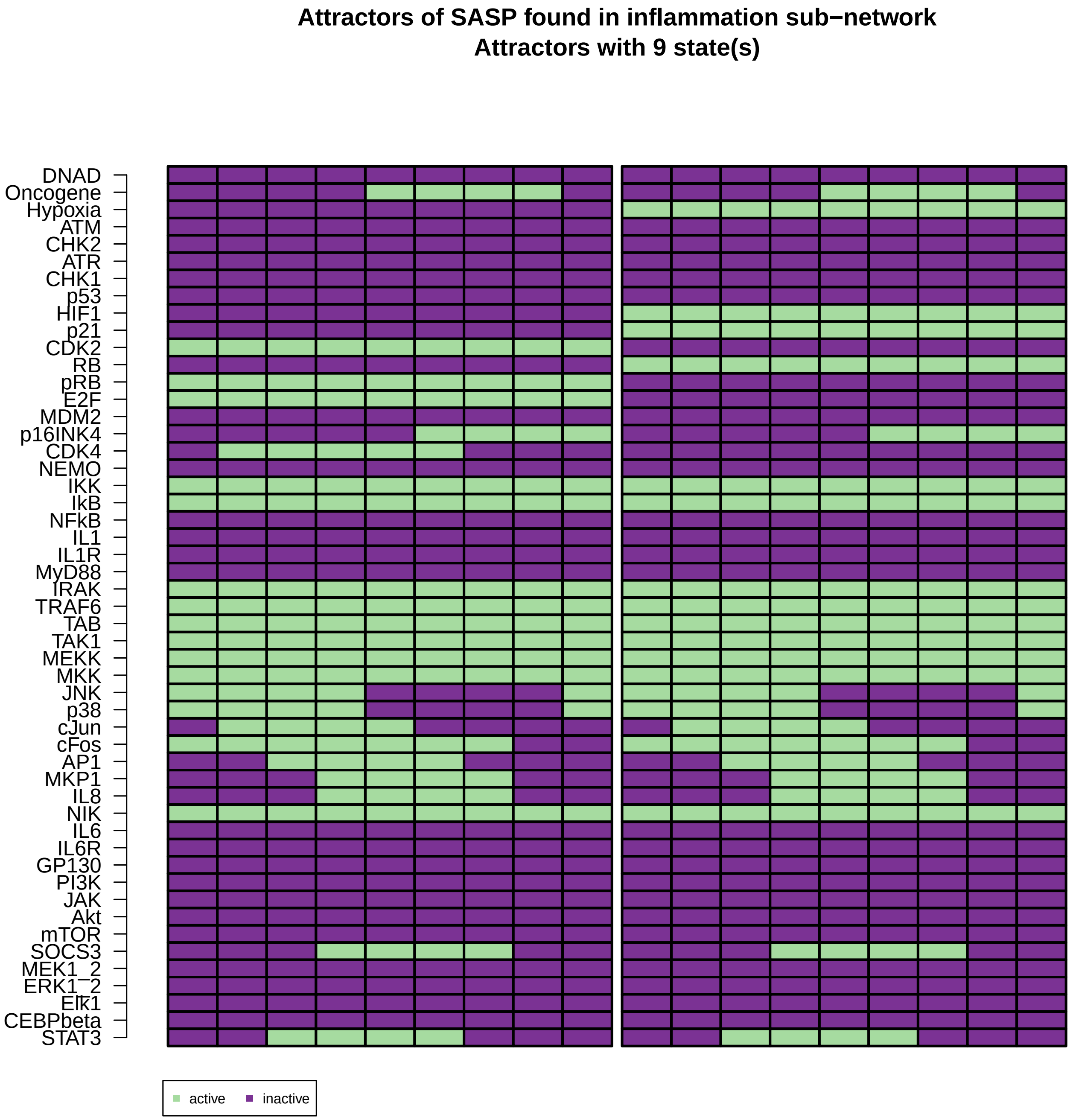


```
[1] "Matching attractors in complete network : "
 [1]  1  2  3  4  5  6  7  8  9 10 11 12 13 14 15 16 17 18 19
[1] "Inflamm Attr 1 -> Complete Attr1"
[1] "Inflamm Attr 1 -> Complete Attr2"
[1] "Inflamm Attr 1 -> Complete Attr3"
[1] "Inflamm Attr 1 -> Complete Attr4"
[1] "Inflamm Attr 2 -> Complete Attr5"
[1] "Inflamm Attr 2 -> Complete Attr6"
[1] "Inflamm Attr 3 -> Complete Attr9"
[1] "Inflamm Attr 3 -> Complete Attr10"
[1] "Inflamm Attr 3 -> Complete Attr11"
[1] "Inflamm Attr 3 -> Complete Attr12"
```

```
[1] "Inflamm Attr 3 -> Complete Attr13"
[1] "Inflamm Attr 3 -> Complete Attr14"
[1] "Inflamm Attr 4 -> Complete Attr7"
[1] "Inflamm Attr 4 -> Complete Attr8"
[1] "Inflamm Attr 4 -> Complete Attr15"
[1] "Inflamm Attr 5 -> Complete Attr16"
[1] "Inflamm Attr 5 -> Complete Attr17"
[1] "Inflamm Attr 6 -> Complete Attr18"
[1] "Inflamm Attr 6 -> Complete Attr19"
```

Simulation of complete network under physiological conditions

Figures 2 and 3 in the main manuscript show the signaling cascade for known physiological phenotypes.

```
startState <- generateState(saspnetwork ,c("DNAD" = 0, "IkB" = 1, "MDM2" = 1))
titleString <- startState[c("DNAD", "IkB", "MDM2")]
titleString <- paste(paste(names(titleString), titleString, sep = "="), collapse = " ")
par(mar=c(5,7,5,4))
invisible(plotSequence(saspnetwork,
                       startState = startState,
                       title = titleString,
                       offColor = "#7b3294", onColor = "#a6dba0"))
```

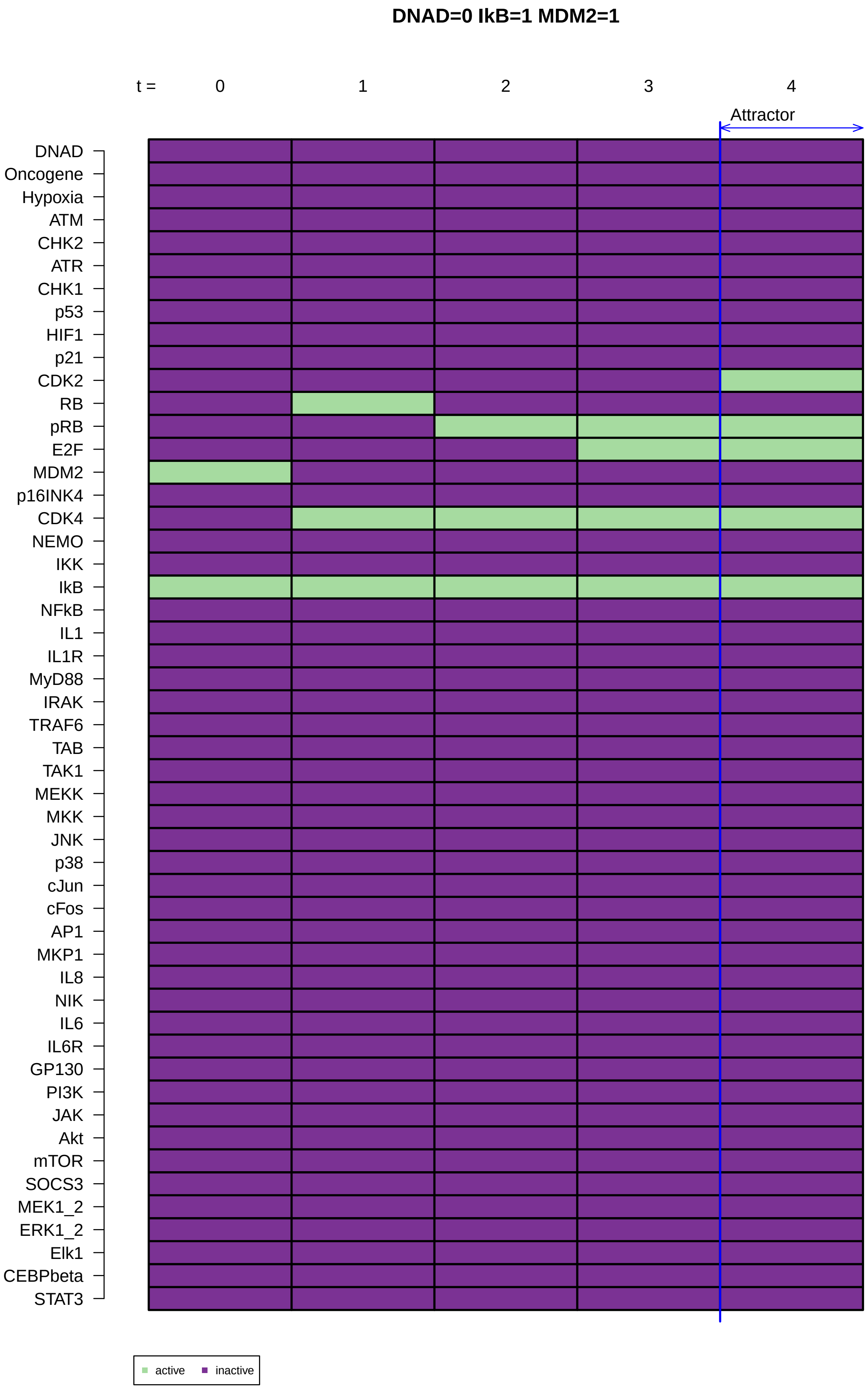
DNAD=0 IkB=1 MDM2=1
t =
0
1
2
3
4
Attractor
DNAD
Oncogene
Hypoxia
ATM
CHK2
ATR
CHK1
p53
HIF1
p21
CDK2
RB
pRB
E2F
MDM2
p16INK4
CDK4
NEMO
IKK
IkB
NFkB
IL1
IL1R
MyD88
IRAK
TRAF6
TAB
TAK1
MEKK
MKK
JNK
p38
cJun
cFos
AP1
MKP1
IL8
NIK
IL6
IL6R
GP130
PI3K
JAK
Akt
mTOR
SOCS3
MEK1_2
ERK1_2
Elk1
CEBPbeta
STAT3
active
inactive

Without DNA damage the resulting network state is expected to show normal cell cycle progression. As shown here in Figure 2, this includes the activation of CDK2 (t = 5) and CDK4 (t = 2) with subsequent hyperphosphorylation of RB that leads to a release and activation of E2F that in turn drives the cell into cell cycle progression.

Simulation of complete network under DNA damage conditions

```
startState <- generateState(saspnetwork, c("DNAD" = 1, "IkB" = 1, "MDM2" = 1))
titleString <- startState[c("DNAD", "IkB", "MDM2")]
titleString <- paste(paste(names(titleString), titleString, sep = "="), collapse = " ")
par(mar=c(5,7,5,4))
invisible(plotSequence(saspnetwork,
                       startState = startState,
                       title = titleString,
                       offColor = "#7b3294", onColor = "#a6dba0"))
```

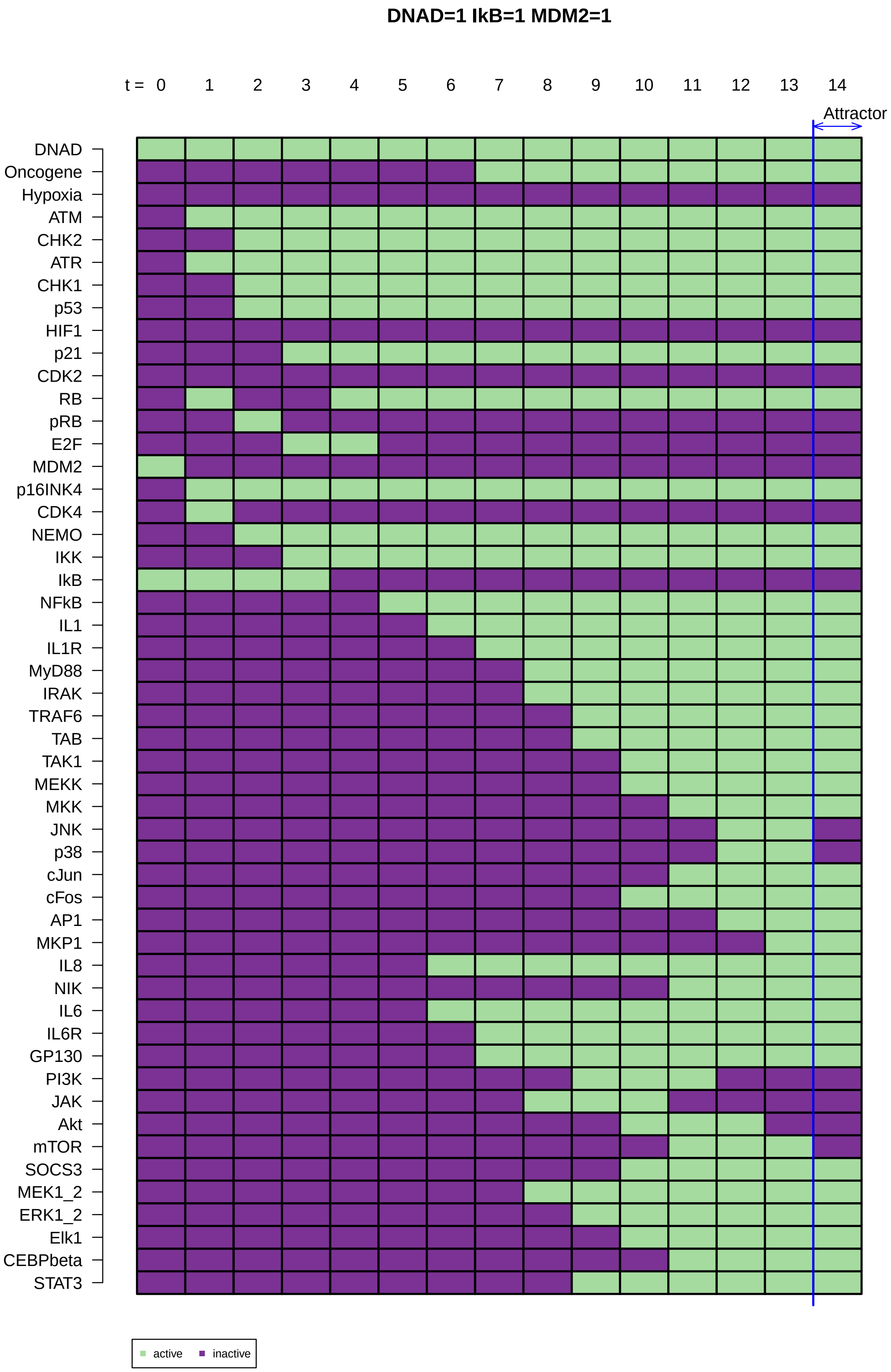
DNAD=1 IkB=1 MDM2=1
t = 0 1 2 3 4 5 6 7 8 9 10 11 12 13 14
Attractor
DNAD
Oncogene
Hypoxia
ATM
CHK2
ATR
CHK1
p53
HIF1
p21
CDK2
RB
pRB
E2F
MDM2
p16INK4
CDK4
NEMO
IKK
IkB
NFkB
IL1
IL1R
MyD88
IRAK
TRAF6
TAB
TAK1
MEKK
MKK
JNK
p38
cJun
cFos
AP1
MKP1
IL8
NIK
IL6
IL6R
GP130
PI3K
JAK
Akt
mTOR
SOCS3
MEK1_2
ERK1_2
Elk1
CEBPbeta
STAT3
active
inactive

Upon DNA damage the first response of the cell is the activation of ATM/ATR mediated DNA damage repair with a subsequent activation of p53- and p16-mediated cell cycle arrest. The DNA damage signal is relayed by the DNA damage response through NEMO that in turn activates NF-κB signaling which will ultimately lead to the activation of IL-1, IL-6 and IL-8 signaling.

Figures 4-6 in the main manuscript show the proposed knockouts that cause in-silico IL-6 and IL-8 inhibition. In each, we fix one of regulatory factors `NFkB`, `IkB` and `NEMO` to be `1` (over-expression) or `0` knock-out. These simulations show the evolution of expected states after DNA damage.

```
saspKO <- fixGenes(saspnetwork, "NFkB", 0)
startState <- generateState(saspKO, c("DNAD" = 1, "IkB" = 1, "MDM2" = 1))
titleString <- startState[c("DNAD", "IkB", "MDM2")]
titleString <- paste(paste(names(titleString), titleString, sep = "="), collapse = " ")
par(mar=c(5,7,5,4))
invisible(plotSequence(saspKO,
                       startState = startState,
                       title = titleString,
                       offColor = "#7b3294", onColor = "#a6dba0"))
```

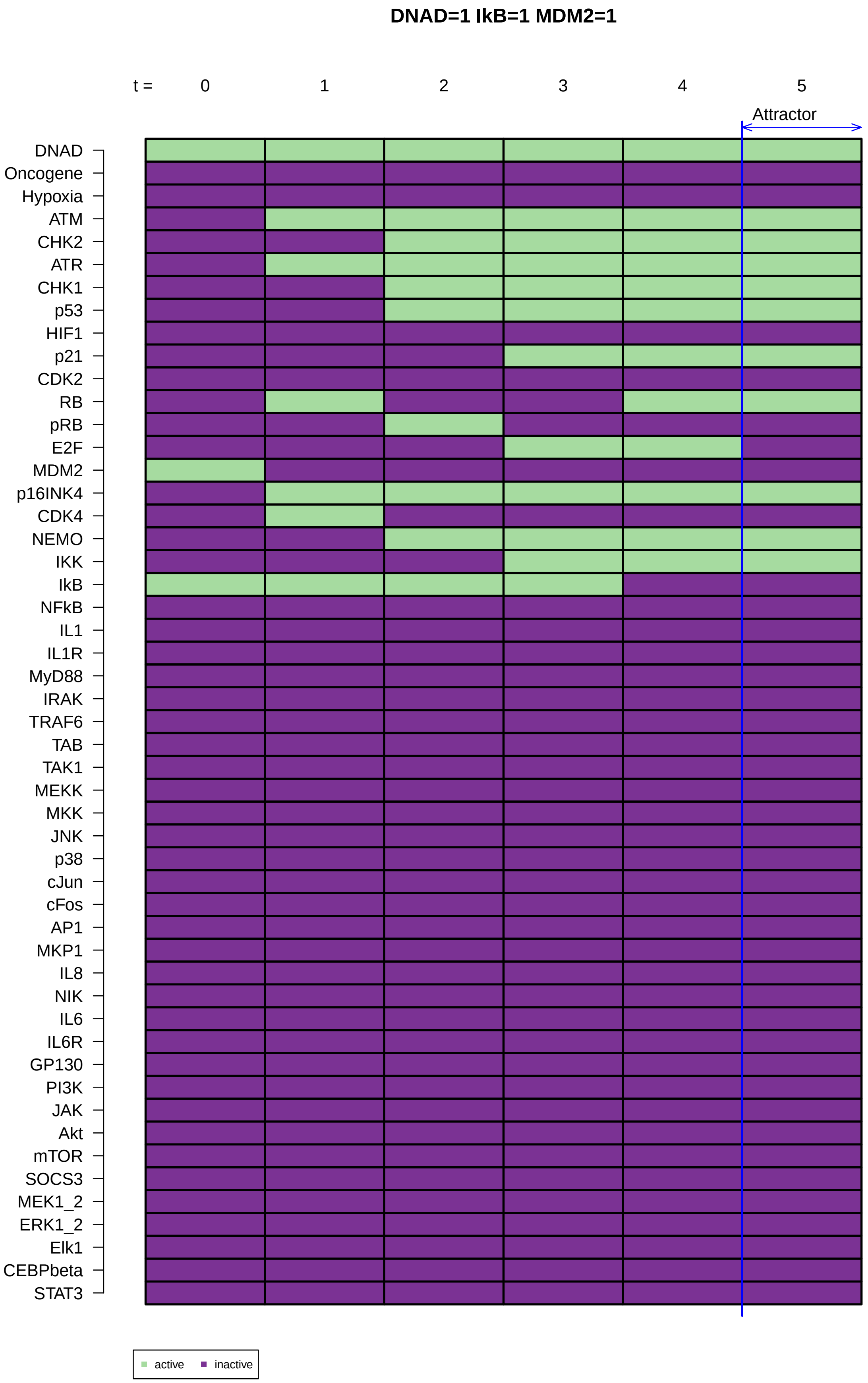

DNAD=1 IkB=1 MDM2=1
t =
0
1
2
3
4
5
Attractor
DNAD
Oncogene
Hypoxia
ATM
CHK2
ATR
CHK1
p53
HIF1
p21
CDK2
RB
pRB
E2F
MDM2
p16INK4
CDK4
NEMO
IKK
IkB
NFkB
IL1
IL1R
MyD88
IRAK
TRAF6
TAB
TAK1
MEKK
MKK
JNK
p38
cJun
cFos
AP1
MKP1
IL8
NIK
IL6
IL6R
GP130
PI3K
JAK
Akt
mTOR
SOCS3
MEK1_2
ERK1_2
Elk1
CEBPbeta
STAT3
active
inactive

```r
saspKO <- fixGenes(saspnetwork, "IkB", 1)
startState <- generateState(saspKO, c("DNAD" = 1, "IkB" = 1, "MDM2" = 1))
titleString <- startState[c("DNAD", "IkB", "MDM2")]
titleString <- paste(paste(names(titleString), titleString, sep = "="), collapse = " ")
par(mar=c(5,7,5,4))
invisible(plotSequence(saspKO,
                       startState = startState,
                       title = titleString,
                       offColor = "#7b3294", onColor = "#a6dba0"))
```

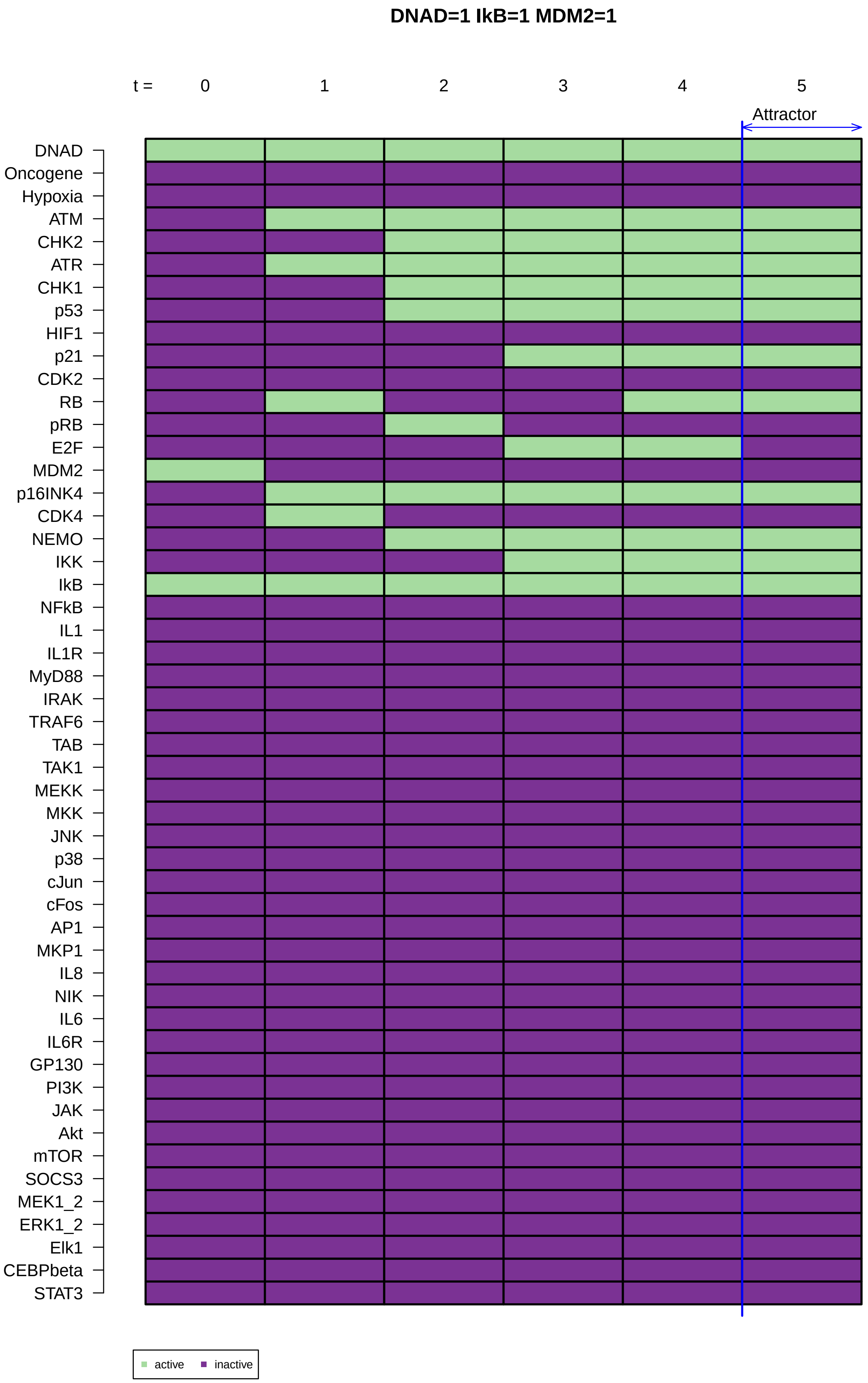
DNAD=1 IkB=1 MDM2=1
t =
0
1
2
3
4
5
Attractor
DNAD
Oncogene
Hypoxia
ATM
CHK2
ATR
CHK1
p53
HIF1
p21
CDK2
RB
pRB
E2F
MDM2
p16INK4
CDK4
NEMO
IKK
IkB
NFkB
IL1
IL1R
MyD88
IRAK
TRAF6
TAB
TAK1
MEKK
MKK
JNK
p38
cJun
cFos
AP1
MKP1
IL8
NIK
IL6
IL6R
GP130
PI3K
JAK
Akt
mTOR
SOCS3
MEK1_2
ERK1_2
Elk1
CEBPbeta
STAT3
active
inactive

```r
saspKO <- fixGenes(saspnetwork, "NEMO", 0)
startState <- generateState(saspKO, c("DNAD" = 1, "IkB" = 1, "MDM2" = 1))
titleString <- startState[c("DNAD", "IkB", "MDM2")]
titleString <- paste(paste(names(titleString), titleString, sep = "="), collapse = " ")
par(mar=c(5,7,5,4))
invisible(plotSequence(saspKO,
                       startState = startState,
                       title = titleString,
                       offColor = "#7b3294", onColor = "#a6dba0"))
```

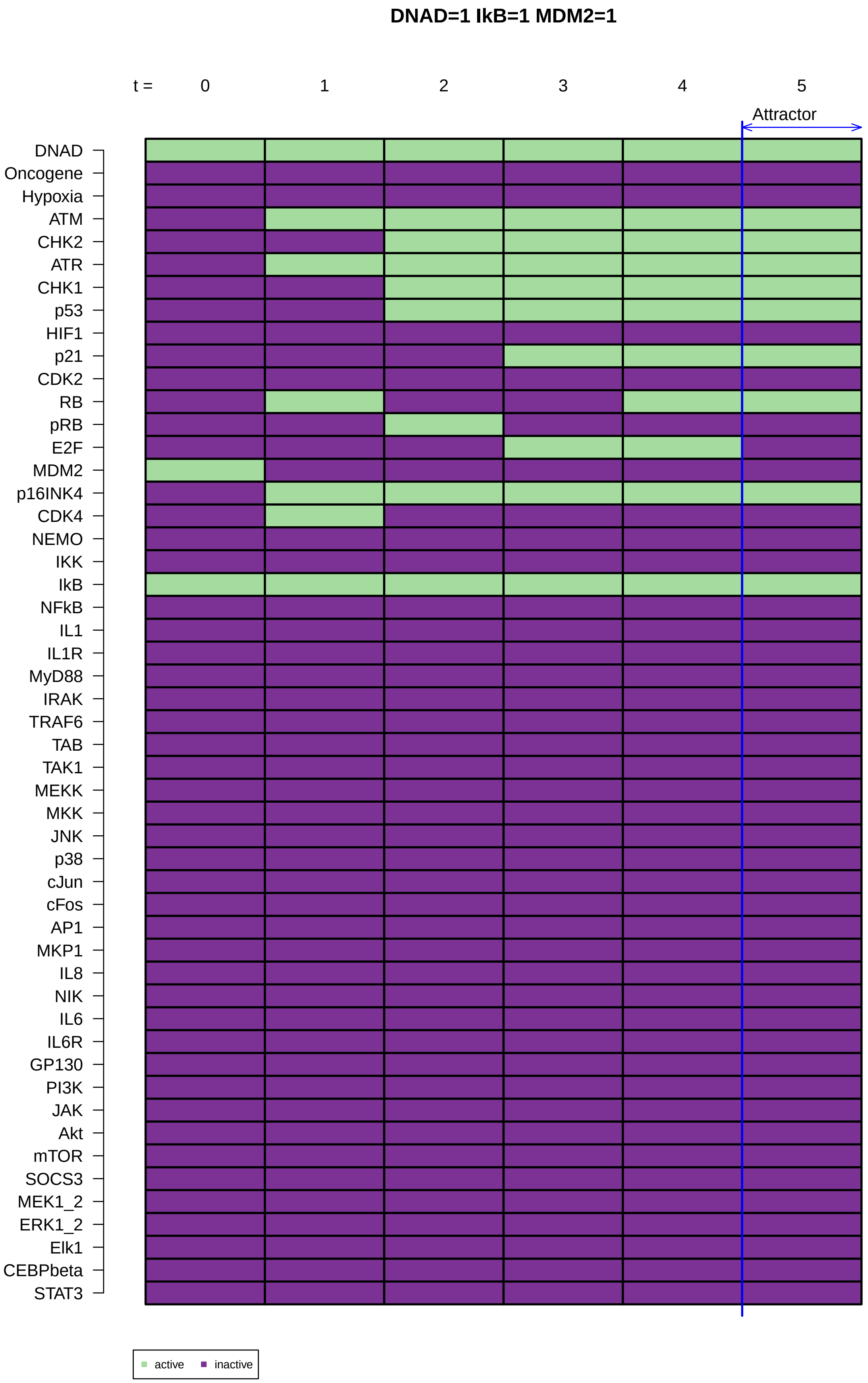
DNAD=1 IkB=1 MDM2=1
t =
0
1
2
3
4
5
Attractor
DNAD
Oncogene
Hypoxia
ATM
CHK2
ATR
CHK1
p53
HIF1
p21
CDK2
RB
pRB
E2F
MDM2
p16INK4
CDK4
NEMO
IKK
IkB
NFkB
IL1
IL1R
MyD88
IRAK
TRAF6
TAB
TAK1
MEKK
MKK
JNK
p38
cJun
cFos
AP1
MKP1
IL8
NIK
IL6
IL6R
GP130
PI3K
JAK
Akt
mTOR
SOCS3
MEK1_2
ERK1_2
Elk1
CEBPbeta
STAT3
active
inactive

Interaction targets that suppress IL signaling

One goal of the model is to generate candidate interactions that have suppressive effect on IL-6 and IL-8 activation. The following shows the implementation details on how to find such interactions.

```r
par(mar=c(5,7,5,4))
##
# This method finds candidate interactions that when modified lead to a
# hypothetical phenotype.
##
findCandidates <- function(network, startState,
                           numberOfInteractions = 1,
                           targetPhenotype = c("IL1" = 0, "IL6" = 0),
                           exclude = c("DNAD", "Hypoxia", "Stress")) {
  # first we select variable genes, i.e., genes that we can knock out
  # or overexpress in the model
  variableGenes <- setdiff(network$genes, c(names(targetPhenotype), exclude))

  # we then choose k = 'numberOfInteractions' out of variable genes
  geneCombinations <- combn(variableGenes, numberOfInteractions, simplify = F)

  # for each of the k combinations of genes we create a binary table
  # of k genes that represent the genes's different states
  binaryTables <- lapply(geneCombinations, function(x) {
    tbl <- BoolNet:::allcombn(2, length(x)) - 1
    colnames(tbl) <- x
    data.frame(as.data.frame(tbl),
               groupcombination = paste(x, collapse = "_"),
               valuecombination = 1:nrow(tbl))
  })

  # we then transform these tables into a long format that will allow us ...
  result <- lapply(binaryTables, tidyr::gather,
                   key = "gene", value = "value",
                   - groupcombination, - valuecombination) %>%
    dplyr::bind_rows() %>%
    # ... for each group and value combination ...
    dplyr::group_by(groupcombination, valuecombination) %>%
    tidyr::nest() %>%
    dplyr::mutate(targetSatisfied =
                    purrr::map(data, function(candidate) {
                      # ... to compute a path to an attractor.
                      # From the start state
                      s <- startState
                      # and each gene state in the group combination ...
                      s[candidate$gene] <- candidate$value

                      # ... we fix the gene, i.e. knock out or overexpress
                      # them in the network.
                      fixedNetwork <-  fixGenes(network,
                                                 candidate$gene,
                                                 candidate$value)
                      # We compute the attractor for this particular start
                      # state and furthermore the path of binary activation
```

```
                      # patterns that lead to this attractor
                      a <- getAttractorSequence(
                        getAttractors(fixedNetwork,
                                      startStates = list(s)), 1)
                      # We check if ALL the genes in the path to the
                      # attractor correspond to the desired targetPhenotype
                      return(all(mapply(function(gene, value) {
                        all(a[,gene] == value)
                      }, names(targetPhenotype), targetPhenotype)))
                    })) %>%
    tidyr::unnest(targetSatisfied, .drop = FALSE)
  return(result)
}
```

Having defined the search procedure we can apply it to our SASP network. As shown in previous section (Figure 3 in the main manuscript), activation of DNA damage (`DNAD`) together with corresponding factors `IkB` and `MDM2` leads to an activation of IL-6 and IL-8 signaling. The model allows us to generate hypotheses about the possible knockouts/over-expressions, which may lead to suppression of `IL-6` and `IL-8`.

```
par(mar=c(5,7,5,4))
# Start state that lead to activation of IL signalling
startState <- generateState(saspnetwork,c("DNAD" = 1, "IkB" = 1, "MDM2" = 1))
# Given the start state, find candidates that surpress IL6 and IL 8
combs <- findCandidates(network = saspnetwork, startState = startState,
                        # by looking at single knock outs
                        numberOfInteractions = 1,
                        targetPhenotype = c("IL8" = 0, "IL6" = 0))

knitr::kable(combs %>%
  dplyr::filter(targetSatisfied == TRUE) %>%
  tidyr::unnest() %>%
  dplyr::group_by(groupcombination, valuecombination) %>%
  tidyr::nest( - targetSatisfied) %>%
  dplyr::mutate(hypothesis = map(data, function(x) {
    paste(paste(x$gene, x$value, sep = " = "), collapse = "; ")
  })) %>%
  tidyr::unnest(hypothesis, .drop = TRUE)
)
```

groupcombination	valuecombination	hypothesis
ATM	1	ATM = 0
NEMO	1	NEMO = 0
IKK	1	IKK = 0
IkB	2	IkB = 1
NFkB	1	NFkB = 0

The table shows possible combinations. Since the knockout of `ATM`, `IKK`, and `NFkB` or over-expression of `IkB` in the actual biological system will have a negative impact on the cells, `NEMO` remains as a viable candidate. Therefore we choose knockout of `NEMO` as the hypothesis to test in wet-lab.

Hypotheses that include multiple interactions can be tested in a similar way. For example, there are 343 possible hypotheses for interactions including two regulatory factors. However, some of them will be a subset of the hypotheses that were generated for one regulatory factor, like `NEMO`.

```r
par(mar=c(5,7,5,4))
# Start state that lead to activation of IL signalling
startState <- generateState(saspnetwork,c("DNAD" = 1, "IkB" = 1, "MDM2" = 1))
# Given the start state, find candidates that surpress IL6 and IL 8
combs <- findCandidates(network = saspnetwork, startState = startState,
                        # by looking at single knock outs
                        numberOfInteractions = 2,
                        targetPhenotype = c("IL8" = 0, "IL6" = 0))

knitr::kable(combs %>%
  dplyr::filter(targetSatisfied == TRUE) %>%
  tidyr::unnest() %>%
  dplyr::group_by(groupcombination, valuecombination) %>%
  tidyr::nest( - targetSatisfied) %>%
  dplyr::mutate(hypothesis = map(data, function(x) {
    paste(paste(x$gene, x$value, sep = " = "), collapse = "; ")
  })) %>%
  tidyr::unnest(hypothesis, .drop = TRUE)
)
```

groupcombination	valuecombination	hypothesis
Oncogene_ATM	1	Oncogene = 0; ATM = 0
Oncogene_ATM	3	Oncogene = 1; ATM = 0
Oncogene_NEMO	1	Oncogene = 0; NEMO = 0
Oncogene_NEMO	3	Oncogene = 1; NEMO = 0
Oncogene_IKK	1	Oncogene = 0; IKK = 0
Oncogene_IKK	3	Oncogene = 1; IKK = 0
Oncogene_IkB	2	Oncogene = 0; IkB = 1
Oncogene_IkB	4	Oncogene = 1; IkB = 1
Oncogene_NFkB	1	Oncogene = 0; NFkB = 0
Oncogene_NFkB	3	Oncogene = 1; NFkB = 0
ATM_CHK2	1	ATM = 0; CHK2 = 0
ATM_CHK2	2	ATM = 0; CHK2 = 1
ATM_ATR	1	ATM = 0; ATR = 0
ATM_ATR	2	ATM = 0; ATR = 1
ATM_CHK1	1	ATM = 0; CHK1 = 0
ATM_CHK1	2	ATM = 0; CHK1 = 1
ATM_p53	1	ATM = 0; p53 = 0
ATM_p53	2	ATM = 0; p53 = 1
ATM_HIF1	1	ATM = 0; HIF1 = 0
ATM_HIF1	2	ATM = 0; HIF1 = 1
ATM_p21	1	ATM = 0; p21 = 0
ATM_p21	2	ATM = 0; p21 = 1
ATM_CDK2	1	ATM = 0; CDK2 = 0
ATM_CDK2	2	ATM = 0; CDK2 = 1
ATM_RB	1	ATM = 0; RB = 0
ATM_RB	2	ATM = 0; RB = 1
ATM_pRB	1	ATM = 0; pRB = 0
ATM_pRB	2	ATM = 0; pRB = 1
ATM_E2F	1	ATM = 0; E2F = 0
ATM_E2F	2	ATM = 0; E2F = 1
ATM_MDM2	1	ATM = 0; MDM2 = 0
ATM_MDM2	2	ATM = 0; MDM2 = 1

groupcombination	valuecombination	hypothesis
ATM_p16INK4	1	ATM = 0; p16INK4 = 0
ATM_p16INK4	2	ATM = 0; p16INK4 = 1
ATM_CDK4	1	ATM = 0; CDK4 = 0
ATM_CDK4	2	ATM = 0; CDK4 = 1
ATM_NEMO	1	ATM = 0; NEMO = 0
ATM_NEMO	3	ATM = 1; NEMO = 0
ATM_IKK	1	ATM = 0; IKK = 0
ATM_IKK	2	ATM = 0; IKK = 1
ATM_IKK	3	ATM = 1; IKK = 0
ATM_IkB	1	ATM = 0; IkB = 0
ATM_IkB	2	ATM = 0; IkB = 1
ATM_IkB	4	ATM = 1; IkB = 1
ATM_NFkB	1	ATM = 0; NFkB = 0
ATM_NFkB	3	ATM = 1; NFkB = 0
ATM_IL1	1	ATM = 0; IL1 = 0
ATM_IL1R	1	ATM = 0; IL1R = 0
ATM_MyD88	1	ATM = 0; MyD88 = 0
ATM_IRAK	1	ATM = 0; IRAK = 0
ATM_TRAF6	1	ATM = 0; TRAF6 = 0
ATM_TAB	1	ATM = 0; TAB = 0
ATM_TAK1	1	ATM = 0; TAK1 = 0
ATM_MEKK	1	ATM = 0; MEKK = 0
ATM_MKK	1	ATM = 0; MKK = 0
ATM_JNK	1	ATM = 0; JNK = 0
ATM_p38	1	ATM = 0; p38 = 0
ATM_cJun	1	ATM = 0; cJun = 0
ATM_cJun	2	ATM = 0; cJun = 1
ATM_cFos	1	ATM = 0; cFos = 0
ATM_cFos	2	ATM = 0; cFos = 1
ATM_AP1	1	ATM = 0; AP1 = 0
ATM_MKP1	1	ATM = 0; MKP1 = 0
ATM_MKP1	2	ATM = 0; MKP1 = 1
ATM_NIK	1	ATM = 0; NIK = 0
ATM_NIK	2	ATM = 0; NIK = 1
ATM_IL6R	1	ATM = 0; IL6R = 0
ATM_IL6R	2	ATM = 0; IL6R = 1
ATM_GP130	1	ATM = 0; GP130 = 0
ATM_GP130	2	ATM = 0; GP130 = 1
ATM_PI3K	1	ATM = 0; PI3K = 0
ATM_PI3K	2	ATM = 0; PI3K = 1
ATM_JAK	1	ATM = 0; JAK = 0
ATM_JAK	2	ATM = 0; JAK = 1
ATM_Akt	1	ATM = 0; Akt = 0
ATM_Akt	2	ATM = 0; Akt = 1
ATM_mTOR	1	ATM = 0; mTOR = 0
ATM_mTOR	2	ATM = 0; mTOR = 1
ATM_SOCS3	1	ATM = 0; SOCS3 = 0
ATM_SOCS3	2	ATM = 0; SOCS3 = 1
ATM_MEK1_2	1	ATM = 0; MEK1_2 = 0
ATM_MEK1_2	2	ATM = 0; MEK1_2 = 1
ATM_ERK1_2	1	ATM = 0; ERK1_2 = 0
ATM_Elk1	1	ATM = 0; Elk1 = 0

groupcombination	valuecombination	hypothesis
ATM__CEBPbeta	1	ATM = 0; CEBPbeta = 0
ATM__STAT3	1	ATM = 0; STAT3 = 0
ATM__STAT3	2	ATM = 0; STAT3 = 1
CHK2__NEMO	1	CHK2 = 0; NEMO = 0
CHK2__NEMO	3	CHK2 = 1; NEMO = 0
CHK2__IKK	1	CHK2 = 0; IKK = 0
CHK2__IKK	3	CHK2 = 1; IKK = 0
CHK2__IkB	2	CHK2 = 0; IkB = 1
CHK2__IkB	4	CHK2 = 1; IkB = 1
CHK2__NFkB	1	CHK2 = 0; NFkB = 0
CHK2__NFkB	3	CHK2 = 1; NFkB = 0
ATR__NEMO	1	ATR = 0; NEMO = 0
ATR__NEMO	3	ATR = 1; NEMO = 0
ATR__IKK	1	ATR = 0; IKK = 0
ATR__IKK	3	ATR = 1; IKK = 0
ATR__IkB	2	ATR = 0; IkB = 1
ATR__IkB	4	ATR = 1; IkB = 1
ATR__NFkB	1	ATR = 0; NFkB = 0
ATR__NFkB	3	ATR = 1; NFkB = 0
CHK1__NEMO	1	CHK1 = 0; NEMO = 0
CHK1__NEMO	3	CHK1 = 1; NEMO = 0
CHK1__IKK	1	CHK1 = 0; IKK = 0
CHK1__IKK	3	CHK1 = 1; IKK = 0
CHK1__IkB	2	CHK1 = 0; IkB = 1
CHK1__IkB	4	CHK1 = 1; IkB = 1
CHK1__NFkB	1	CHK1 = 0; NFkB = 0
CHK1__NFkB	3	CHK1 = 1; NFkB = 0
p53__NEMO	1	p53 = 0; NEMO = 0
p53__NEMO	3	p53 = 1; NEMO = 0
p53__IKK	1	p53 = 0; IKK = 0
p53__IKK	3	p53 = 1; IKK = 0
p53__IkB	2	p53 = 0; IkB = 1
p53__IkB	4	p53 = 1; IkB = 1
p53__NFkB	1	p53 = 0; NFkB = 0
p53__NFkB	3	p53 = 1; NFkB = 0
HIF1__NEMO	1	HIF1 = 0; NEMO = 0
HIF1__NEMO	3	HIF1 = 1; NEMO = 0
HIF1__IKK	1	HIF1 = 0; IKK = 0
HIF1__IKK	3	HIF1 = 1; IKK = 0
HIF1__IkB	2	HIF1 = 0; IkB = 1
HIF1__IkB	4	HIF1 = 1; IkB = 1
HIF1__NFkB	1	HIF1 = 0; NFkB = 0
HIF1__NFkB	3	HIF1 = 1; NFkB = 0
p21__NEMO	1	p21 = 0; NEMO = 0
p21__NEMO	3	p21 = 1; NEMO = 0
p21__IKK	1	p21 = 0; IKK = 0
p21__IKK	3	p21 = 1; IKK = 0
p21__IkB	2	p21 = 0; IkB = 1
p21__IkB	4	p21 = 1; IkB = 1
p21__NFkB	1	p21 = 0; NFkB = 0
p21__NFkB	3	p21 = 1; NFkB = 0
CDK2__NEMO	1	CDK2 = 0; NEMO = 0

groupcombination	valuecombination	hypothesis
CDK2__NEMO	3	CDK2 = 1; NEMO = 0
CDK2__IKK	1	CDK2 = 0; IKK = 0
CDK2__IKK	3	CDK2 = 1; IKK = 0
CDK2__IkB	2	CDK2 = 0; IkB = 1
CDK2__IkB	4	CDK2 = 1; IkB = 1
CDK2__NFkB	1	CDK2 = 0; NFkB = 0
CDK2__NFkB	3	CDK2 = 1; NFkB = 0
RB__NEMO	1	RB = 0; NEMO = 0
RB__NEMO	3	RB = 1; NEMO = 0
RB__IKK	1	RB = 0; IKK = 0
RB__IKK	3	RB = 1; IKK = 0
RB__IkB	2	RB = 0; IkB = 1
RB__IkB	4	RB = 1; IkB = 1
RB__NFkB	1	RB = 0; NFkB = 0
RB__NFkB	3	RB = 1; NFkB = 0
pRB__NEMO	1	pRB = 0; NEMO = 0
pRB__NEMO	3	pRB = 1; NEMO = 0
pRB__IKK	1	pRB = 0; IKK = 0
pRB__IKK	3	pRB = 1; IKK = 0
pRB__IkB	2	pRB = 0; IkB = 1
pRB__IkB	4	pRB = 1; IkB = 1
pRB__NFkB	1	pRB = 0; NFkB = 0
pRB__NFkB	3	pRB = 1; NFkB = 0
E2F__NEMO	1	E2F = 0; NEMO = 0
E2F__NEMO	3	E2F = 1; NEMO = 0
E2F__IKK	1	E2F = 0; IKK = 0
E2F__IKK	3	E2F = 1; IKK = 0
E2F__IkB	2	E2F = 0; IkB = 1
E2F__IkB	4	E2F = 1; IkB = 1
E2F__NFkB	1	E2F = 0; NFkB = 0
E2F__NFkB	3	E2F = 1; NFkB = 0
MDM2__NEMO	1	MDM2 = 0; NEMO = 0
MDM2__NEMO	3	MDM2 = 1; NEMO = 0
MDM2__IKK	1	MDM2 = 0; IKK = 0
MDM2__IKK	3	MDM2 = 1; IKK = 0
MDM2__IkB	2	MDM2 = 0; IkB = 1
MDM2__IkB	4	MDM2 = 1; IkB = 1
MDM2__NFkB	1	MDM2 = 0; NFkB = 0
MDM2__NFkB	3	MDM2 = 1; NFkB = 0
p16INK4__NEMO	1	p16INK4 = 0; NEMO = 0
p16INK4__NEMO	3	p16INK4 = 1; NEMO = 0
p16INK4__IKK	1	p16INK4 = 0; IKK = 0
p16INK4__IKK	3	p16INK4 = 1; IKK = 0
p16INK4__IkB	2	p16INK4 = 0; IkB = 1
p16INK4__IkB	4	p16INK4 = 1; IkB = 1
p16INK4__NFkB	1	p16INK4 = 0; NFkB = 0
p16INK4__NFkB	3	p16INK4 = 1; NFkB = 0
CDK4__NEMO	1	CDK4 = 0; NEMO = 0
CDK4__NEMO	3	CDK4 = 1; NEMO = 0
CDK4__IKK	1	CDK4 = 0; IKK = 0
CDK4__IKK	3	CDK4 = 1; IKK = 0
CDK4__IkB	2	CDK4 = 0; IkB = 1

groupcombination	valuecombination	hypothesis
CDK4_IkB	4	CDK4 = 1; IkB = 1
CDK4_NFkB	1	CDK4 = 0; NFkB = 0
CDK4_NFkB	3	CDK4 = 1; NFkB = 0
NEMO_IKK	1	NEMO = 0; IKK = 0
NEMO_IKK	2	NEMO = 0; IKK = 1
NEMO_IKK	3	NEMO = 1; IKK = 0
NEMO_IkB	1	NEMO = 0; IkB = 0
NEMO_IkB	2	NEMO = 0; IkB = 1
NEMO_IkB	4	NEMO = 1; IkB = 1
NEMO_NFkB	1	NEMO = 0; NFkB = 0
NEMO_NFkB	3	NEMO = 1; NFkB = 0
NEMO_IL1	1	NEMO = 0; IL1 = 0
NEMO_IL1R	1	NEMO = 0; IL1R = 0
NEMO_MyD88	1	NEMO = 0; MyD88 = 0
NEMO_IRAK	1	NEMO = 0; IRAK = 0
NEMO_TRAF6	1	NEMO = 0; TRAF6 = 0
NEMO_TAB	1	NEMO = 0; TAB = 0
NEMO_TAK1	1	NEMO = 0; TAK1 = 0
NEMO_MEKK	1	NEMO = 0; MEKK = 0
NEMO_MKK	1	NEMO = 0; MKK = 0
NEMO_JNK	1	NEMO = 0; JNK = 0
NEMO_p38	1	NEMO = 0; p38 = 0
NEMO_cJun	1	NEMO = 0; cJun = 0
NEMO_cJun	2	NEMO = 0; cJun = 1
NEMO_cFos	1	NEMO = 0; cFos = 0
NEMO_cFos	2	NEMO = 0; cFos = 1
NEMO_AP1	1	NEMO = 0; AP1 = 0
NEMO_MKP1	1	NEMO = 0; MKP1 = 0
NEMO_MKP1	2	NEMO = 0; MKP1 = 1
NEMO_NIK	1	NEMO = 0; NIK = 0
NEMO_NIK	2	NEMO = 0; NIK = 1
NEMO_IL6R	1	NEMO = 0; IL6R = 0
NEMO_IL6R	2	NEMO = 0; IL6R = 1
NEMO_GP130	1	NEMO = 0; GP130 = 0
NEMO_GP130	2	NEMO = 0; GP130 = 1
NEMO_PI3K	1	NEMO = 0; PI3K = 0
NEMO_PI3K	2	NEMO = 0; PI3K = 1
NEMO_JAK	1	NEMO = 0; JAK = 0
NEMO_JAK	2	NEMO = 0; JAK = 1
NEMO_Akt	1	NEMO = 0; Akt = 0
NEMO_Akt	2	NEMO = 0; Akt = 1
NEMO_mTOR	1	NEMO = 0; mTOR = 0
NEMO_mTOR	2	NEMO = 0; mTOR = 1
NEMO_SOCS3	1	NEMO = 0; SOCS3 = 0
NEMO_SOCS3	2	NEMO = 0; SOCS3 = 1
NEMO_MEK1_2	1	NEMO = 0; MEK1_2 = 0
NEMO_MEK1_2	2	NEMO = 0; MEK1_2 = 1
NEMO_ERK1_2	1	NEMO = 0; ERK1_2 = 0
NEMO_Elk1	1	NEMO = 0; Elk1 = 0
NEMO_CEBPbeta	1	NEMO = 0; CEBPbeta = 0
NEMO_STAT3	1	NEMO = 0; STAT3 = 0
NEMO_STAT3	2	NEMO = 0; STAT3 = 1

groupcombination	valuecombination	hypothesis
IKK_IkB	1	IKK = 0; IkB = 0
IKK_IkB	2	IKK = 0; IkB = 1
IKK_IkB	4	IKK = 1; IkB = 1
IKK_NFkB	1	IKK = 0; NFkB = 0
IKK_NFkB	3	IKK = 1; NFkB = 0
IKK_IL1	1	IKK = 0; IL1 = 0
IKK_IL1R	1	IKK = 0; IL1R = 0
IKK_MyD88	1	IKK = 0; MyD88 = 0
IKK_IRAK	1	IKK = 0; IRAK = 0
IKK_TRAF6	1	IKK = 0; TRAF6 = 0
IKK_TAB	1	IKK = 0; TAB = 0
IKK_TAK1	1	IKK = 0; TAK1 = 0
IKK_MEKK	1	IKK = 0; MEKK = 0
IKK_MKK	1	IKK = 0; MKK = 0
IKK_JNK	1	IKK = 0; JNK = 0
IKK_p38	1	IKK = 0; p38 = 0
IKK_cJun	1	IKK = 0; cJun = 0
IKK_cJun	2	IKK = 0; cJun = 1
IKK_cFos	1	IKK = 0; cFos = 0
IKK_cFos	2	IKK = 0; cFos = 1
IKK_AP1	1	IKK = 0; AP1 = 0
IKK_MKP1	1	IKK = 0; MKP1 = 0
IKK_MKP1	2	IKK = 0; MKP1 = 1
IKK_NIK	1	IKK = 0; NIK = 0
IKK_NIK	2	IKK = 0; NIK = 1
IKK_IL6R	1	IKK = 0; IL6R = 0
IKK_IL6R	2	IKK = 0; IL6R = 1
IKK_GP130	1	IKK = 0; GP130 = 0
IKK_GP130	2	IKK = 0; GP130 = 1
IKK_PI3K	1	IKK = 0; PI3K = 0
IKK_PI3K	2	IKK = 0; PI3K = 1
IKK_JAK	1	IKK = 0; JAK = 0
IKK_JAK	2	IKK = 0; JAK = 1
IKK_Akt	1	IKK = 0; Akt = 0
IKK_Akt	2	IKK = 0; Akt = 1
IKK_mTOR	1	IKK = 0; mTOR = 0
IKK_mTOR	2	IKK = 0; mTOR = 1
IKK_SOCS3	1	IKK = 0; SOCS3 = 0
IKK_SOCS3	2	IKK = 0; SOCS3 = 1
IKK_MEK1_2	1	IKK = 0; MEK1_2 = 0
IKK_MEK1_2	2	IKK = 0; MEK1_2 = 1
IKK_ERK1_2	1	IKK = 0; ERK1_2 = 0
IKK_Elk1	1	IKK = 0; Elk1 = 0
IKK_CEBPbeta	1	IKK = 0; CEBPbeta = 0
IKK_STAT3	1	IKK = 0; STAT3 = 0
IKK_STAT3	2	IKK = 0; STAT3 = 1
IkB_NFkB	1	IkB = 0; NFkB = 0
IkB_NFkB	3	IkB = 1; NFkB = 0
IkB_IL1	3	IkB = 1; IL1 = 0
IkB_IL1R	3	IkB = 1; IL1R = 0
IkB_MyD88	3	IkB = 1; MyD88 = 0
IkB_IRAK	3	IkB = 1; IRAK = 0

groupcombination	valuecombination	hypothesis
IkB_TRAF6	3	IkB = 1; TRAF6 = 0
IkB_TAB	3	IkB = 1; TAB = 0
IkB_TAK1	3	IkB = 1; TAK1 = 0
IkB_MEKK	3	IkB = 1; MEKK = 0
IkB_MKK	3	IkB = 1; MKK = 0
IkB_JNK	3	IkB = 1; JNK = 0
IkB_p38	3	IkB = 1; p38 = 0
IkB_cJun	3	IkB = 1; cJun = 0
IkB_cJun	4	IkB = 1; cJun = 1
IkB_cFos	3	IkB = 1; cFos = 0
IkB_cFos	4	IkB = 1; cFos = 1
IkB_AP1	3	IkB = 1; AP1 = 0
IkB_MKP1	3	IkB = 1; MKP1 = 0
IkB_MKP1	4	IkB = 1; MKP1 = 1
IkB_NIK	3	IkB = 1; NIK = 0
IkB_NIK	4	IkB = 1; NIK = 1
IkB_IL6R	3	IkB = 1; IL6R = 0
IkB_IL6R	4	IkB = 1; IL6R = 1
IkB_GP130	3	IkB = 1; GP130 = 0
IkB_GP130	4	IkB = 1; GP130 = 1
IkB_PI3K	3	IkB = 1; PI3K = 0
IkB_PI3K	4	IkB = 1; PI3K = 1
IkB_JAK	3	IkB = 1; JAK = 0
IkB_JAK	4	IkB = 1; JAK = 1
IkB_Akt	3	IkB = 1; Akt = 0
IkB_Akt	4	IkB = 1; Akt = 1
IkB_mTOR	3	IkB = 1; mTOR = 0
IkB_mTOR	4	IkB = 1; mTOR = 1
IkB_SOCS3	3	IkB = 1; SOCS3 = 0
IkB_SOCS3	4	IkB = 1; SOCS3 = 1
IkB_MEK1_2	3	IkB = 1; MEK1_2 = 0
IkB_MEK1_2	4	IkB = 1; MEK1_2 = 1
IkB_ERK1_2	3	IkB = 1; ERK1_2 = 0
IkB_Elk1	3	IkB = 1; Elk1 = 0
IkB_CEBPbeta	3	IkB = 1; CEBPbeta = 0
IkB_STAT3	3	IkB = 1; STAT3 = 0
IkB_STAT3	4	IkB = 1; STAT3 = 1
NFkB_IL1	1	NFkB = 0; IL1 = 0
NFkB_IL1R	1	NFkB = 0; IL1R = 0
NFkB_MyD88	1	NFkB = 0; MyD88 = 0
NFkB_IRAK	1	NFkB = 0; IRAK = 0
NFkB_TRAF6	1	NFkB = 0; TRAF6 = 0
NFkB_TAB	1	NFkB = 0; TAB = 0
NFkB_TAK1	1	NFkB = 0; TAK1 = 0
NFkB_MEKK	1	NFkB = 0; MEKK = 0
NFkB_MKK	1	NFkB = 0; MKK = 0
NFkB_JNK	1	NFkB = 0; JNK = 0
NFkB_p38	1	NFkB = 0; p38 = 0
NFkB_cJun	1	NFkB = 0; cJun = 0
NFkB_cJun	2	NFkB = 0; cJun = 1
NFkB_cFos	1	NFkB = 0; cFos = 0
NFkB_cFos	2	NFkB = 0; cFos = 1

groupcombination	valuecombination	hypothesis
NFkB__AP1	1	NFkB = 0; AP1 = 0
NFkB__MKP1	1	NFkB = 0; MKP1 = 0
NFkB__MKP1	2	NFkB = 0; MKP1 = 1
NFkB__NIK	1	NFkB = 0; NIK = 0
NFkB__NIK	2	NFkB = 0; NIK = 1
NFkB__IL6R	1	NFkB = 0; IL6R = 0
NFkB__IL6R	2	NFkB = 0; IL6R = 1
NFkB__GP130	1	NFkB = 0; GP130 = 0
NFkB__GP130	2	NFkB = 0; GP130 = 1
NFkB__PI3K	1	NFkB = 0; PI3K = 0
NFkB__PI3K	2	NFkB = 0; PI3K = 1
NFkB__JAK	1	NFkB = 0; JAK = 0
NFkB__JAK	2	NFkB = 0; JAK = 1
NFkB__Akt	1	NFkB = 0; Akt = 0
NFkB__Akt	2	NFkB = 0; Akt = 1
NFkB__mTOR	1	NFkB = 0; mTOR = 0
NFkB__mTOR	2	NFkB = 0; mTOR = 1
NFkB__SOCS3	1	NFkB = 0; SOCS3 = 0
NFkB__SOCS3	2	NFkB = 0; SOCS3 = 1
NFkB__MEK1__2	1	NFkB = 0; MEK1__2 = 0
NFkB__MEK1__2	2	NFkB = 0; MEK1__2 = 1
NFkB__ERK1__2	1	NFkB = 0; ERK1__2 = 0
NFkB__Elk1	1	NFkB = 0; Elk1 = 0
NFkB__CEBPbeta	1	NFkB = 0; CEBPbeta = 0
NFkB__STAT3	1	NFkB = 0; STAT3 = 0
NFkB__STAT3	2	NFkB = 0; STAT3 = 1

#Classification of all single node perturbations in SASP network

We analyzed all single-node perturbations in the `SASP`-network and compared the results to the attractors of the original network. All single-node perturbations including knockout and overexpression perturbations, were classified by their effects on the different attractors of the `SASP`-network. Some of the different perturbation experiments have the same effects on the network. We classified the pertubations by the attractor ids in the wild-type network which they effect.

```
genes <- saspnetwork$genes
attractors <- getAttractors(saspnetwork, method = "sat.exhaustive")
#plot attractors groupwise to prevent cutting of a figure in PDF-file
par(mar=c(5,7,5,4))
p <- plotAttractors(attractors,
                    title = "All attractors of the SASP Boolean network model
                             using synchronous updates",
                    offColor = "#7b3294", onColor = "#a6dba0")
```

All attractors of the SASP Boolean network model using synchronous updates
Attractors with 1 state(s)

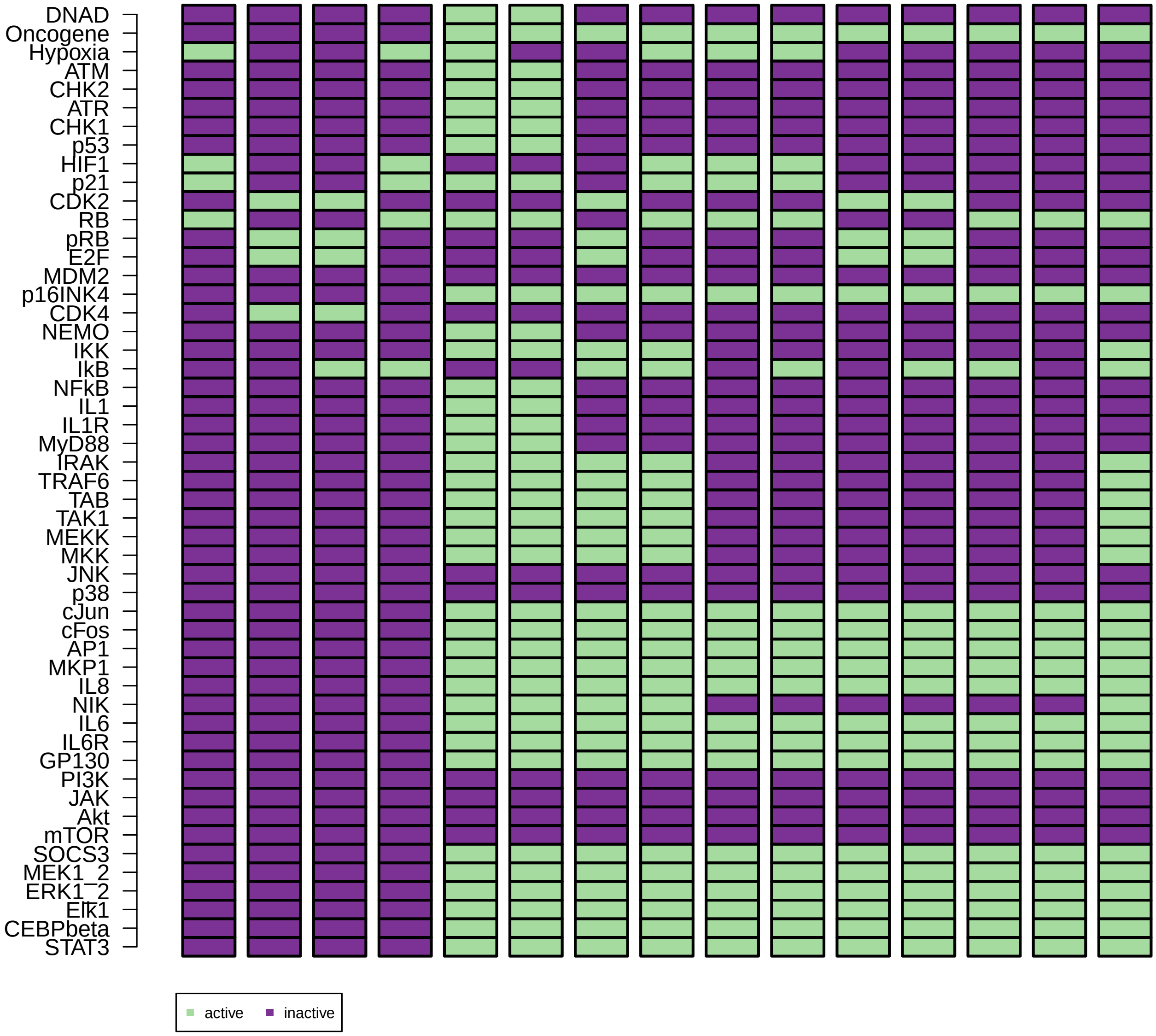

All attractors of the SASP Boolean network model using synchronous updates
Attractors with 3 state(s)

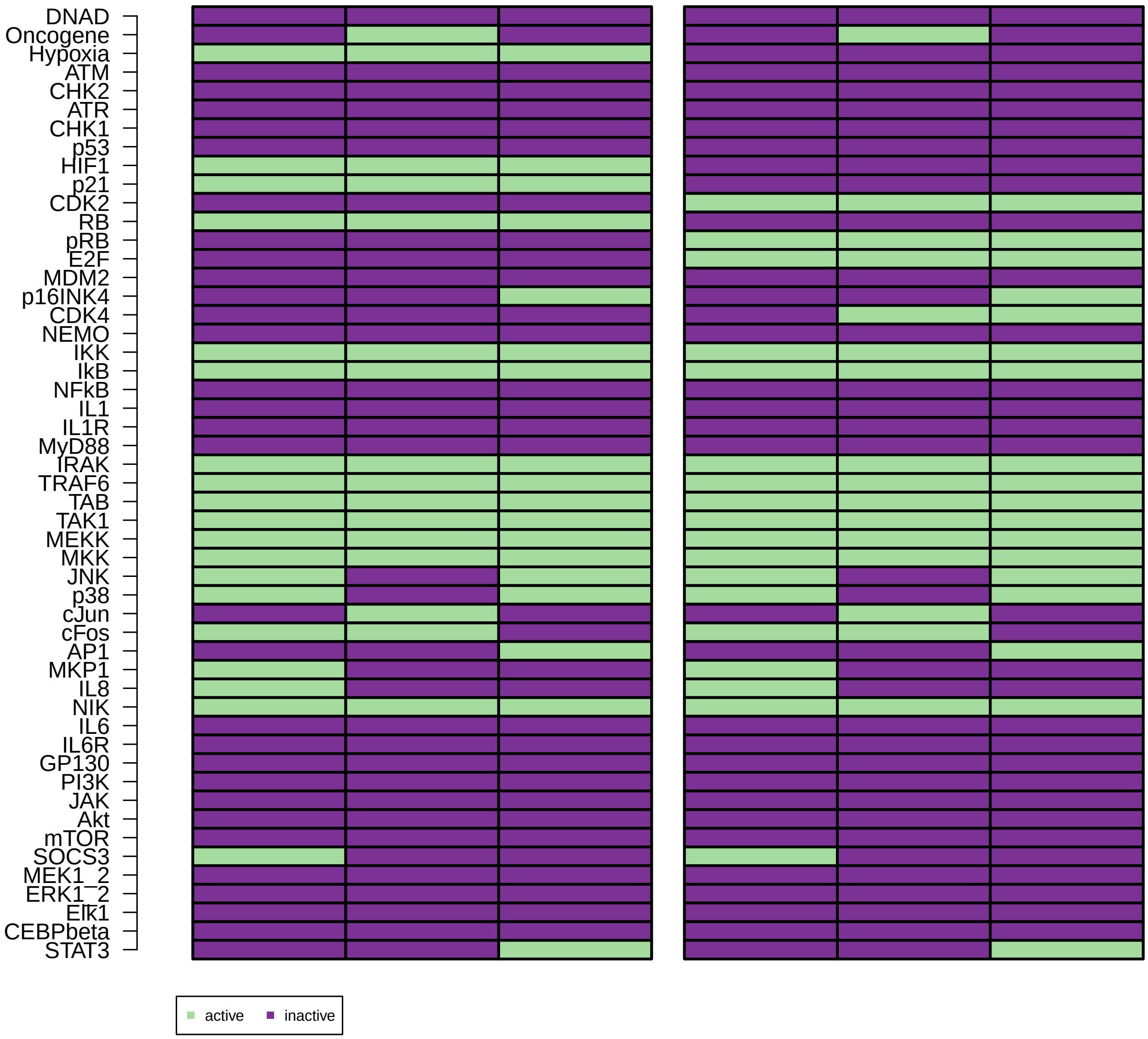

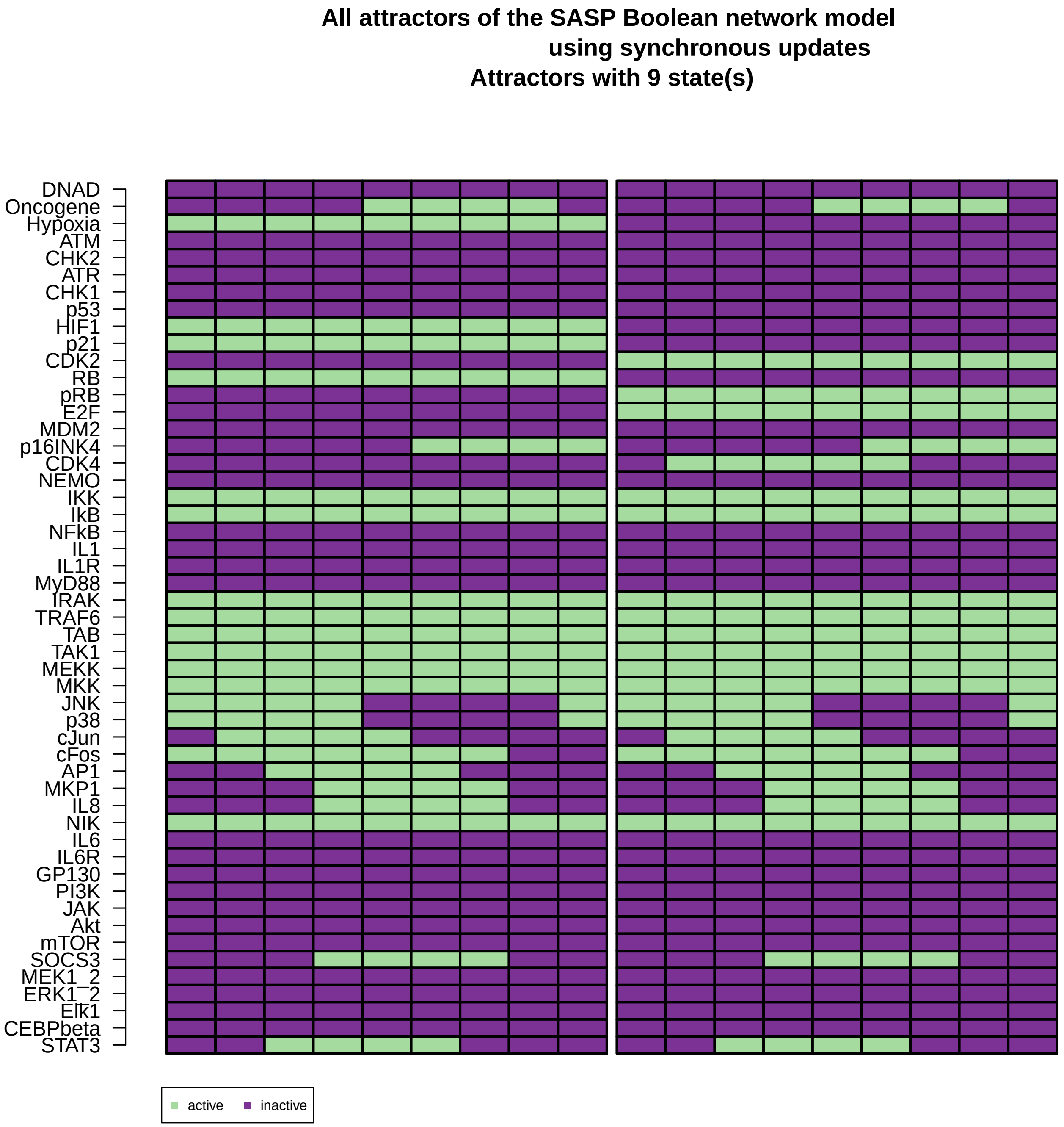


```
#generate all kO/overexp combinations of depth 1
combs <- combn(unlist(lapply(genes, function(x) list(c(x,0), c(x,1))),
                      recursive=FALSE), 1)

combsNaming <- combn(unlist(lapply(genes, function(x)
  list(paste(x, "_kO", sep =""), paste(x,"_OE",sep = ""))),
  recursive=FALSE),
  1)

#do attractor search on each perturbed network
modAttrs <- apply(combs, 2, function(comb)
{
```

```
  geneNames <- sapply(comb, function(x)x[[1]])
  geneVals <- sapply(comb, function(x)as.integer(x[[2]]))

  net2 <- fixGenes(saspnetwork,
                   geneNames,
                   geneVals)

  a <- getAttractors(net2, method = "sat.exhaustive")
  return(a)
})

#classify perturbations by changes in attractors
equalAttractorIDs <- lapply(modAttrs, getIDofEqualAttractors, origAttrs = attractors, partly = F)
missingAttrs <- lapply(equalAttractorIDs, setdiff, x = 1:length(attractors$attractors))
names(equalAttractorIDs) <- names(missingAttrs) <- unlist(combsNaming[1,])
equalTable <- Reduce(cbind,
                     lapply(equalAttractorIDs,
                            function(v) 1:length(attractors$attractors) %in% v))
colnames(equalTable) <- combsNaming[1,]
rownames(equalTable) <- paste0("Attr ", 1:length(attractors$attractors))
equalTable <- data.frame(t(equalTable))

#function to count groups
count.duplicates <- function(DF){
x <- do.call('paste', c(DF, sep = '\r'))
  ox <- order(x)
  rl <- rle(x[ox])
  cbind(DF[ox[cumsum(rl$lengths)],,drop=FALSE],freq = rl$lengths)
}

#group perturbations leading to changes in same attractors
groups <- count.duplicates(equalTable)
rownames(groups) <- paste0("Group ", 1:nrow(groups))
groups[groups == T] <- 1
groups[groups == F] <- 0

#group perturbations by attractor (e.g. attractor1:G1Ko,G2OE,..., attractor2:...)
groupedByAttractor <- apply(equalTable, MARGIN = 2, function(e) {
  rownames(equalTable)[which(e == F)]
})

groupedByAttractor
```

```
$Attr.1
 [1] "DNAD_OE"     "Oncogene_OE" "Hypoxia_kO"  "ATM_OE"      "CHK2_OE"
 [6] "ATR_OE"      "CHK1_OE"     "p53_OE"      "HIF1_kO"     "p21_kO"
[11] "CDK2_OE"     "pRB_OE"      "CDK4_OE"     "NEMO_OE"     "IKK_OE"
[16] "NFkB_OE"     "IL1_OE"      "IL1R_OE"     "MyD88_OE"    "IRAK_OE"
[21] "TRAF6_OE"    "TAB_OE"      "TAK1_OE"     "MEKK_OE"     "MKK_OE"
[26] "JNK_OE"      "p38_OE"      "AP1_OE"      "IL8_OE"      "NIK_OE"
[31] "IL6_OE"      "IL6R_OE"     "PI3K_OE"     "JAK_OE"      "Akt_OE"
[36] "mTOR_OE"     "ERK1_2_OE"   "Elk1_OE"     "CEBPbeta_OE" "STAT3_OE"

$Attr.2
```

```
 [1] "DNAD_OE"     "Oncogene_OE" "Hypoxia_OE"  "ATM_OE"      "CHK2_OE"
 [6] "ATR_OE"      "CHK1_OE"     "p53_OE"      "HIF1_OE"     "p21_OE"
[11] "RB_OE"       "E2F_kO"      "p16INK4_OE"  "NEMO_OE"     "IKK_OE"
[16] "NFkB_OE"     "IL1_OE"      "IL1R_OE"     "MyD88_OE"    "IRAK_OE"
[21] "TRAF6_OE"    "TAB_OE"      "TAK1_OE"     "MEKK_OE"     "MKK_OE"
[26] "JNK_OE"      "p38_OE"      "AP1_OE"      "IL8_OE"      "NIK_OE"
[31] "IL6_OE"      "IL6R_OE"     "PI3K_OE"     "JAK_OE"      "Akt_OE"
[36] "mTOR_OE"     "ERK1_2_OE"   "Elk1_OE"     "CEBPbeta_OE" "STAT3_OE"

$Attr.3
 [1] "DNAD_OE"     "Oncogene_OE" "Hypoxia_OE"  "ATM_OE"      "CHK2_OE"
 [6] "ATR_OE"      "CHK1_OE"     "p53_OE"      "HIF1_OE"     "p21_OE"
[11] "RB_OE"       "E2F_kO"      "p16INK4_OE"  "NEMO_OE"     "NFkB_OE"
[16] "IL1_OE"      "IL1R_OE"     "MyD88_OE"    "IRAK_OE"     "TRAF6_OE"
[21] "TAB_OE"      "TAK1_OE"     "MEKK_OE"     "MKK_OE"      "JNK_OE"
[26] "p38_OE"      "AP1_OE"      "IL8_OE"      "NIK_OE"      "IL6_OE"
[31] "PI3K_OE"     "JAK_OE"      "Akt_OE"      "mTOR_OE"     "ERK1_2_OE"
[36] "Elk1_OE"     "CEBPbeta_OE" "STAT3_OE"

$Attr.4
 [1] "DNAD_OE"     "Oncogene_OE" "Hypoxia_kO"  "ATM_OE"      "CHK2_OE"
 [6] "ATR_OE"      "CHK1_OE"     "p53_OE"      "HIF1_kO"     "p21_kO"
[11] "CDK2_OE"     "pRB_OE"      "CDK4_OE"     "NEMO_OE"     "NFkB_OE"
[16] "IL1_OE"      "IL1R_OE"     "MyD88_OE"    "IRAK_OE"     "TRAF6_OE"
[21] "TAB_OE"      "TAK1_OE"     "MEKK_OE"     "MKK_OE"      "JNK_OE"
[26] "p38_OE"      "AP1_OE"      "IL8_OE"      "NIK_OE"      "IL6_OE"
[31] "PI3K_OE"     "JAK_OE"      "Akt_OE"      "mTOR_OE"     "ERK1_2_OE"
[36] "Elk1_OE"     "CEBPbeta_OE" "STAT3_OE"

$Attr.5
 [1] "DNAD_kO"   "ATM_kO"    "ATR_kO"    "p53_kO"    "CDK2_OE"
 [6] "pRB_OE"    "MDM2_OE"   "CDK4_OE"   "NEMO_kO"   "IKK_kO"
[11] "IkB_OE"    "NFkB_kO"   "IL1_kO"    "IL1R_kO"   "IRAK_kO"
[16] "TRAF6_kO"  "TAK1_kO"   "cJun_kO"   "cFos_kO"   "AP1_kO"
[21] "MKP1_kO"   "IL6_kO"    "GP130_kO"  "PI3K_OE"   "JAK_OE"
[26] "Akt_OE"    "SOCS3_kO"  "MEK1_2_kO" "ERK1_2_kO" "Elk1_kO"
[31] "STAT3_kO"

$Attr.6
 [1] "DNAD_kO"   "ATM_kO"    "ATR_kO"    "p53_kO"    "CDK2_OE"
 [6] "pRB_OE"    "MDM2_OE"   "CDK4_OE"   "NEMO_kO"   "IKK_kO"
[11] "IkB_OE"    "NFkB_kO"   "IL1_kO"    "IL1R_kO"   "IRAK_kO"
[16] "TRAF6_kO"  "TAK1_kO"   "cJun_kO"   "cFos_kO"   "AP1_kO"
[21] "MKP1_kO"   "IL6_kO"    "GP130_kO"  "PI3K_OE"   "JAK_OE"
[26] "Akt_OE"    "SOCS3_kO"  "MEK1_2_kO" "ERK1_2_kO" "Elk1_kO"
[31] "STAT3_kO"

$Attr.7
 [1] "DNAD_OE"     "Oncogene_kO" "Hypoxia_OE"  "ATM_OE"      "CHK2_OE"
 [6] "ATR_OE"      "CHK1_OE"     "p53_OE"      "HIF1_OE"     "p21_OE"
[11] "CDK2_kO"     "RB_OE"       "E2F_kO"      "p16INK4_kO"  "NEMO_OE"
[16] "IkB_kO"      "NFkB_OE"     "IL1_OE"      "IL1R_OE"     "IRAK_kO"
[21] "TRAF6_kO"    "TAK1_kO"     "cJun_kO"     "cFos_kO"     "AP1_kO"
[26] "MKP1_kO"     "NIK_kO"      "IL6_kO"      "GP130_kO"    "PI3K_OE"
```

```
[31] "JAK_OE"       "Akt_OE"       "SOCS3_kO"     "MEK1_2_kO"    "ERK1_2_kO"
[36] "Elk1_kO"      "STAT3_kO"

$Attr.8
 [1] "DNAD_OE"      "Oncogene_kO" "Hypoxia_kO"   "ATM_OE"       "CHK2_OE"
 [6] "ATR_OE"       "CHK1_OE"      "p53_OE"       "HIF1_kO"      "CDK2_OE"
[11] "pRB_OE"       "CDK4_OE"      "NEMO_OE"      "IkB_kO"       "NFkB_OE"
[16] "IL1_OE"       "IL1R_OE"      "IRAK_kO"      "TRAF6_kO"     "TAK1_kO"
[21] "cJun_kO"      "cFos_kO"      "AP1_kO"       "MKP1_kO"      "NIK_kO"
[26] "IL6_kO"       "GP130_kO"     "PI3K_OE"      "JAK_OE"       "Akt_OE"
[31] "SOCS3_kO"     "MEK1_2_kO"    "ERK1_2_kO"    "Elk1_kO"      "STAT3_kO"

$Attr.9
 [1] "DNAD_OE"      "Oncogene_kO" "Hypoxia_kO"   "ATM_OE"       "CHK2_OE"
 [6] "ATR_OE"       "CHK1_OE"      "p53_OE"       "HIF1_kO"      "CDK2_OE"
[11] "pRB_OE"       "CDK4_OE"      "NEMO_OE"      "IKK_OE"       "NFkB_OE"
[16] "IL1_OE"       "IL1R_OE"      "MyD88_OE"     "IRAK_OE"      "TRAF6_OE"
[21] "TAB_OE"       "TAK1_OE"      "MEKK_OE"      "cJun_kO"      "cFos_kO"
[26] "AP1_kO"       "NIK_OE"       "IL6_kO"       "GP130_kO"     "PI3K_OE"
[31] "JAK_OE"       "Akt_OE"       "SOCS3_kO"     "MEK1_2_kO"    "ERK1_2_kO"
[36] "Elk1_kO"      "STAT3_kO"

$Attr.10
 [1] "DNAD_OE"      "Oncogene_kO" "Hypoxia_kO"   "ATM_OE"       "CHK2_OE"
 [6] "ATR_OE"       "CHK1_OE"      "p53_OE"       "HIF1_kO"      "CDK2_OE"
[11] "pRB_OE"       "CDK4_OE"      "NEMO_OE"      "NFkB_OE"      "IL1_OE"
[16] "IL1R_OE"      "MyD88_OE"     "IRAK_OE"      "TRAF6_OE"     "TAB_OE"
[21] "TAK1_OE"      "MEKK_OE"      "cJun_kO"      "cFos_kO"      "AP1_kO"
[26] "NIK_OE"       "IL6_kO"       "GP130_kO"     "PI3K_OE"      "JAK_OE"
[31] "Akt_OE"       "SOCS3_kO"     "MEK1_2_kO"    "ERK1_2_kO"    "Elk1_kO"
[36] "STAT3_kO"

$Attr.11
 [1] "DNAD_OE"      "Oncogene_kO" "Hypoxia_OE"   "ATM_OE"       "CHK2_OE"
 [6] "ATR_OE"       "CHK1_OE"      "p53_OE"       "HIF1_OE"      "p21_OE"
[11] "CDK2_kO"      "RB_OE"        "E2F_kO"       "p16INK4_kO"   "NEMO_OE"
[16] "IKK_OE"       "NFkB_OE"      "IL1_OE"       "IL1R_OE"      "MyD88_OE"
[21] "IRAK_OE"      "TRAF6_OE"     "TAB_OE"       "TAK1_OE"      "MEKK_OE"
[26] "cJun_kO"      "cFos_kO"      "AP1_kO"       "NIK_OE"       "IL6_kO"
[31] "GP130_kO"     "PI3K_OE"      "JAK_OE"       "Akt_OE"       "SOCS3_kO"
[36] "MEK1_2_kO"    "ERK1_2_kO"    "Elk1_kO"      "STAT3_kO"

$Attr.12
 [1] "DNAD_OE"      "Oncogene_kO" "Hypoxia_OE"   "ATM_OE"       "CHK2_OE"
 [6] "ATR_OE"       "CHK1_OE"      "p53_OE"       "HIF1_OE"      "p21_OE"
[11] "CDK2_kO"      "RB_OE"        "E2F_kO"       "p16INK4_kO"   "NEMO_OE"
[16] "NFkB_OE"      "IL1_OE"       "IL1R_OE"      "MyD88_OE"     "IRAK_OE"
[21] "TRAF6_OE"     "TAB_OE"       "TAK1_OE"      "MEKK_OE"      "cJun_kO"
[26] "cFos_kO"      "AP1_kO"       "NIK_OE"       "IL6_kO"       "GP130_kO"
[31] "PI3K_OE"      "JAK_OE"       "Akt_OE"       "SOCS3_kO"     "MEK1_2_kO"
[36] "ERK1_2_kO"    "Elk1_kO"      "STAT3_kO"

$Attr.13
 [1] "DNAD_OE"      "Oncogene_kO" "Hypoxia_OE"   "ATM_OE"       "ATR_OE"
```

```
 [6] "p53_OE"       "HIF1_OE"      "CDK2_OE"      "pRB_OE"       "E2F_OE"
[11] "p16INK4_kO"   "CDK4_OE"      "NEMO_OE"      "NFkB_OE"      "IL1_OE"
[16] "IL1R_OE"      "MyD88_OE"     "IRAK_OE"      "TRAF6_OE"     "TAB_OE"
[21] "TAK1_OE"      "MEKK_OE"      "cJun_kO"      "cFos_kO"      "AP1_kO"
[26] "NIK_OE"       "IL6_kO"       "GP130_kO"     "PI3K_OE"      "JAK_OE"
[31] "Akt_OE"       "SOCS3_kO"     "MEK1_2_kO"    "ERK1_2_kO"    "Elk1_kO"
[36] "STAT3_kO"

$Attr.14
 [1] "DNAD_OE"      "Oncogene_kO" "Hypoxia_OE"   "ATM_OE"       "ATR_OE"
 [6] "p53_OE"       "HIF1_OE"      "CDK2_OE"      "pRB_OE"       "E2F_OE"
[11] "p16INK4_kO"   "CDK4_OE"      "NEMO_OE"      "IKK_OE"       "NFkB_OE"
[16] "IL1_OE"       "IL1R_OE"      "MyD88_OE"     "IRAK_OE"      "TRAF6_OE"
[21] "TAB_OE"       "TAK1_OE"      "MEKK_OE"      "cJun_kO"      "cFos_kO"
[26] "AP1_kO"       "NIK_OE"       "IL6_kO"       "GP130_kO"     "PI3K_OE"
[31] "JAK_OE"       "Akt_OE"       "SOCS3_kO"     "MEK1_2_kO"    "ERK1_2_kO"
[36] "Elk1_kO"      "STAT3_kO"

$Attr.15
 [1] "DNAD_OE"      "Oncogene_kO" "Hypoxia_OE"   "ATM_OE"       "ATR_OE"
 [6] "p53_OE"       "HIF1_OE"      "CDK2_OE"      "pRB_OE"       "E2F_OE"
[11] "p16INK4_kO"   "CDK4_OE"      "NEMO_OE"      "IkB_kO"       "NFkB_OE"
[16] "IL1_OE"       "IL1R_OE"      "IRAK_kO"      "TRAF6_kO"     "TAK1_kO"
[21] "cJun_kO"      "cFos_kO"      "AP1_kO"       "MKP1_kO"      "NIK_kO"
[26] "IL6_kO"       "GP130_kO"     "PI3K_OE"      "JAK_OE"       "Akt_OE"
[31] "SOCS3_kO"     "MEK1_2_kO"    "ERK1_2_kO"    "Elk1_kO"      "STAT3_kO"

$Attr.16
 [1] "DNAD_OE"      "Oncogene_kO" "Oncogene_OE" "Hypoxia_kO"   "ATM_OE"
 [6] "CHK2_OE"      "ATR_OE"       "CHK1_OE"      "p53_OE"       "HIF1_kO"
[11] "p21_kO"       "CDK2_OE"      "pRB_OE"       "CDK4_OE"      "NEMO_OE"
[16] "IkB_kO"       "NFkB_OE"      "IL1_OE"       "IL1R_OE"      "IRAK_kO"
[21] "TRAF6_kO"     "TAK1_kO"      "MKK_kO"       "JNK_OE"       "p38_OE"
[26] "cJun_kO"      "cJun_OE"      "cFos_kO"      "cFos_OE"      "AP1_kO"
[31] "AP1_OE"       "MKP1_kO"      "MKP1_OE"      "IL8_kO"       "IL8_OE"
[36] "NIK_kO"       "IL6_OE"       "IL6R_OE"      "PI3K_OE"      "JAK_OE"
[41] "Akt_OE"       "mTOR_OE"      "ERK1_2_OE"    "Elk1_OE"      "CEBPbeta_OE"
[46] "STAT3_kO"     "STAT3_OE"

$Attr.17
 [1] "DNAD_OE"      "Oncogene_kO" "Oncogene_OE" "Hypoxia_OE"   "ATM_OE"
 [6] "CHK2_OE"      "ATR_OE"       "CHK1_OE"      "p53_OE"       "HIF1_OE"
[11] "p21_OE"       "CDK2_kO"      "RB_OE"        "E2F_kO"       "p16INK4_kO"
[16] "p16INK4_OE"   "NEMO_OE"      "IkB_kO"       "NFkB_OE"      "IL1_OE"
[21] "IL1R_OE"      "IRAK_kO"      "TRAF6_kO"     "TAK1_kO"      "MKK_kO"
[26] "JNK_OE"       "p38_OE"       "cJun_kO"      "cJun_OE"      "cFos_kO"
[31] "cFos_OE"      "AP1_kO"       "AP1_OE"       "MKP1_kO"      "MKP1_OE"
[36] "IL8_kO"       "IL8_OE"       "NIK_kO"       "IL6_OE"       "IL6R_OE"
[41] "PI3K_OE"      "JAK_OE"       "Akt_OE"       "mTOR_OE"      "ERK1_2_OE"
[46] "Elk1_OE"      "CEBPbeta_OE" "STAT3_kO"     "STAT3_OE"

$Attr.18
 [1] "DNAD_OE"      "Oncogene_kO" "Oncogene_OE" "Hypoxia_kO"   "ATM_OE"
 [6] "CHK2_OE"      "ATR_OE"       "CHK1_OE"      "p53_OE"       "HIF1_kO"
```

```
[11] "p21_kO"      "CDK2_OE"     "pRB_OE"      "CDK4_OE"     "NEMO_OE"
[16] "IkB_kO"      "NFkB_OE"     "IL1_OE"      "IL1R_OE"     "IRAK_kO"
[21] "TRAF6_kO"    "TAK1_kO"     "MKK_kO"      "JNK_OE"      "p38_OE"
[26] "cJun_kO"     "cJun_OE"     "cFos_kO"     "cFos_OE"     "AP1_kO"
[31] "AP1_OE"      "MKP1_kO"     "MKP1_OE"     "IL8_kO"      "IL8_OE"
[36] "NIK_kO"      "IL6_OE"      "IL6R_OE"     "PI3K_OE"     "JAK_OE"
[41] "Akt_OE"      "mTOR_OE"     "ERK1_2_OE"   "Elk1_OE"     "CEBPbeta_OE"
[46] "STAT3_kO"    "STAT3_OE"

$Attr.19
 [1] "DNAD_OE"     "Oncogene_kO" "Oncogene_OE" "Hypoxia_OE"  "ATM_OE"
 [6] "CHK2_OE"     "ATR_OE"      "CHK1_OE"     "p53_OE"      "HIF1_OE"
[11] "p21_OE"      "CDK2_kO"     "RB_OE"       "E2F_kO"      "p16INK4_kO"
[16] "p16INK4_OE"  "NEMO_OE"     "IkB_kO"      "NFkB_OE"     "IL1_OE"
[21] "IL1R_OE"     "IRAK_kO"     "TRAF6_kO"    "TAK1_kO"     "MKK_kO"
[26] "JNK_OE"      "p38_OE"      "cJun_kO"     "cJun_OE"     "cFos_kO"
[31] "cFos_OE"     "AP1_kO"      "AP1_OE"      "MKP1_kO"     "MKP1_OE"
[36] "IL8_kO"      "IL8_OE"      "NIK_kO"      "IL6_OE"      "IL6R_OE"
[41] "PI3K_OE"     "JAK_OE"      "Akt_OE"      "mTOR_OE"     "ERK1_2_OE"
[46] "Elk1_OE"     "CEBPbeta_OE" "STAT3_kO"    "STAT3_OE"
```

Focusing on the attractors 5 and 6, which represent the DNA damage response, the classification shows the same results. Both attractors are removed by the same perturbations. On one hand, this is another indication for the similarity of these two attractors. On the other hand, it can be seen, that the relevant perturbations disturb the DNA damage dependent activation of the proinflammatory parts of the network (including the perturbations of NEMO, Iκb and NF-κB as tested in the manuscript).

In-vitro validation of model predictions

To validate the prediction of the proposed `SASP` model, the hypothesis from the in-silico experiments are compared to in-vitro experiments. The data is either published in publications we did not use for model creation or performed for this manuscript.

In-silico knockout of `NEMO` predicts a set of genes is not changed due to the knockout. To validate these results, we measured the expression level of the same genes under wild type conditions and `NEMO` knock out in-vitro (Supplementary Figure 2). Comparison of control and `NEMO` knockout results show that the measured genes remain stable under consideration of a two-fold cutoff. These results go hand in hand with the in-silico predictions of this experiment. For measured genes the model shows a prediction accuracy of 100% compared to the in-vitro experiments.

We further compared the predictions of the model behavior under DNA damage conditions (Fig 3) with the `SASP` marker panel in Coppé et al., 2008 [4]. A set of genes (`p53`, `p21`, `IL-1`, `IL-6`, `IL-8`) which the proposed model has in common with the measured genes by Coppé et al are compared to validate the model prediction. In Coppé et al.[4] the expression of these genes is measured under wild type and senescence conditions. Again considering a two-fold cutoff, in Coppé et al., the expression of all genes in the set is significantly increased. We correlate this with the predictions of the Boolean network. The Boolean networks simulation predicts a upgregulation of all genes in the set as well.

Rodier et al., 2009 [9] state that the `SASP` markers `IL-6` and `IL-8` as well as `CHK2` are increased after DNA damage. In their analysis they include in-vitro experiments with `ATM` knockout. Here, it was shown that knockout of `ATM` prevents the activation of `IL-6`, `IL-8`, `CHK2`. To validate our model, we inserted the knockout of `ATM` under DMA damage conditions.

```r
par(mar=c(5,7,5,4))
#initial state under DNA damage conditions
```

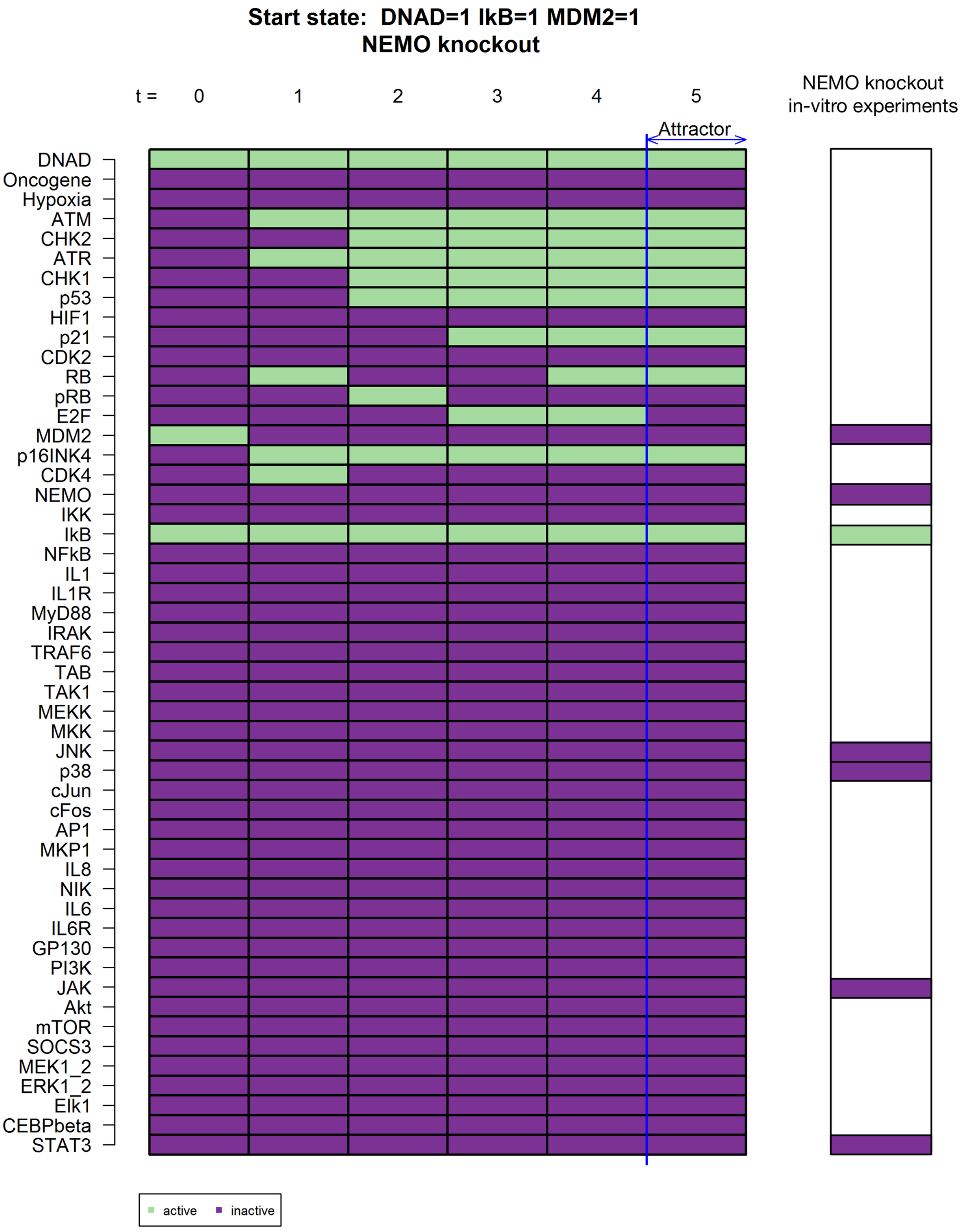

Figure 1: Comparison of in-silico knockout prediction of NEMO and in-vitro data for NEMO knockout

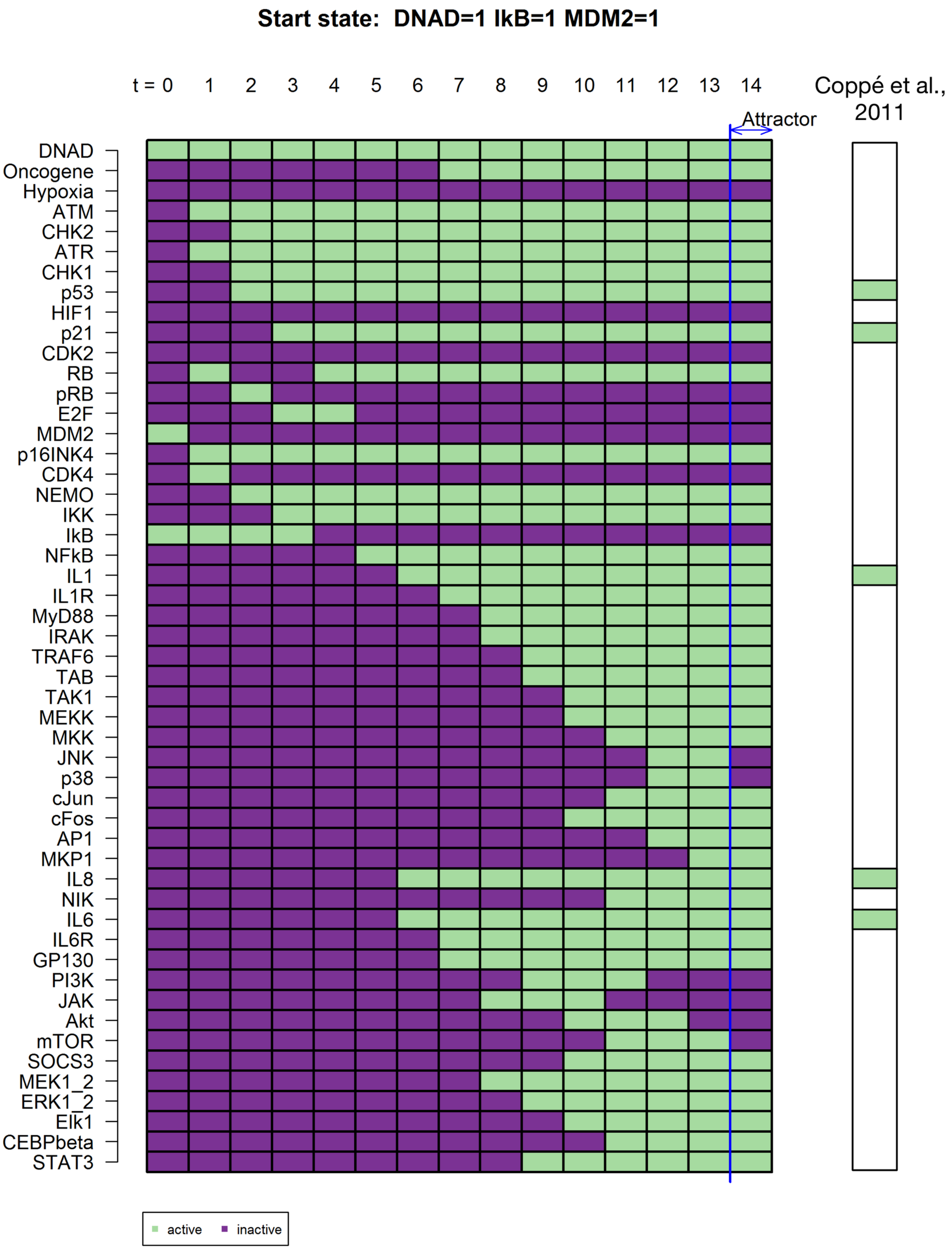

Figure 2: Comparison of in-silico DNA damage prediction and in-vitro data by Coppé et al., 2008

```r
startStates <- list(c(c(1,0,0,0,0,0,0,0,0,0,0,0,0,0,1,0,0,0,0,1),rep(0,31)))

#ATM knockout network
atmKO <- fixGenes(saspnetwork, "ATM", 0)

#attractor search
atmKOattrs <- getAttractors(atmKO,startStates = startStates)
par(mar=c(5,7,5,4))
#plotting attractor
p <- plotAttractors(atmKOattrs,
                    title = "Startstate : DNAD = 1, IkB = 1, MDM2 = 1, ATM knockout",
                    offColor = "#7b3294", onColor = "#a6dba0")
```

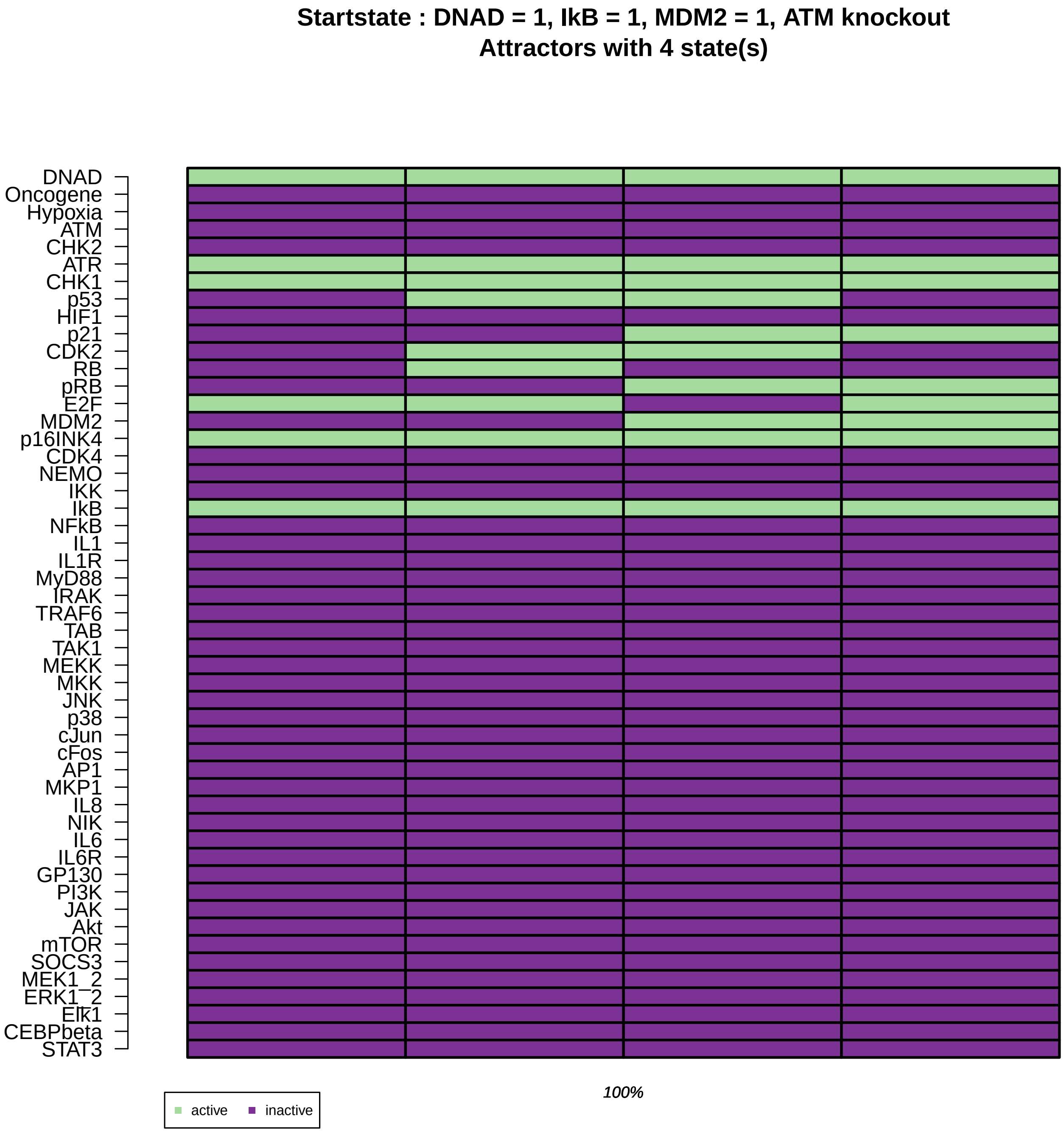

The figure shows a downregulation of `IL-6`, `IL-8` and `CHK2` after `ATM` knockout under DNA damage conditions as stated by Rodier et al., 2009.

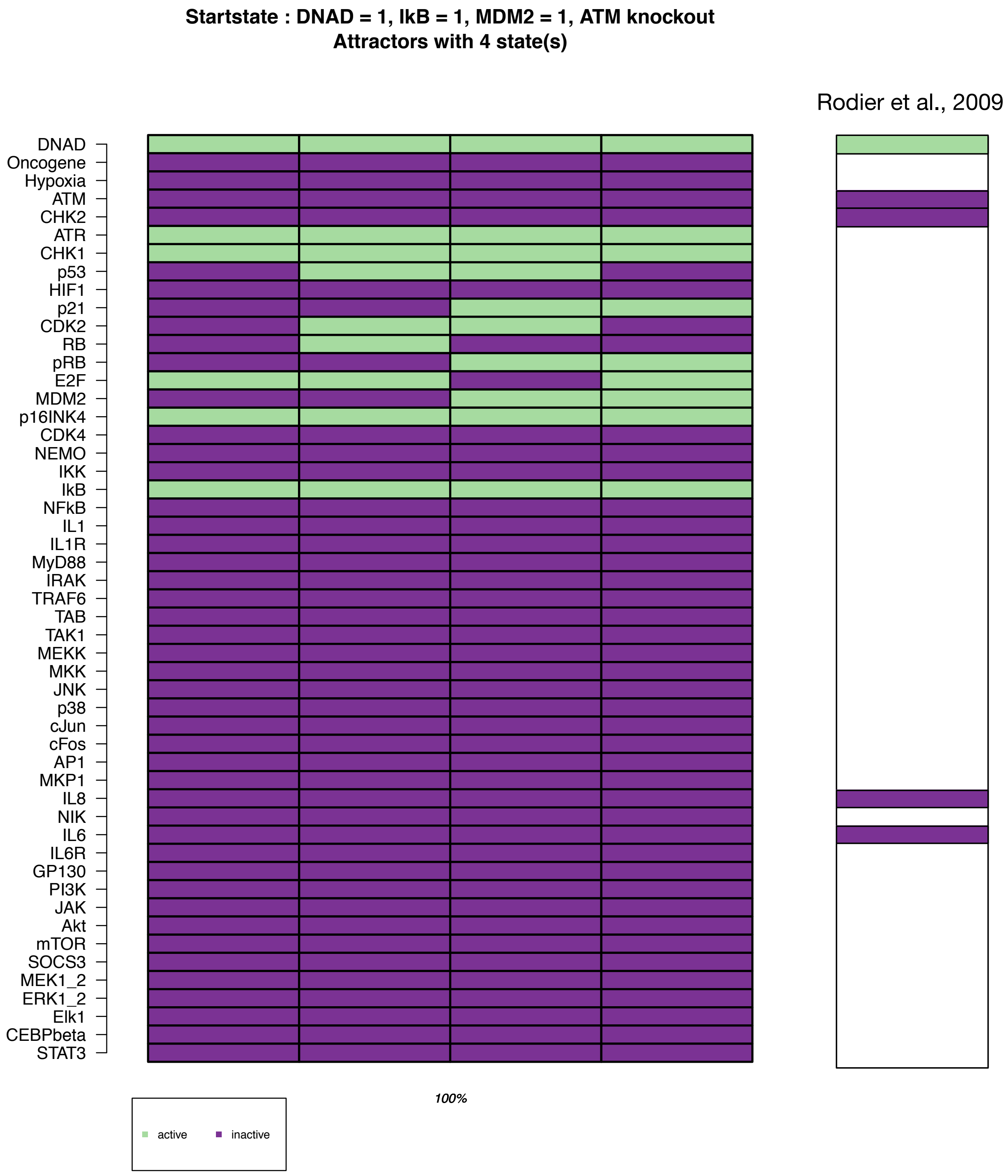

Figure 3: Comparison of in-silico DNA damage prediction with ATM knockout and in-vitro data by Rodier et al., 2009

Exhaustive attractor search

An exhaustive attractor search (using the `sat.exhaustive` option in BoolNet) reveals 19 different attractors.

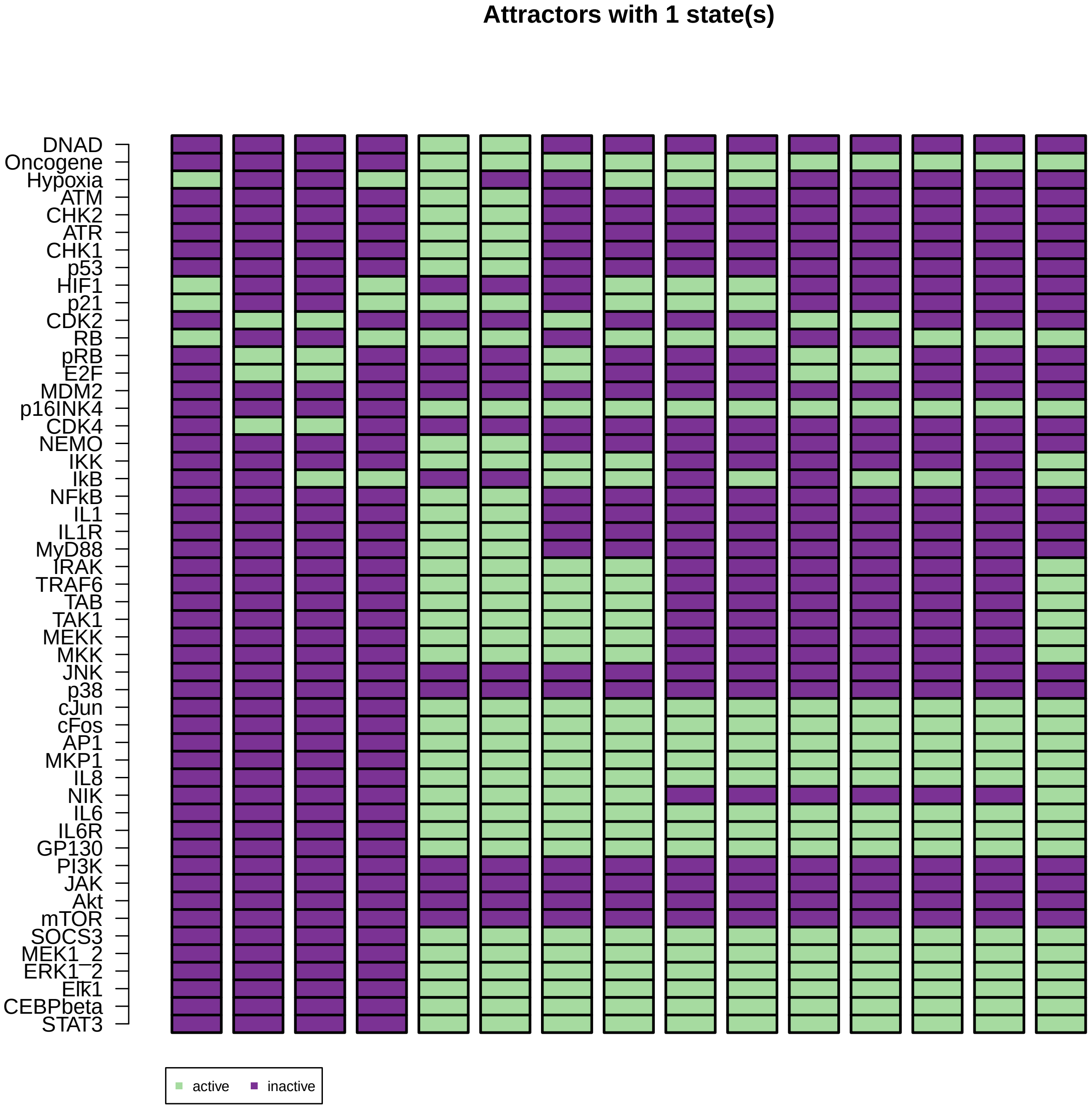

Attractors with 3 state(s)

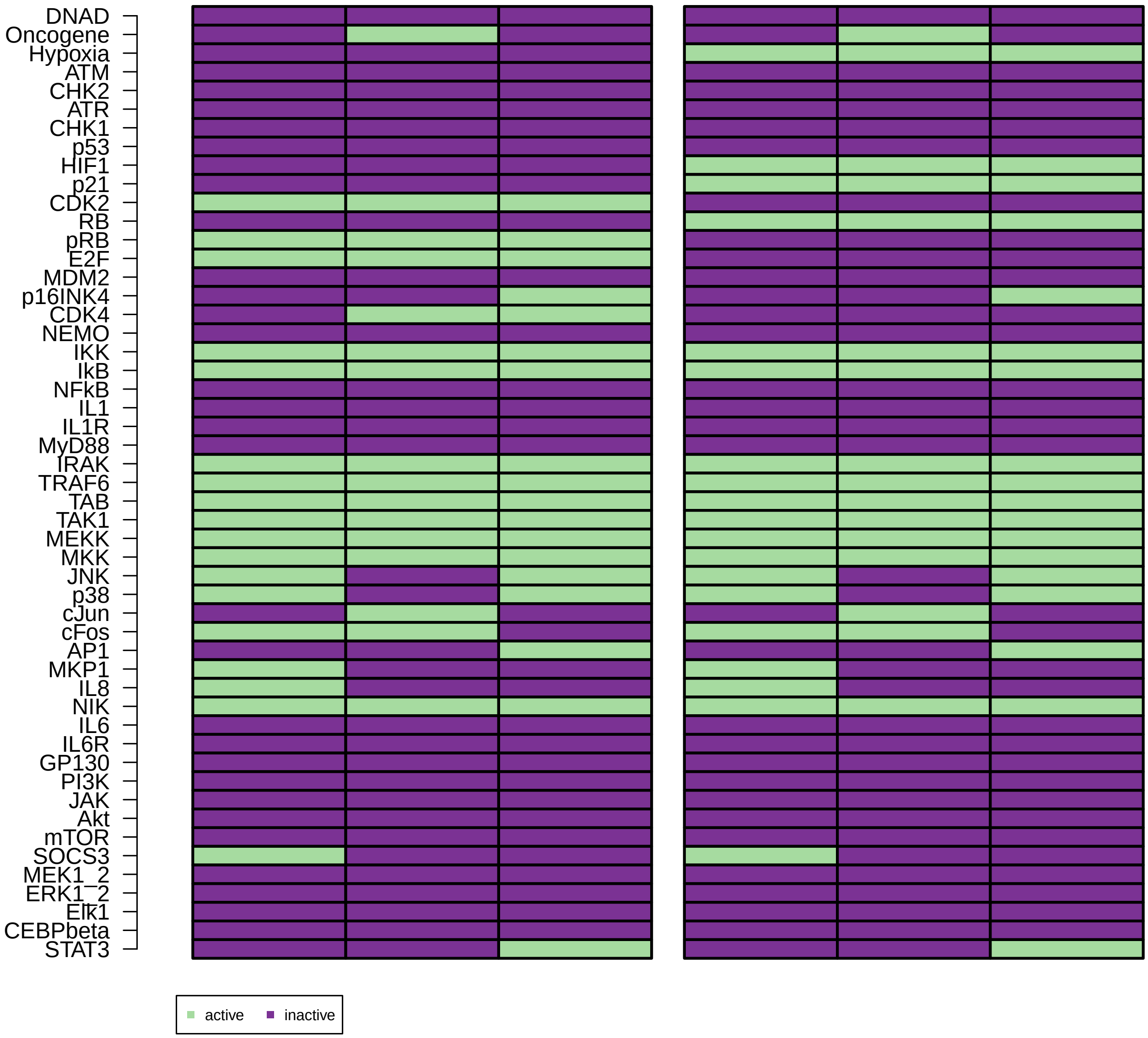

Attractors with 9 state(s)

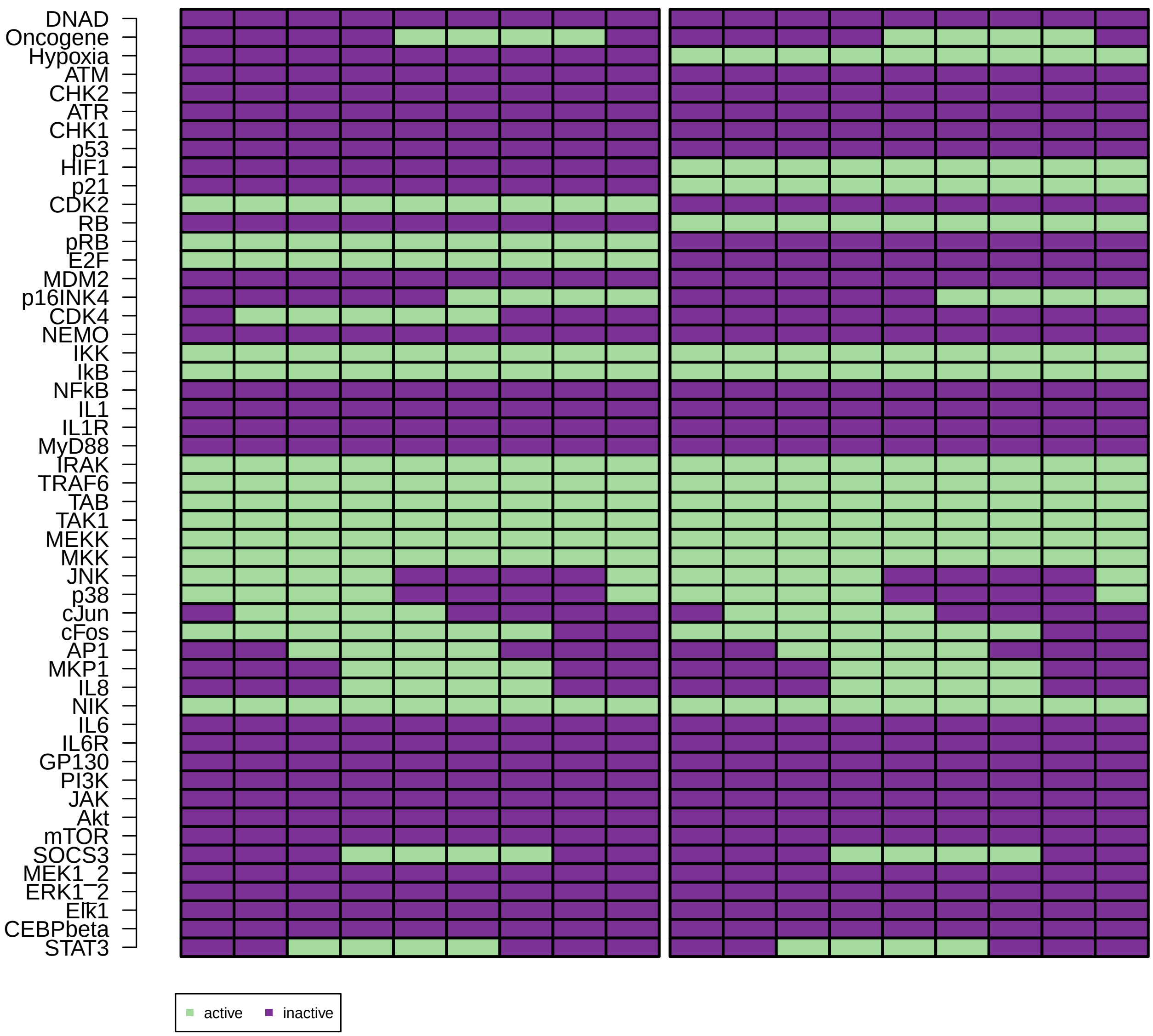

Unfortunately, an exhaustive search that also includes a direct computation of the basin size is not possible due to the sheer amount of 2^{51} combinations that needed to be evaluated. However, we can approximate the basin size by sampling `attrs <- getAttractors(saspnetwork, startStates = 50000000)`. It is worth noting that due to random sampling it will be impossible to capture and estimate basin sizes of all possible attractors. As can be seen in the Figure, only eight attractors take up the majority of the tested random states. All other attractors have a relatively low basin size which means that observation of such phenotypes (if the model would be perfect) can be considered very rare. Furthermore, these attractors come from start states that are biologically implausible.

In the single state attractors, four are taking up 70% of the basin based on the test random states. Two of the attractors with the largest basin size (column 2 and 3) correspond to the DNA damage response phenotypes as reported in the main manuscript. The main difference seems to be the activation of `Hypoxia`. As `Hypoxia` is considered an input, it does not have any regulatory factors besides itself. This acts as a switch dividing the two basins in two equal sizes.

The other two minor single state attractors (columns 1 and 5) have a relatively low basin size (around 10%). Their behavior suggests activation of the IL signaling in absence of DNA damage that does not occur in the actual biological system. Since their low basin size, however, the model also suggests that these phenotype would be rarely observed. All other single state attractors have a very low basin size as well and similarly activate IL signaling in absence of DNA damage.

The rest of the basin of tested random states contains attractors of length 3 and 9 with approximately 7.5% each.

```
#load async simulation
load("RSim/attrSim.RData")
#load sync simulation
load("RSim/simulation-basinsize-sasp.RData")
par(mar=c(5,7,5,4))
invisible(plotAttractors(attrs, title = "Basin sizes",
                         offColor = "#7b3294", onColor = "#a6dba0",
                         drawLegend = FALSE))
```

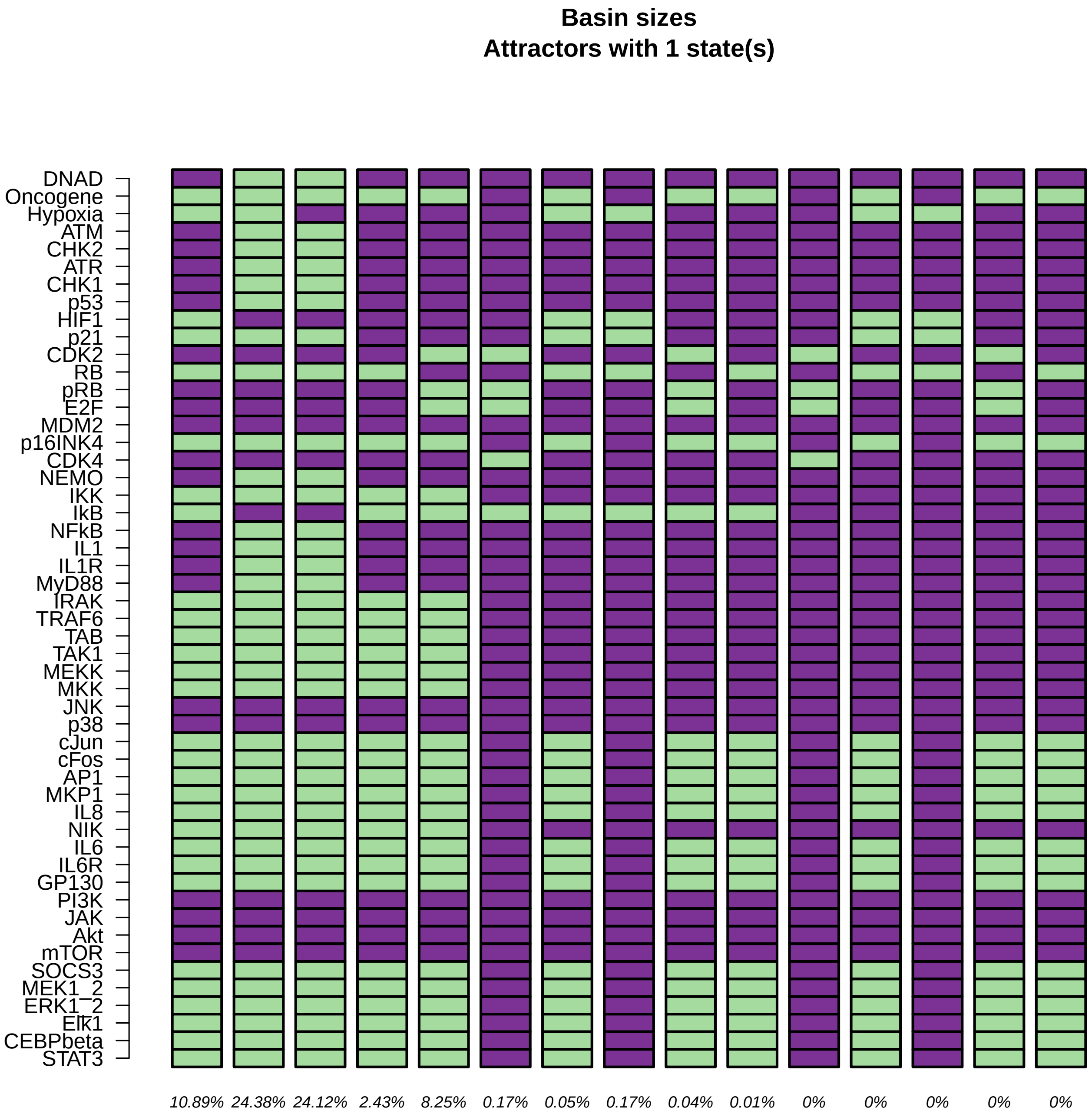
Basin sizes
Attractors with 1 state(s)
DNAD
Oncogene
Hypoxia
ATM
CHK2
ATR
CHK1
p53
HIF1
p21
CDK2
RB
pRB
E2F
MDM2
p16INK4
CDK4
NEMO
IKK
IkB
NFkB
IL1
IL1R
MyD88
IRAK
TRAF6
TAB
TAK1
MEKK
MKK
JNK
p38
cJun
cFos
AP1
MKP1
IL8
NIK
IL6
IL6R
GP130
PI3K
JAK
Akt
mTOR
SOCS3
MEK1_2
ERK1_2
Elk1
CEBPbeta
STAT3
10.89%
24.38%
24.12%
2.43%
8.25%
0.17%
0.05%
0.17%
0.04%
0.01%
0%
0%
0%
0%
0%

Basin sizes
Attractors with 3 state(s)

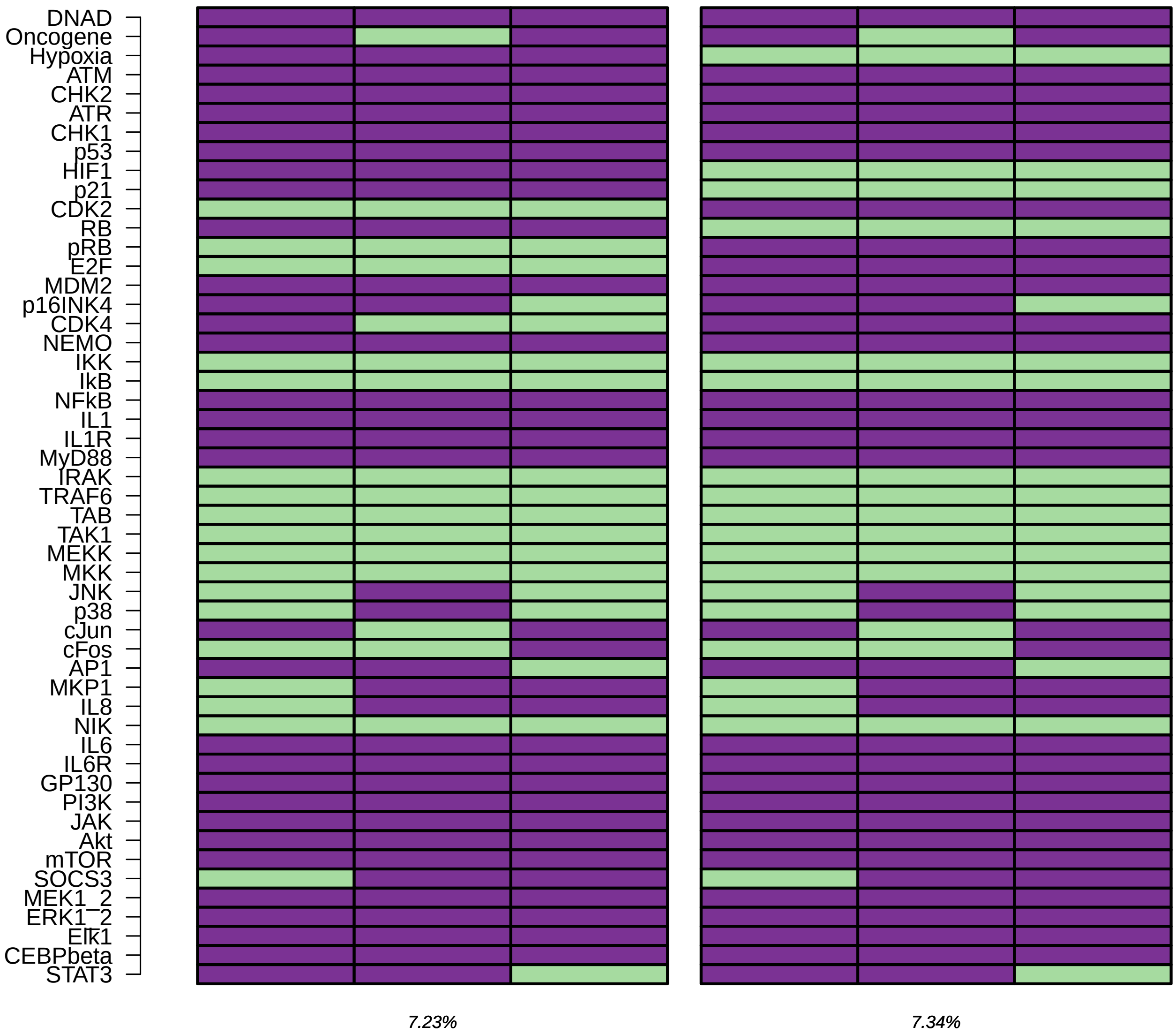

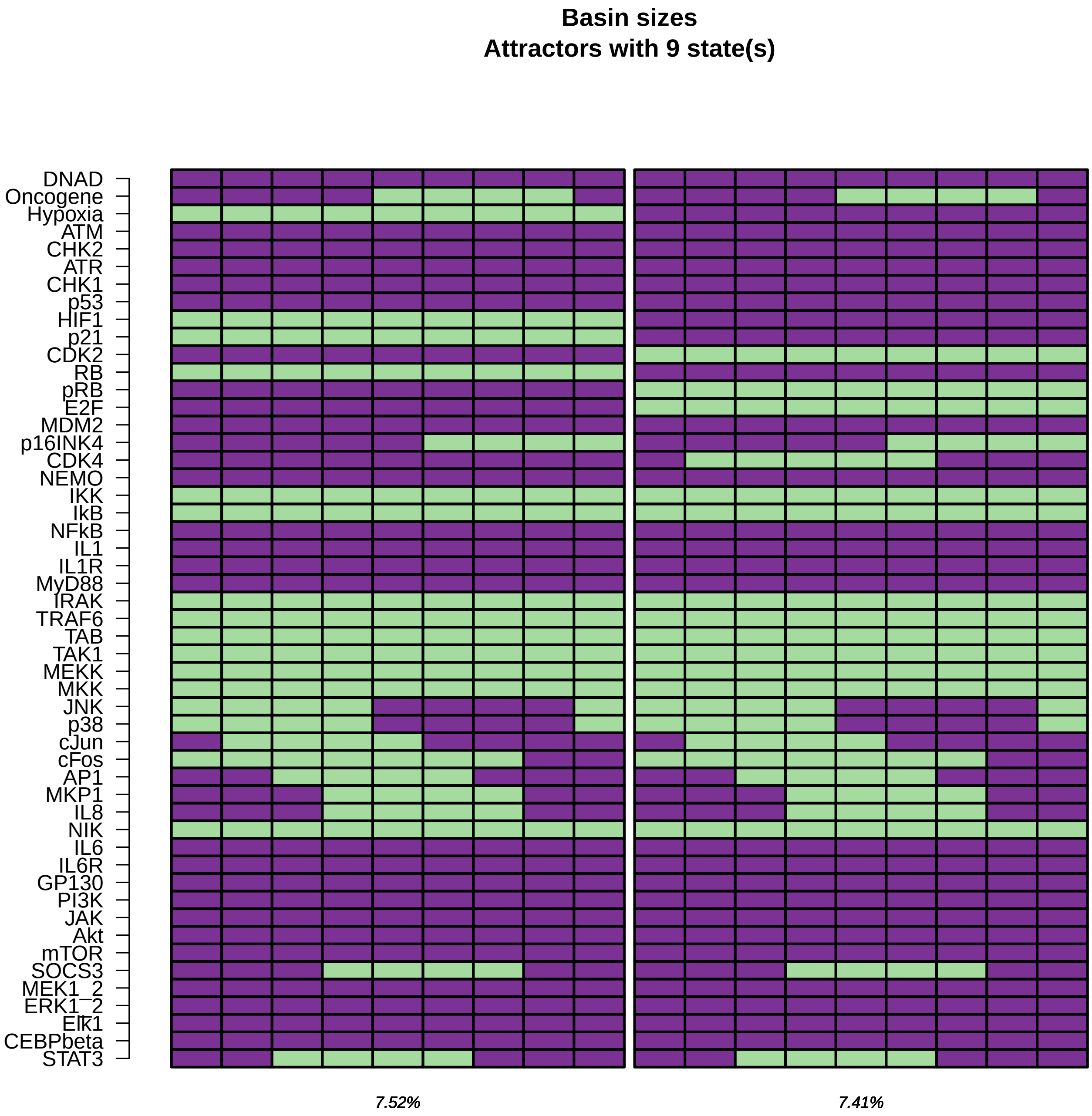

Model predictions using asynchronous updates

Attractors and the trajectories in the state graph might change in the model using asynchronous updates. Our model is mainly focused on synchronous update but in addition we did simulations using an asynchronous update scheme. As for the Basin calculation, we use `5000000` randomly generated initial states for the asynchronous attractor search simulation (`saspAsync <- getAttractors(saspnetwork, type = "asynchronous", startStates = 5000000)`. The resulting attractors are the same 15 steady-state attractors compared to those of the synchronous search. In addition there are two loose attractors found. Using the asynchronous update can lead to different trajectories in comparison to synchronous updates. To validate the stability of our SASP model we add simulations under the same initial conditions as the manuscript (Fig. 2 - Fig. 6) using asynchronous updates. The following plots show that the attractors for the given inputs are independent of the update strategy.

```r
#re-simulate attractor displayed in figure 2 using async updates
startStates <- list(c(c(0,0,0,0,0,0,0,0,0,0,0,0,0,0,1,0,0,0,0,1),rep(0,31)))
#asynchronous attractor search
saspAsyncFig2 <- getAttractors(saspnetwork, type = "asynchronous",
                               startStates = startStates)
par(mar=c(5,7,5,4))
p <- plotAttractors(saspAsyncFig2,
                    title = "Startstate : DNAD = 0, IkB = 1, MDM2 = 1, async",
                    offColor = "#7b3294", onColor = "#a6dba0")
```

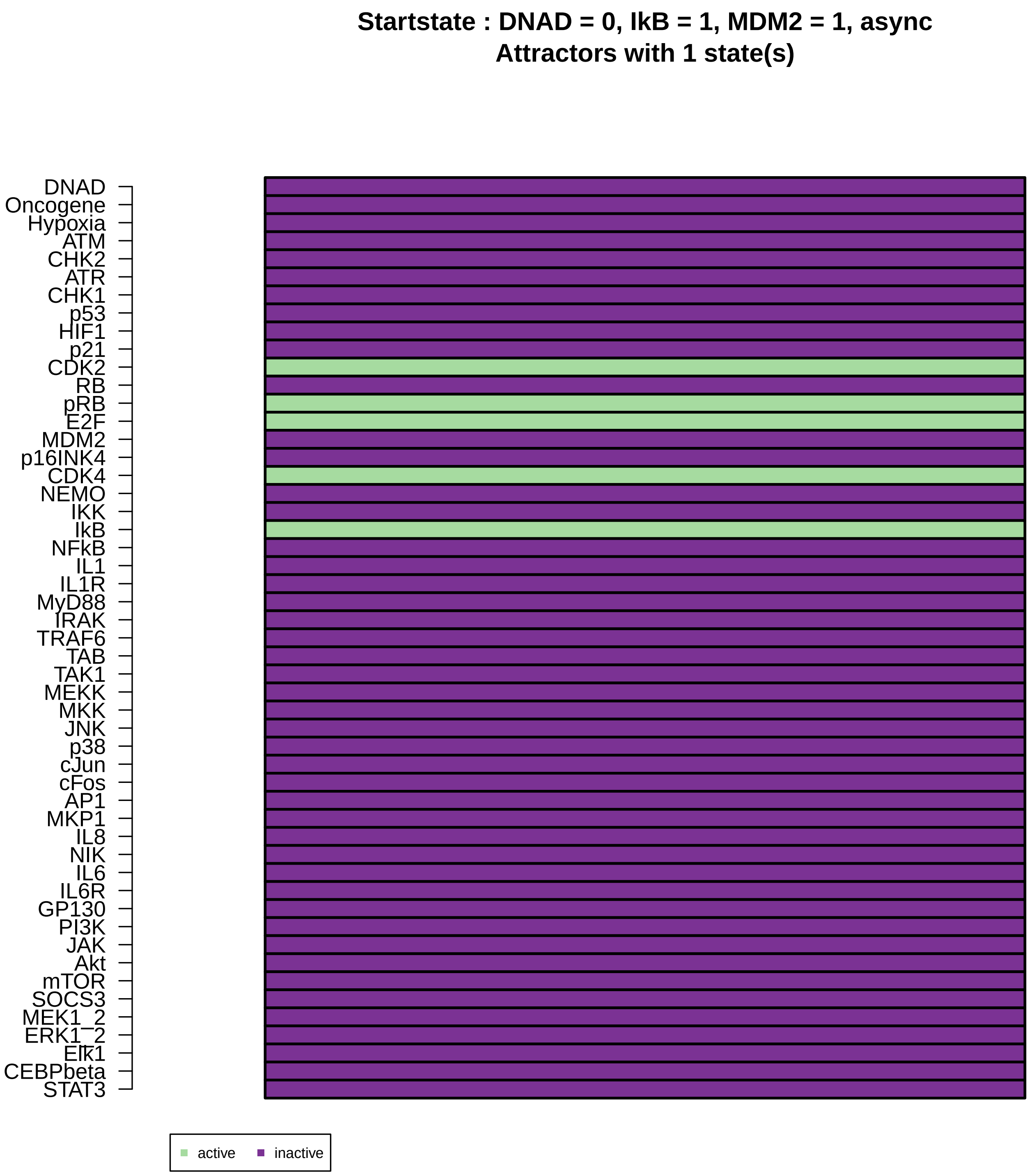


```
#re-simulate attractor displayed in figure 3 using async updates
startStates <- list(c(c(1,0,0,0,0,0,0,0,0,0,0,0,0,0,1,0,0,0,0,1),rep(0,31)))
#asynchronous attractor search
saspAsyncFig3 <- getAttractors(saspnetwork, type = "asynchronous",
                               startStates = startStates)
par(mar=c(5,7,5,4))
p <- plotAttractors(saspAsyncFig3,
                    title = "Startstate : DNAD = 1, IkB = 1, MDM2 = 1, async",
                    offColor = "#7b3294", onColor = "#a6dba0")
```

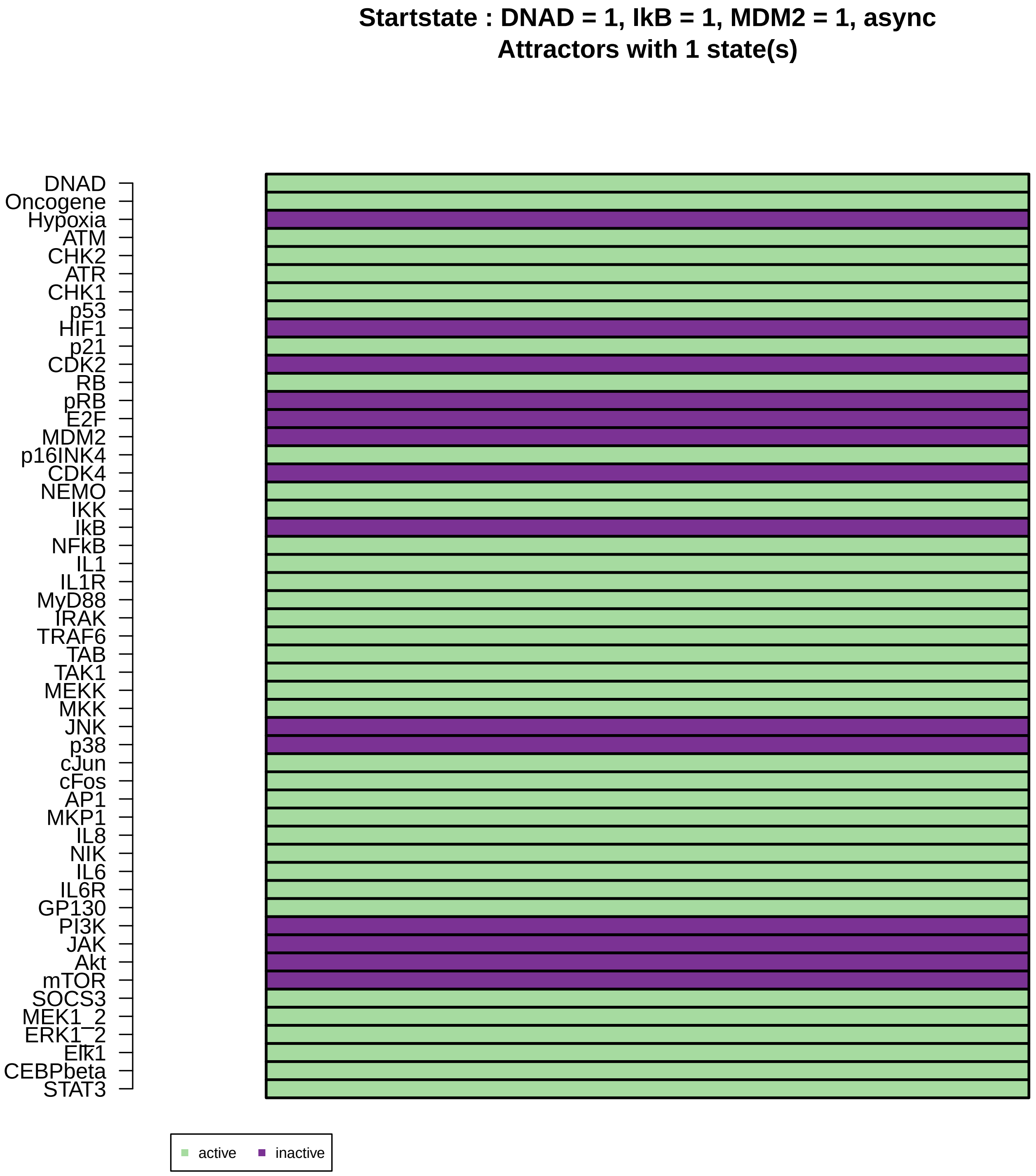


```
#re-simulate attractor displayed in figure 4 using async updates
startStates <- list(c(c(1,0,0,0,0,0,0,0,0,0,0,0,0,0,1,0,0,0,0,1),rep(0,31)))
nfkbpKO <- fixGenes(saspnetwork, "NFkB", 0)
#asynchronous attractor search
saspAsyncFig4 <- getAttractors(nfkbpKO, type = "asynchronous",
                               startStates = startStates)
par(mar=c(5,7,5,4))
p <- plotAttractors(saspAsyncFig4,
                    title = "Startstate : DNAD = 1, IkB = 1, MDM2 = 1, NFkB knock out,  async",
```

```
                        offColor = "#7b3294", onColor = "#a6dba0")
```

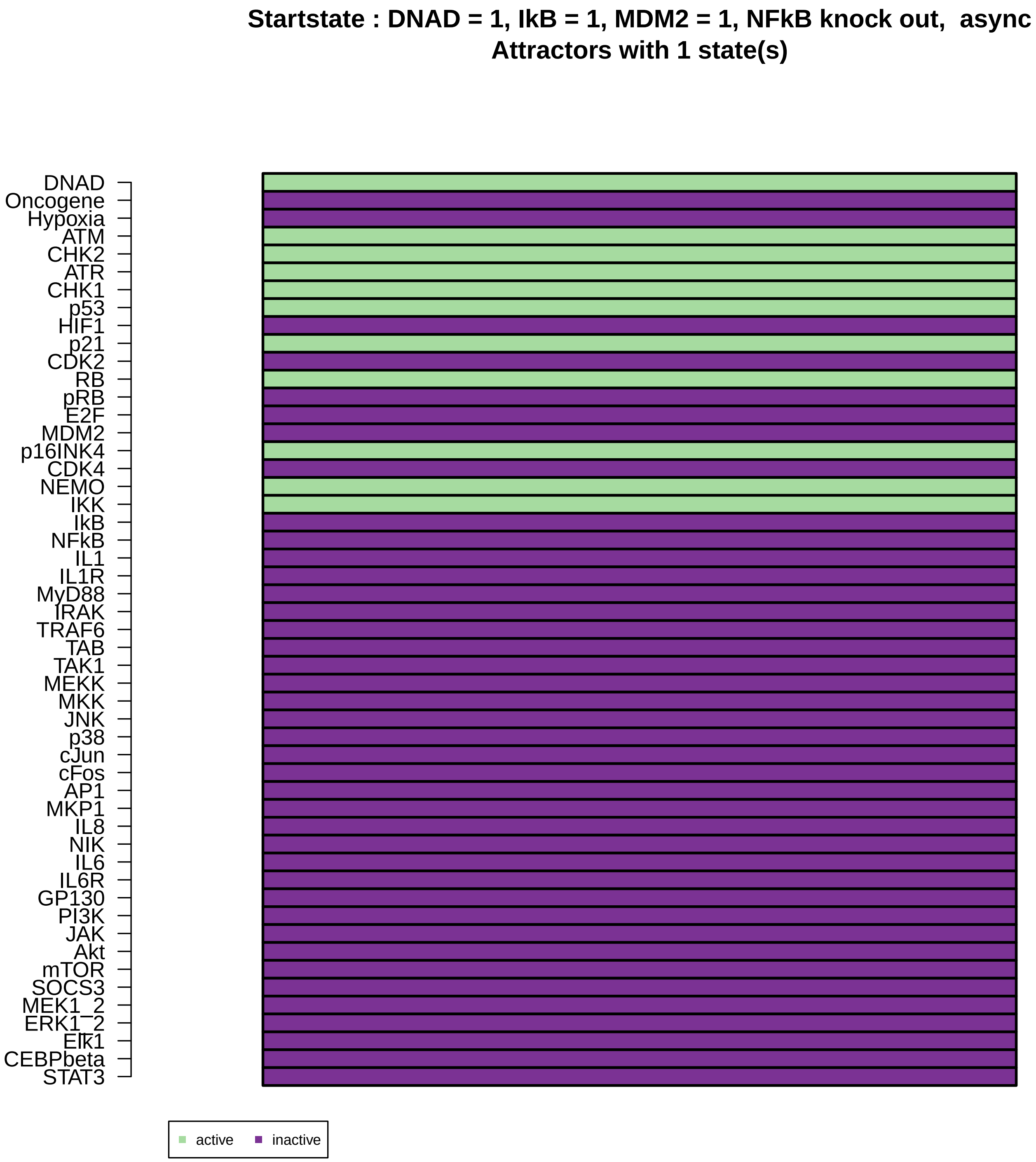


```
#re-simulate attractor displayed in figure 5 using async updates
startStates <- list(c(c(1,0,0,0,0,0,0,0,0,0,0,0,0,0,1,0,0,0,0,1),rep(0,31)))
ikbKO <- fixGenes(saspnetwork, "IkB", 1)
#asynchronous attractor search
saspAsyncFig5 <- getAttractors(ikbKO, type = "asynchronous",
                               startStates = startStates)
par(mar=c(5,7,5,4))
```

```r
p <- plotAttractors(saspAsyncFig5,
                    title = "Startstate : DNAD = 1, IkB = 1, MDM2 = 1, IkB over-expression, async",
                    offColor = "#7b3294", onColor = "#a6dba0")
```

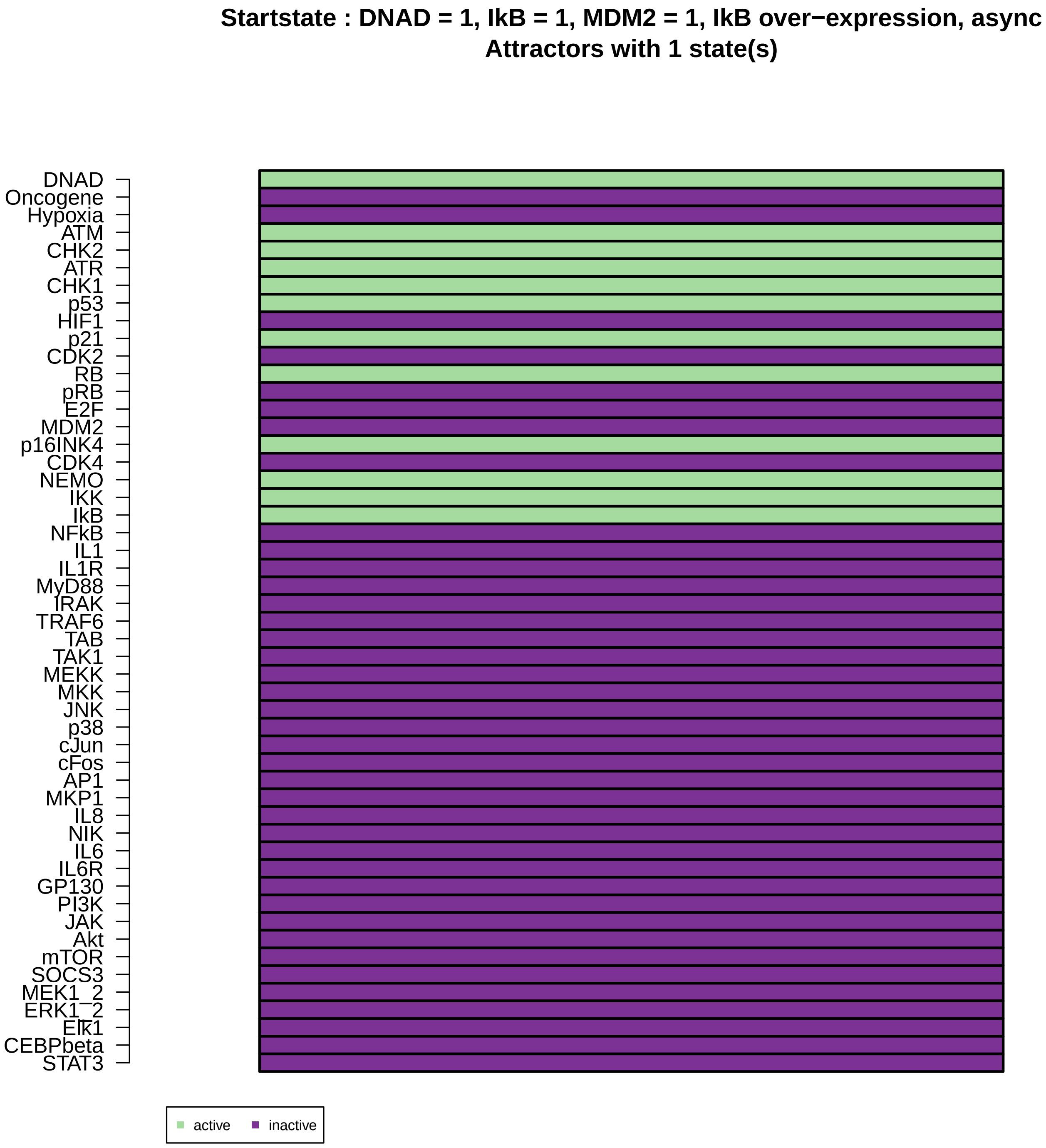


```r
#re-simulate attractor displayed in figure 6 using async updates
startStates <- list(c(c(1,0,0,0,0,0,0,0,0,0,0,0,0,0,1,0,0,0,0,1),rep(0,31)))
nemoKO <- fixGenes(saspnetwork, "NEMO", 0)
#asynchronous attractor search
saspAsyncFig6 <- getAttractors(nemoKO, type = "asynchronous",
                               startStates = startStates)
```

```r
par(mar=c(5,7,5,4))
p <- plotAttractors(saspAsyncFig6,
                    title = "Startstate : DNAD = 1, IkB = 1, MDM2 = 1, NEMO knock-out, async",
                    offColor = "#7b3294", onColor = "#a6dba0")
```

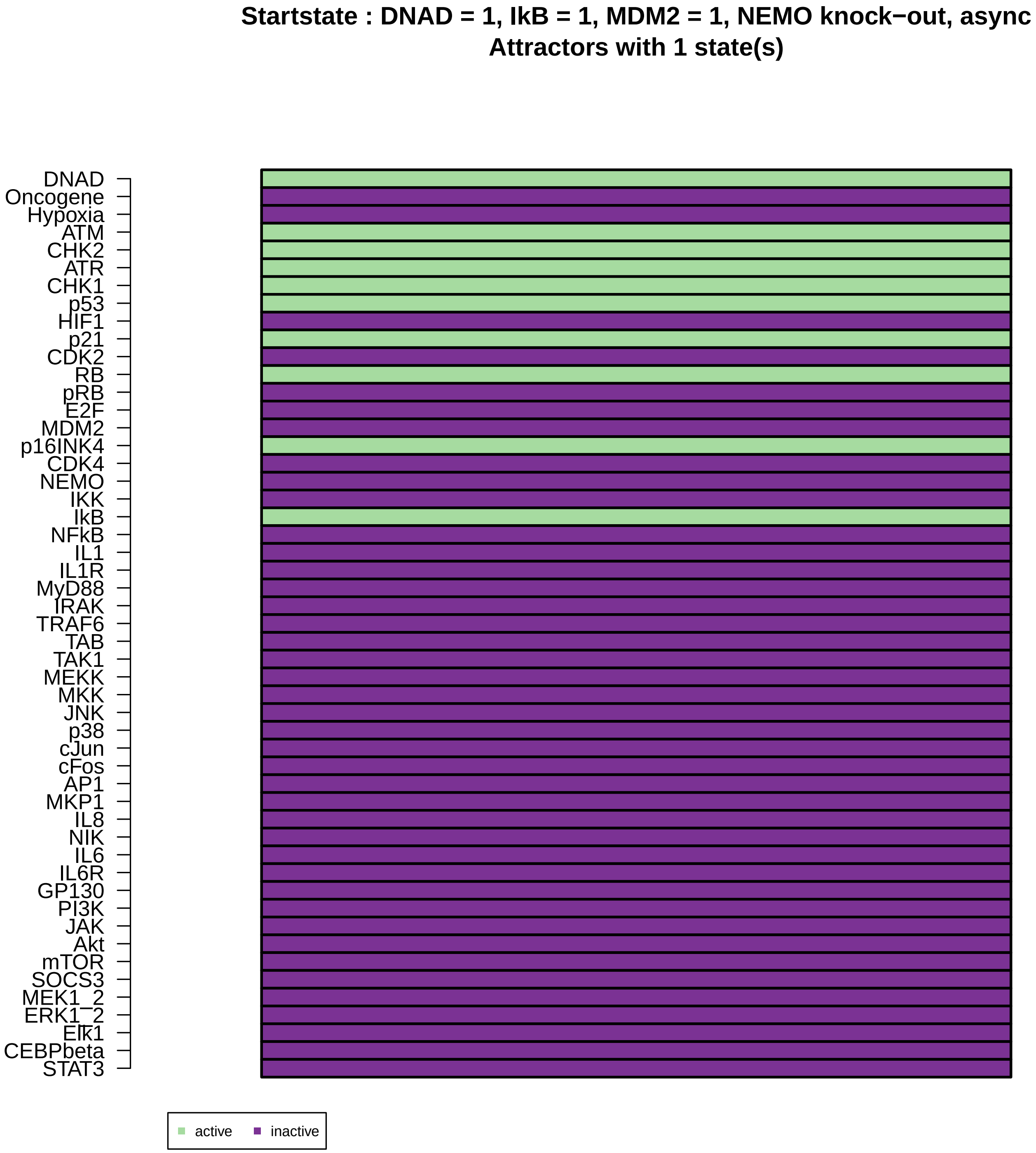

The complex attractors that are found in the attractor search comprise 704 and 1408 states respectively. We aimed at a possible interpretation of these attractors by searching for the stable conditions inside the attractors. We measure the probability of each regulatory factor to be active inside the complete attractors. All regulatory factors that are either `0` or `1` in the complete set of attractors states are extracted. The

constant regulatory factors inside the attractor are used to create a so called symbolic steady state attractor. This symbolic steady state attractor is used to improve the interpretability of these large complex attractors.

```
#reduce attractors based
reducedAttrs <- computeSymbolicSteadyStates(saspAsync, round = F)
#format attractors to single attractor objects to be able to plot them using BoolNet
reformattedReducedAttrs <- formatReducedAttractors(reducedAttrs = reducedAttrs,
                                                    attrs = saspAsync)

#extract loose attractors only
idx <- sapply(saspAsync$attractors, function(a) ncol(a$involvedStates) > 1)
origIdx <- which(idx)
reducedAttrs <- reducedAttrs[idx]
reformattedReducedAttrs <- reformattedReducedAttrs[idx]

#print symbolic steady states
for(i in 1:length(reducedAttrs))
{
    print(paste("Attractor No. " , i, sep =""))
    print(paste("original length : ", ncol(saspAsync$attractors[[origIdx[i]]]$involvedStates), sep =""))
    print("Probabilities:")
    print(reducedAttrs[[i]]$percentageOn)
    print(paste("number of constant genes in attractor: ",
                length(reformattedReducedAttrs[[i]]$stateInfo$genes)))
}

#plot "steady state" attractors
for(i in 1:length(reducedAttrs))
{
  par(mar=c(5,7,5,4))
  plotAttractors(reformattedReducedAttrs[[i]],
                 title = paste("Fixed-state attractor from loose attractor ", i),
                 offColor = "#7b3294", onColor = "#a6dba0")
}
```

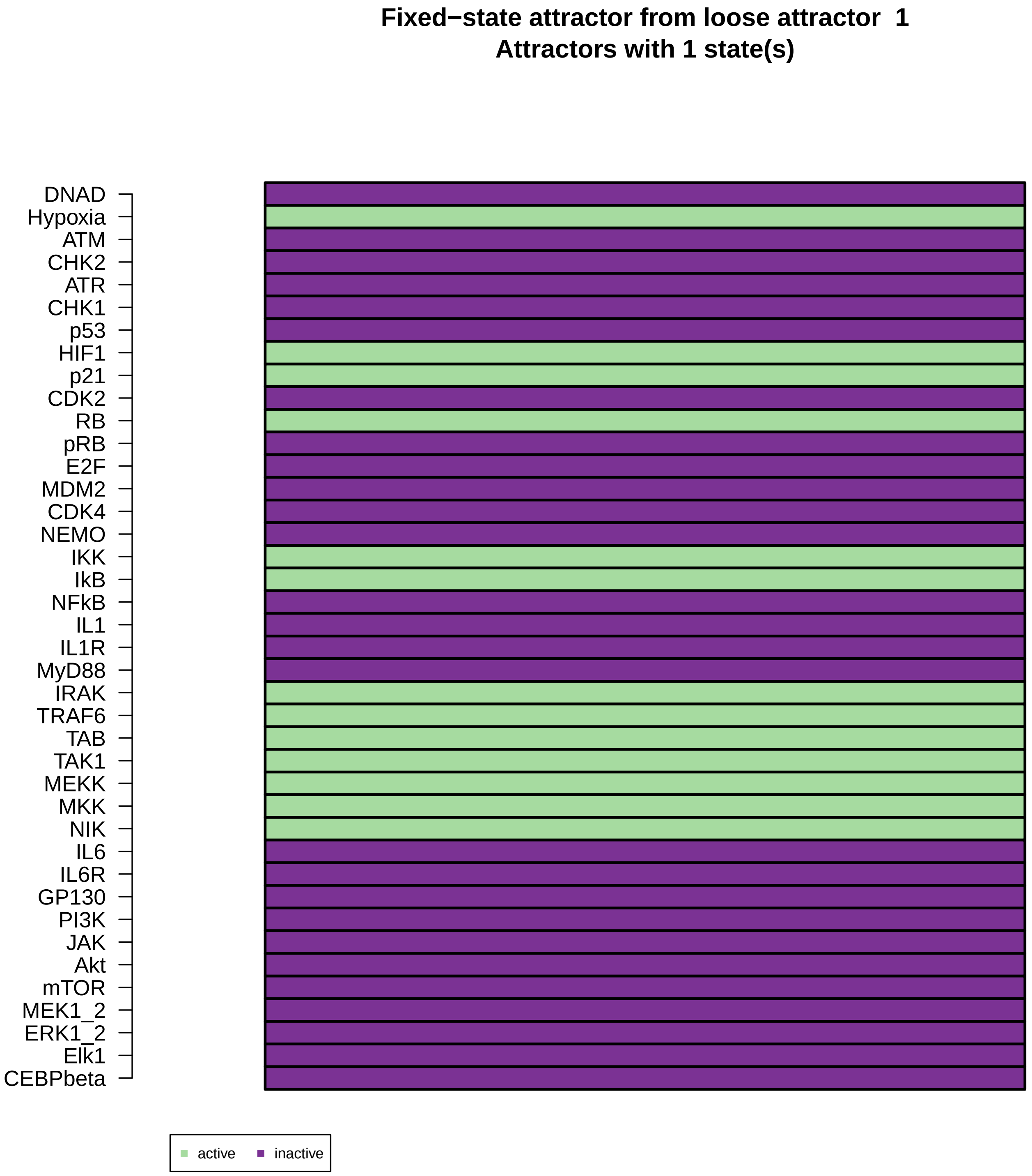
Fixed−state attractor from loose attractor 1
Attractors with 1 state(s)
DNAD
Hypoxia
ATM
CHK2
ATR
CHK1
p53
HIF1
p21
CDK2
RB
pRB
E2F
MDM2
CDK4
NEMO
IKK
IkB
NFkB
IL1
IL1R
MyD88
IRAK
TRAF6
TAB
TAK1
MEKK
MKK
NIK
IL6
IL6R
GP130
PI3K
JAK
Akt
mTOR
MEK1_2
ERK1_2
Elk1
CEBPbeta
active
inactive

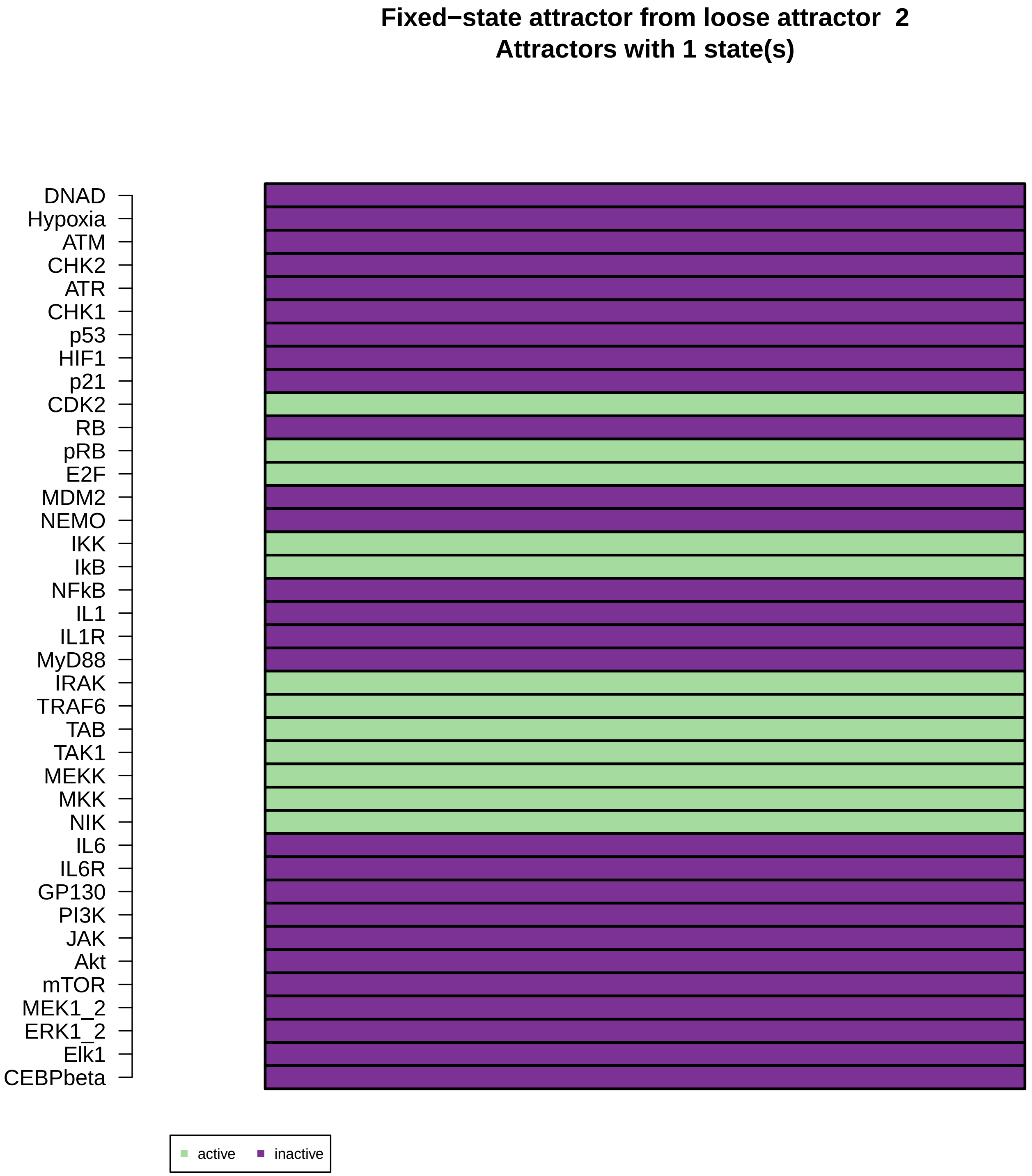


```
[1] "Attractor No. 1"
[1] "original length : 704"
[1] "Probabilities:"
     DNAD  Oncogene   Hypoxia       ATM      CHK2       ATR      CHK1
0.0000000 0.5000000 1.0000000 0.0000000 0.0000000 0.0000000 0.0000000
      p53      HIF1       p21      CDK2        RB       pRB       E2F
0.0000000 1.0000000 1.0000000 0.0000000 1.0000000 0.0000000 0.0000000
     MDM2   p16INK4      CDK4      NEMO       IKK       IkB      NFkB
0.0000000 0.5000000 0.0000000 0.0000000 1.0000000 1.0000000 0.0000000
```

```
      IL1      IL1R     MyD88      IRAK     TRAF6       TAB      TAK1
0.0000000 0.0000000 0.0000000 1.0000000 1.0000000 1.0000000 1.0000000
     MEKK       MKK       JNK       p38      cJun      cFos       AP1
1.0000000 1.0000000 0.4545455 0.4545455 0.5909091 0.7272727 0.4090909
     MKP1       IL8       NIK       IL6      IL6R     GP130      PI3K
0.3636364 0.5000000 1.0000000 0.0000000 0.0000000 0.0000000 0.0000000
      JAK       Akt      mTOR     SOCS3    MEK1_2    ERK1_2      Elk1
0.0000000 0.0000000 0.0000000 0.5000000 0.0000000 0.0000000 0.0000000
 CEBPbeta     STAT3
0.0000000 0.3636364
[1] "number of constant genes in attractor:  40"
[1] "Attractor No. 2"
[1] "original length : 1408"
[1] "Probabilities:"
     DNAD  Oncogene   Hypoxia       ATM      CHK2       ATR      CHK1
0.0000000 0.5000000 0.0000000 0.0000000 0.0000000 0.0000000 0.0000000
      p53      HIF1       p21      CDK2        RB       pRB       E2F
0.0000000 0.0000000 0.0000000 1.0000000 0.0000000 1.0000000 1.0000000
     MDM2   p16INK4      CDK4      NEMO       IKK       IkB      NFkB
0.0000000 0.5000000 0.5000000 0.0000000 1.0000000 1.0000000 0.0000000
      IL1      IL1R     MyD88      IRAK     TRAF6       TAB      TAK1
0.0000000 0.0000000 0.0000000 1.0000000 1.0000000 1.0000000 1.0000000
     MEKK       MKK       JNK       p38      cJun      cFos       AP1
1.0000000 1.0000000 0.4545455 0.4545455 0.5909091 0.7272727 0.4090909
     MKP1       IL8       NIK       IL6      IL6R     GP130      PI3K
0.3636364 0.5000000 1.0000000 0.0000000 0.0000000 0.0000000 0.0000000
      JAK       Akt      mTOR     SOCS3    MEK1_2    ERK1_2      Elk1
0.0000000 0.0000000 0.0000000 0.5000000 0.0000000 0.0000000 0.0000000
 CEBPbeta     STAT3
0.0000000 0.3636364
[1] "number of constant genes in attractor:  39"
```

Looking at the constant factors of the two loose attractors, both suggest an activation of the IL-1 signaling in absence of DNA damage. This does not occur in the biological system. In addition, the large sizes of the attractors (704,1408) suggests that these attractors do not represent a biological phenotype.
